# Extensive Quantitative Profiling of Plasma Steroidome and Prostaglandin Metabolites for Studies of Female Reproductive Physiology

**DOI:** 10.1021/acs.analchem.6c03626

**Published:** 2026-09-02

**Authors:** Haolin Chen, Luca Bürgi, Anna-Katharina Hankele, Reto Giacometti, Susanne E. Ulbrich, Laurent Bigler

**Affiliations:** † 27219 ETH Zurich, Animal Physiology, Institute of Agricultural Sciences, Universitaetstrasse 2, Zurich 8092, Switzerland; ‡ 27217 University of Zurich, Department of Chemistry, Winterthurerstrasse 190, Zurich 8057, Switzerland

## Abstract

Steroid hormones and their metabolites orchestrate female reproductive physiology. Key analytes such as 17β-estradiol and progesterone are integral to standard clinical practice worldwide. For more advanced endocrine monitoring of female reproduction, current analytical approaches lack automated throughput, broad metabolite coverage, and sufficient sensitivity. Herein, we present an ultrahigh-performance liquid chromatography coupled with high-resolution mass spectrometry (UHPLC-HRMS) workflow that targets 56 selected steroids (estrogens, progestogens, androgens, glucocorticoids, and steroid conjugates) and 5 prostaglandin metabolites for quantitative profiling using 250 μL of plasma within a single pipeline. The workflow integrates optimized, automated protein precipitation-enhanced solid-phase extraction and two complementary chromatographic separations with tailored eluent additives and ionization modes to accommodate the chemical diversity of the broad target panel. A full method validation demonstrated excellent performance, with low limits of quantification (5–200 pg/mL) determined in charcoal-stripped plasma as a surrogate matrix. The workflow was applied to authentic cattle (n = 5) and human (n = 18) plasma samples from males and, particularly, females across the female reproductive cycle and selected pregnancy stages. Both well-established endocrine hormones and their metabolites, as well as analytes whose roles in reproduction remain insufficiently characterized, were among the 42 and 48 targets detected in cattle and human samples, respectively. Compound-specific, valley-split, and composite quantification were reported separately. This demonstrates extensive target coverage and robust quantitative performance across biological matrices with distinct physiological contexts. The workflow thus offers a robust tool for steroidomic studies and enables broad-spectrum monitoring of endocrine dynamics for basic, translational, and clinical research.

## Graphical abstract

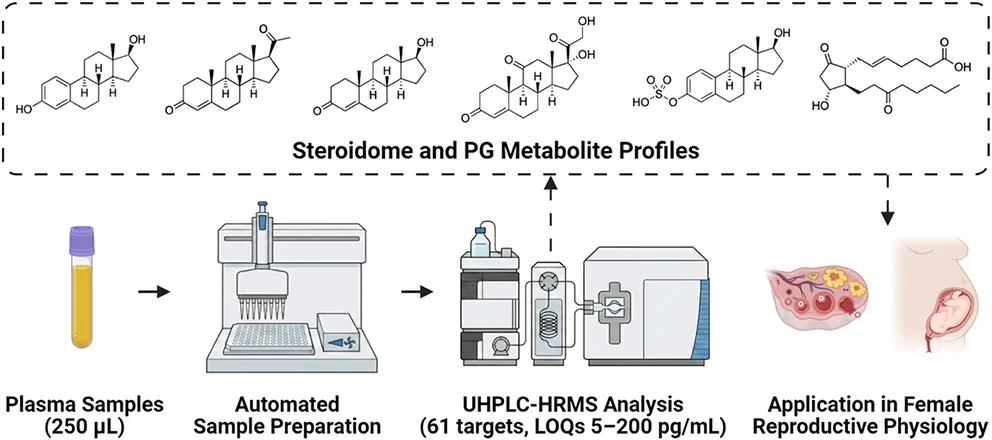

## Introduction

Steroid hormones play a pivotal role in physiology, governing sexual development and reproductive capacity, and impacting overall health. Even minor imbalances in hormone levels can profoundly perturb fertility, resulting in difficulties in conceiving or maintaining an ongoing pregnancy.^1,2^ The sex steroids estrogens, progestogens, and androgens are particularly critical. Both excess and shortage, as well as dysregulated production and metabolism of these steroids are associated with impaired fertility.^3^ Common disorders that disrupt the menstrual cycle include polyendocrine metabolic ovarian syndrome (PMOS), which affects one in eight women worldwide, and premature ovarian insufficiency (POI), which occurs in 3.5% of women under the age of 40.^4,5^ Furthermore, lifestyle factors such as stress and obesity can significantly impact the menstrual cycle, manifesting in altered cycle length and hormonal imbalances.^6,7^

A normal menstrual cycle is governed by the tightly regulated and highly complex interplay of multiple hormones. Increasing evidence indicates that, in addition to classical steroid hormones, bioactive steroid metabolites act as crucial mediators of physiological and pathophysiological changes during the menstrual cycle.^8–10^ To date, their specific contribution to female reproduction remains insufficiently understood. Due to technical limitations, most studies on female reproduction and routine endocrinological assessments in reproductive medicine, have so far focused on a few major steroids including 17β-estradiol (E2) and progesterone (P4).^11^ For instance, E2, the most potent estrogen, is widely used in *in vitro* fertilization (IVF) clinics as a prognostic marker for treatment success, although its validity for the latter remains controversial.^12^ So far, assessing the metabolic profiles of steroids—the steroidome—has hardly been introduced to research or clinical settings. Sensitive, accurate, and precise quantification of more numerous steroid hormones, their precursors, and bioactive metabolites is of interest for both research and clinical diagnosis of female reproductive disorders affecting fertility and health. Thus, expanding from measurements of single steroids to extensive steroidome profiling allows the exploration of a broader spectrum of hormones governing reproductive processes, providing deeper insights into endocrine physiology and pathology.

Mass spectrometry-based approaches represent a valuable resource to replace antibody-based assays as the gold standard for steroid quantification.^13^ In particular, liquid chromatography–mass spectrometry (LC-MS) offers multiplexing capabilities along with superior specificity and sensitivity compared with conventional immunoassays.^14^ The characterization and quantification of the steroidome using LC-MS has been successfully applied in studies of toxicology, cancer pathology, and doping control, providing a deeper understanding of their molecular mechanisms and facilitating the development of more robust identification strategies.^15–17^ To date, the research on steroidome dynamics across female reproductive stages remains limited. In addition to steroids, prostaglandins (PG) and their metabolites contribute to reproductive processes, such as ovulation, luteolysis, uterine contractility, and parturition, but have not yet been included in current plasma steroidomic studies.^15,18,19^ Although a recently published method combined the screening of steroids and PG metabolites from urinary matrices,^20^ the accurate quantification of such clinically relevant targets in plasma would require an optimized sample preparation procedure and enhanced analytical sensitivity. These limitations have restricted the comprehensive characterization of steroid and PG metabolites relevant to female reproductive physiology. To dismantle these barriers, we developed an ultrahigh-performance liquid chromatography coupled with high-resolution mass spectrometry (UHPLC-HRMS) workflow to quantitatively analyze 61 targets, including estrogens, progestogens, androgens, glucocorticoids, steroid conjugates, and PG metabolites. The panel was designed to extensively capture well-established steroids, selected downstream steroid metabolites, and PG metabolites associated with female reproductive physiology (Figure 1). For many of these targets, their role in female reproduction has not been fully elucidated. Through systematic optimization of automated sample pretreatment, chromatographic separation, and mass spectrometric detection, we aimed at achieving low limits of quantification in the pg/mL range using the lowest possible amount of plasma. To demonstrate its applicability in biologically relevant matrices, we validated the workflow using authentic plasma samples from humans and cattle, which are characterized by highly variable steroidome compositions as they originate from different physiological states. Overall, this study not only aimed to fill technical gaps in steroidome profiling, but also to provide a powerful tool for elucidating hormone-related pathology and physiology to advance research in female reproductive health.

**Figure 1:**
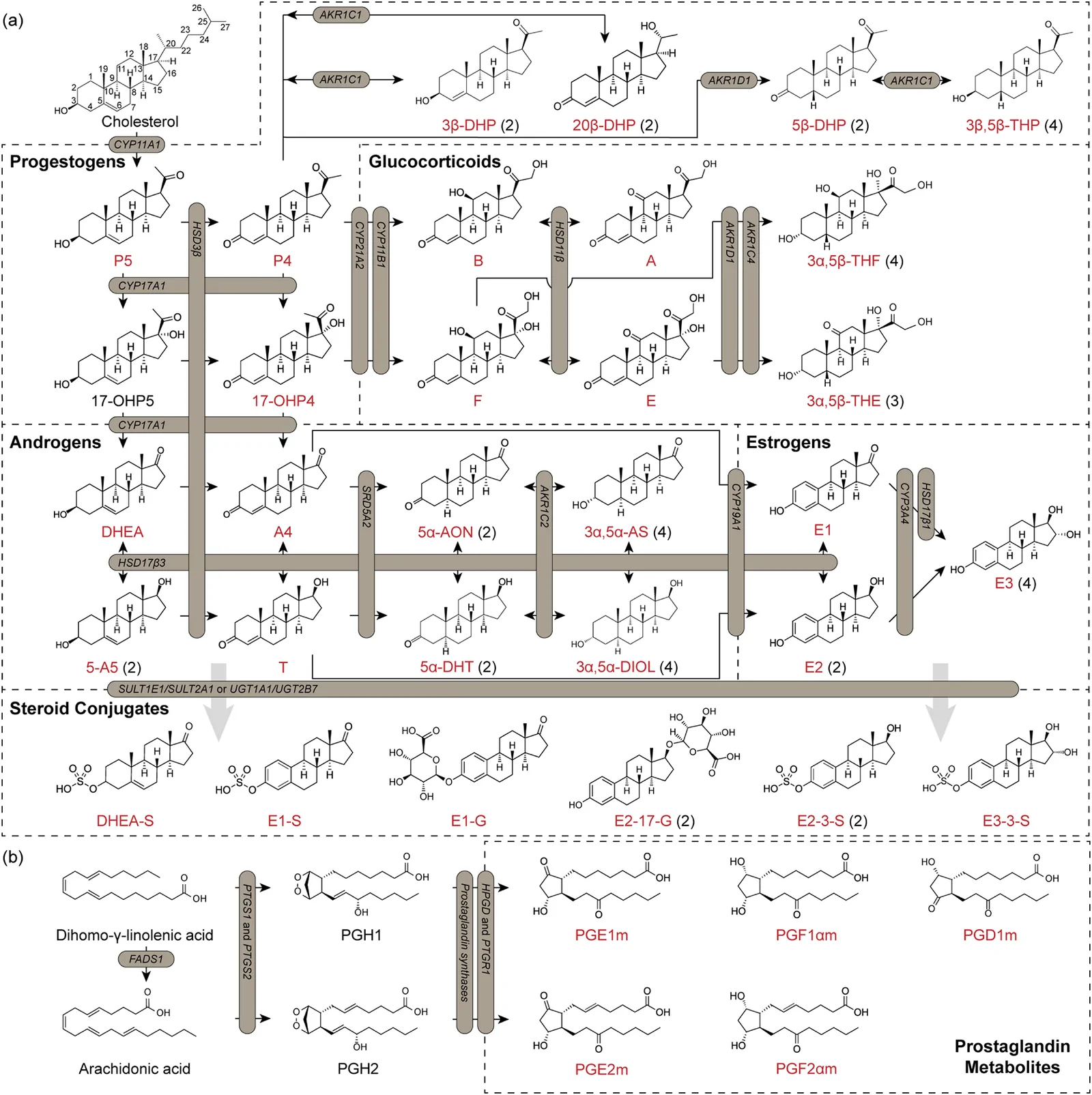
Biosynthetic pathways of (a) steroids and (b) PG metabolites. The target analytes investigated in this study are highlighted in red and enclosed in dashed boxes, categorized into estrogens, progestogens, androgens, glucocorticoids, steroid conjugates and PG metabolites. For stereoisomeric steroids, only one representative chemical structure is shown, with the number of isomers indicated in parentheses following the compound abbreviation. The target panel included 61 analytes; see Table S1 for the complete list including full names and abbreviations.

## Experimental Section

### Plasma Sampling

Plasma samples (n = 5 in total) were collected from five cattle, namely during the follicular phase (n = 1), the luteal phase (n = 1), the sixth month of pregnancy (n = 1), 16 min postpartum (n = 1), and from an adult bull (n = 1). The study was approved by the Ethical Committee of Animal Experiments, Canton of Zurich, Switzerland (ZH083/2021), and carried out following accepted standards in humane animal care. In addition, plasma samples at comparable reproductive stages (n = 18 in total) were obtained by voluntary self-donation from two adult authors of this study with informed consent for analytical method evaluation. These included samples from one adult woman of reproductive age during the follicular phase (n = 3), the luteal phase (n = 4), at ovulation (n = 5), and during pregnancy (n = 3), as well as samples from one adult man (n = 3). Whole blood samples were collected using Vacutainer plastic K2EDTA tubes (Dickinson AG, Allschwil, Switzerland) and centrifuged at 2000 × *g* for 20 min at 4 °C. The plasma supernatants were then transferred separately to 15 mL centrifuge tubes, aliquoted into 96-well plates, and stored at −20 °C until sample preparation.

### Target Analytes

In total, 61 analytes were included and grouped into six categories. Estrogens included E1; E2; 17α-E2; E3; 16β,17α-E3; 16β,17β-E3; and 16α,17α-E3. Progestogens included P5; P4; 17-OHP4; 3α-DHP; 3β-DHP; 5α-DHP; 5β-DHP; 20α-DHP; 20β-DHP; 3β,5α-THP; 3β,5β-THP; 3α,5β-THP; and 3α,5α-THP. Androgens included DHEA; 5-A5; 4-A5; A4; T; 5α-AON; 5β-AON; 5α-DHT; 5β-DHT; 3β,5α-AS; 3β,5β-AS; 3α,5β-AS; 3α,5α-AS; 3β,5α-DIOL; 3β,5β-DIOL; 3α,5β-DIOL; and 3α,5α-DIOL. Glucocorticoids included B; F; A; E; 3β,5α-THF; 3β,5β-THF; 3α,5α-THF; 3α,5β-THF; 3β,5α-THE; 3α,5α-THE; and 3α,5β-THE. Steroid conjugates included E1-S; E2–17-S; E2–3-S; E3–3-S; DHEA-S; E1-G; E2–3-G; and E2–17-G. PG metabolites included PGE2m; PGF2αm; PGD1m; PGE1m; and PGF1αm. For target quantification, an isotopically labeled internal standard (ISTD) was assigned to each analyte based on structural similarity within the respective chemical class. The full names and correspondence between targets and ISTDs are presented in Table S1, and the ISTDs are listed in Table S2.

### Standard Solutions

The preparation of charcoal-stripped plasma as a surrogate matrix for spiking experiments was adapted from our previous research,^21^ as detailed in the Supporting Information. Stock solutions of the 61 target standards (STDs) and 13 ISTDs were individually prepared in methanol (MeOH) at a concentration of 200 μg/mL (Tables S1 and S2). Mixed STD and ISTD solutions were then prepared in MeOH at a concentration of 2 μg/mL. All stock and mixed solutions were stored at −20 °C. On the day of sample preparation, STD working solutions (5, 50, and 500 ng/mL) and an ISTD working solution (50 ng/mL) were freshly prepared by diluting the corresponding mixed solutions with MeOH. Serial calibrators (1–25000 pg/mL) and quality control (QC) samples (400, 1600, and 10000 pg/mL) were prepared by spiking charcoal-stripped plasma with the STD working solutions.

### Sample Preparation

An automated sample preparation was performed in 96-well plates using an Andrew+ pipetting robot (Andrew Alliance, Vernier, Switzerland), the details of the system configuration are described in the Supporting Information. Each well received 250 μL of calibrators, QCs, or authentic plasma samples, followed by 10 μL of ISTD working solution. The plate was then shaken at 2000 rpm for 5 min at 4 °C.

For protein digestion, 5 μL of proteinase K (20 mg/mL) and 5 μL of 0.25 M CaCl_2_ were added to each well. The plate was incubated at 37 °C with shaking at 2000 rpm for 15 min. Protein precipitation was achieved by adding 125 μL of 0.1 M ZnSO_4_ and 250 μL of cold MeOH, followed by shaking at 1200 rpm for 10 min at 4 °C and centrifugation at 3220 × *g* for 10 min at 4 °C. The supernatant (375 μL) was collected from each well and diluted with 225 μL of H_2_O. An Oasis HLB 96-well solid-phase extraction plate (10 mg sorbent per well, Waters, Dättwil, Switzerland) was processed using a Biotage PRESSURE+ positive pressure manifold (Biotage GB Limited, Hengoed, UK). The plate was conditioned with 500 μL of acetonitrile (MeCN) and equilibrated with 500 μL of H_2_O. The diluted supernatant was loaded onto the extraction plate, which was then washed with 500 μL of H_2_O and followed by 500 μL of 5% MeCN. Elution was performed using 500 μL of MeCN. The eluent was evaporated under a N_2_ stream at 45 °C, and the resulting dried extracts were stored at −20 °C until analysis.

Prior to analysis, dried extracts were reconstituted at 4 °C in 100 μL of MeOH/H_2_O (2:3, v/v) by shaking at 2000 rpm for 5 min. The reconstituted samples were transferred to the autosampler (maintained at 4 °C) for UHPLC-HRMS analysis. Liquid–liquid extraction as an alternative sample preparation procedure is described in the Supporting Information.

### UHPLC-HRMS Analysis

The samples were analyzed on a Dionex Ultimate 3000 UHPLC system coupled to a Q Exactive hybrid quadrupole-Orbitrap mass spectrometer (Thermo Fisher, Waltham, MA, USA) equipped with a heated electrospray ionization (ESI) source. The UHPLC system comprised a binary RS pump, an XRS open autosampler (CTC, Zwingen, Switzerland), and a temperature-controlled RS column compartment.

Each sample sequence was measured twice using a two-injection strategy based on the two chromatographic methods described below (*Injection 1* and *Injection 2*). When switching methods, the mobile and stationary phases were modified along the same flow path, the system was purged, the column was equilibrated, and blanks were injected. The injection volume was always 15 μL. *Injection 1*—Estrogens were separated at 60 °C using an ACQUITY Premier BEH C18 column (1.7 μm, 2.1 × 100 mm, Waters, Milford, MA, USA) with a precolumn (1.7 μm, 2.1 × 5 mm, Waters). The mobile phases were (A) H_2_O with 0.1% ammonium hydroxide (NH_4_OH) and (B) MeOH with 0.1% NH_4_OH, at a flow rate of 0.45 mL/min. The gradient program was: 40% B (0–0.5 min), linear increase to 60% B (0.5–7.0 min), to 100% B (7.0–7.1 min), isocratic at 100% B (7.1–11.0 min), decrease to 40% B (11.0–11.1 min), and isocratic at 40% B (11.1–13.0 min). *Injection 2*—Steroid conjugates, glucocorticoids, PG metabolites, androgens, and progestogens were separated at 35 °C using an ACQUITY UPLC HSS T3 column (1.8 μm, 2.1 × 100 mm) with a precolumn (1.8 μm, 2.1 × 5 mm). The mobile phases were (A) H_2_O with 0.1% formic acid (HCOOH) and (B) MeCN with 0.1% HCOOH, at a flow rate of 0.50 mL/min. The gradient program was: 28% B (0–0.5 min), linear increase to 75% B (0.5–10.0 min), to 100% B (10.0–10.1 min), isocratic at 100% B (10.1–14.0 min), decrease to 28% B (14.0–14.1 min), and isocratic at 28% B (14.1–16.0 min).

The mass spectrometric detection was performed in full-scan mode at a resolution of 70000 full width at half-maximum (fwhm) at *m*/*z* 200, with an AGC target of 10^6^ and a maximum injection time of 247 ms according to the parameters described in our previous study.^21^ *Injection 1*—Estrogens were analyzed in (−)-ESI mode at a spray voltage of −3000 V in a mass range between *m*/*z* 260 and 300. The capillary and auxiliary gas heater temperatures were set to 265 and 425 °C, respectively. *Injection 2*—Steroid conjugates and glucocorticoids were analyzed in (−)-ESI mode (−3500 V) from 0.0 to 4.5 min, followed by a polarity switch to (+)-ESI mode (+3500 V) for PG metabolites, androgens, and progestogens from 4.5 to 16.0 min in a mass range between *m*/*z* 240 and 500. The capillary and auxiliary gas heater temperatures were set to 265 and 460 °C, respectively. Sheath, auxiliary, and sweep gas flow rates were fixed at 30, 14, and 6 (arbitrary units) for all measurements. External mass calibration with Pierce ESI negative and positive ion solutions (Thermo Fisher, Waltham, MA, USA) ensured mass accuracies below 2 ppm.

### Method Validation

The method validation referred to the International Council for Harmonisation of Technical Requirements for Pharmaceuticals for Human Use (ICH) M10 guideline and related research, including assessments of recovery, matrix effects, and sensitivity to primarily evaluate sample preparation, as well as accuracy, precision, trueness, selectivity, carry-over, freeze–thaw stability, extract stability, long-term plasma stability, and long-sequence robustness to validate the final workflow.^22^

Recovery and matrix effects were determined using QC samples at low (QC_L, 400 pg/mL), mid (QC_M, 1600 pg/mL), and high (QC_H, 10000 pg/mL) levels, using three biological replicates each. The recovery was calculated as defined by Matuszewski et al.:

$$Recovery\;(\%)=\frac{A_{pre‐spike}}{A_{post‐spike}}\times 100$$

and matrix effect as

$$Matrix\;Effect\;\left(\%\right)=\frac{A_{post‐spike}}{A_{direct}}\times 100$$

where A_pre‑spike_ corresponds to the target peak area in QC samples spiked with STD before sample preparation, A_post‑spike_ to the target peak area in extracted blank samples spiked with STD after sample preparation, and A_direct_ to the target peak area in reconstitution solvent spiked with the STD.^23^

The sensitivity was evaluated using calibrators at 1, 5, 10, 20, 50, 100, 200, 500, 2000, 5000, and 25000 pg/mL, each analyzed in triplicate. The limit of quantification (LOQ) was defined as the lowest calibrator concentration for which the mean absolute relative error (ARE) of the back-calculated concentrations relative to the nominal concentration and the coefficient of variation (CV) across triplicate calibrators were below 20%. As an additional acceptance criterion, the coefficient of determination (R^2^) for each calibration curve was required to exceed 0.99. The limit of detection (LOD) was defined as the lowest concentration showing a reasonable peak shape and a signal-to-noise ratio greater than 3 in all replicates.

The accuracy and precision were determined using QC_L, QC_M, and QC_H samples, each analyzed in triplicate. The accuracy was expressed as ARE:

$$Accuracy\;\left(\%\right)=\left|\frac{C_{measured}-C_{nominal}}{C_{nominal}}\right|\times 100$$

Intraday and interday precision were assessed as CVs of replicate analyses within a single day and across three consecutive days, respectively. The acceptance criteria were mean ARE and CV below 20% for QC_L, and below 15% for QC_M and QC_H. Trueness was further evaluated by triplicate quantification of P4, T, and F in standard reference material (SRM) 1950 human plasma, reported as AREs calculated using the mean measured concentrations relative to the corresponding assigned values.

The selectivity was evaluated by measuring the blank matrix in three biological replicates, while carry-over was assessed by injecting blank samples after the highest calibrator (25000 pg/mL). The acceptance criteria required interfering/residual peaks to be less than 20% of the corresponding target response at the LOQ and less than 5% of the respective ISTD response.

The freeze–thaw stability was assessed using QC_L and QC_H samples, and a cattle plasma sample collected during pregnancy, to represent low, high, and endogenous target concentrations. Each sample was analyzed in three independently prepared replicates. Aliquots were subjected to three freeze–thaw cycles between −20 °C and room temperature prior to sample preparation. Extract stability was assessed using the corresponding prepared extracts stored at 4 °C in the autosampler and reanalyzed on days 0, 1, 2, and 7. The stability was calculated as

$$Stability\;\left(\%\right)=\frac{{\left(A/I\right)}_{n}}{{\left(A/I\right)}_{0}}\times 100$$

where (A/I)_n_ is the target-to-ISTD peak area ratio after the indicated freeze–thaw cycle or at reanalysis time n, (A/I)_0_ is the ratio at the starting point. A mean stability within 85–115% was deemed acceptable. The long-term plasma stability was additionally assessed in a cattle plasma sample collected during pregnancy. Independent sample aliquots stored at −20 °C were analyzed on days 0, 25, and 48 after immediate sample preparation upon thawing. The long-term plasma stability was summarized as per-target CVs across the three time points.

The long-sequence robustness was evaluated by continuously applying *Injection 2* of the UHPLC-HRMS method across consecutive measurements of three 96-well plates, corresponding to 111 injections per plate and 333 injections in total. Representative ISTDs covering the (−)-ESI and (+)-ESI segments were monitored using non-normalized peak areas. In parallel, representative targets were evaluated in evenly interspersed QC_L, QC_M, and QC_H samples (n = 2 per level per plate) using target-to-ISTD peak area ratios. Plate-specific signal behavior and chromatographic variability were summarized as overall CVs, plate-specific mean values, and RT drift.

### Data Processing

Raw mass spectral data were acquired using Xcalibur 4.3 and processed in Skyline 24.1 for target identification and quantification. Peak detection was performed using accurate mass filtering of high-resolution mass spectra (70000 fwhm) with a mass accuracy tolerance of ±5 ppm. Targets were identified by matching the extracted peaks to the corresponding calibrator measured within the same analytical batch based on accurate mass and retention time (RT). For sulfate steroid conjugates, an RT tolerance of ±0.15 min was applied, whereas an RT tolerance of ±0.05 min was used for all other targets. The peaks were synchronously integrated, and the peak areas were normalized to their assigned ISTD (Table S1). Calibration curves were constructed using linear regression with a weighting factor of 1/x. Reported quantitative results were classified as compound-specific, valley-split, or composite quantifications. For partially overlapping isomeric target pairs, quantification was performed using a valley-split integration approach, in which the boundary was placed at the local minimum between adjacent peaks and applied consistently across all the samples. Target pairs that could not be resolved by RT and accurate mass were quantified as composite concentrations. All integrations were visually reviewed to confirm RT consistency, peak shape, and correct boundary placement. Additional visualizations were generated in R 4.5.1 (R Core Team, Vienna, Austria).

## Results and Discussion

### Optimization of Sample Preparation

Blood plasma is an aqueous matrix rich in proteins and lipids that interfere with steroidome profiling methods. Therefore, sample preparation is essential to eliminate matrix components and enrich analytes prior to UHPLC-HRMS analysis. To enable automated high-throughput sample preparation in 96-well plates while maintaining method sensitivity, 250 μL of plasma was used as the sample volume. Our workflow focused on an expanded, biologically informed panel of 61 analytes relevant to female reproductive physiology, many of which have not been sufficiently characterized in terms of their functional significance. It included core estrogens, progestogens, androgens, and glucocorticoids and their precursors; selected cytochromes P450 (CYPs)-, hydroxysteroid dehydrogenases (HSDs)- and aldo-keto reductase family 1 (AKR1)-derived oxidative/reductive steroid metabolites, several of which are neurosteroids or estrogenic androgens;^24–26^ major sulfate and glucuronide conjugates as potential circulating steroid reservoirs;^27,28^ and PG metabolites related to luteolysis, ovulation and parturition together with structurally related homologues (Figure 1 and Table S1).^29^

Previously, we developed a methyl *tert*-butyl ether (MTBE)-based liquid–liquid extraction (LLE) procedure tailored specifically to glucocorticoids and progestogens.^21^ However, the current broader panel exhibits pronounced diversity in polarity (0 < logP < 5) and ionization state at neutral pH.^30,31^ For instance, steroid sulfates (p*Ka* < 1), steroid glucuronides (p*K*a ≈ 3), and PG metabolites (p*K*a ≈ 4) are negatively charged at neutral pH, whereas most free steroids hardly ionize.^32^ Consequently, although the MTBE-based LLE was suitable for glucocorticoids and estrogens, the relatively low-polarity and hydrophobicity of MTBE (logP = 1.2) limited its applicability to the broader target panel, necessitating a more versatile extraction approach.

In this study, a solid-phase extraction (SPE) sample preparation procedure with amphiphilic compatibility was proposed and systematically optimized (Figure 2a). The performance of the optimized SPE and LLE was compared in terms of recovery, matrix effects, and sensitivity. Initially, three types of commercially available 96-well SPE plates (Oasis MAX, Strata-X Pro, and Oasis HLB) featuring distinct sorbent chemistries were evaluated according to manufacturer-recommended steroid extraction procedures (Table S3a). As illustrated in Figure 2b, the Oasis MAX plates exhibited poor recovery of steroid conjugates, attributed to irreversible binding between anionic sulfated steroids and quaternary ammonium groups on the sorbent. In contrast, both Strata-X Pro and Oasis HLB plates, which employ amphiphilic polymeric sorbents, demonstrated superior performance across all target categories. Among them, the Oasis HLB plates were ultimately selected for procedure development due to their consistent and robust performance across the entire target panel.

**Figure 2:**
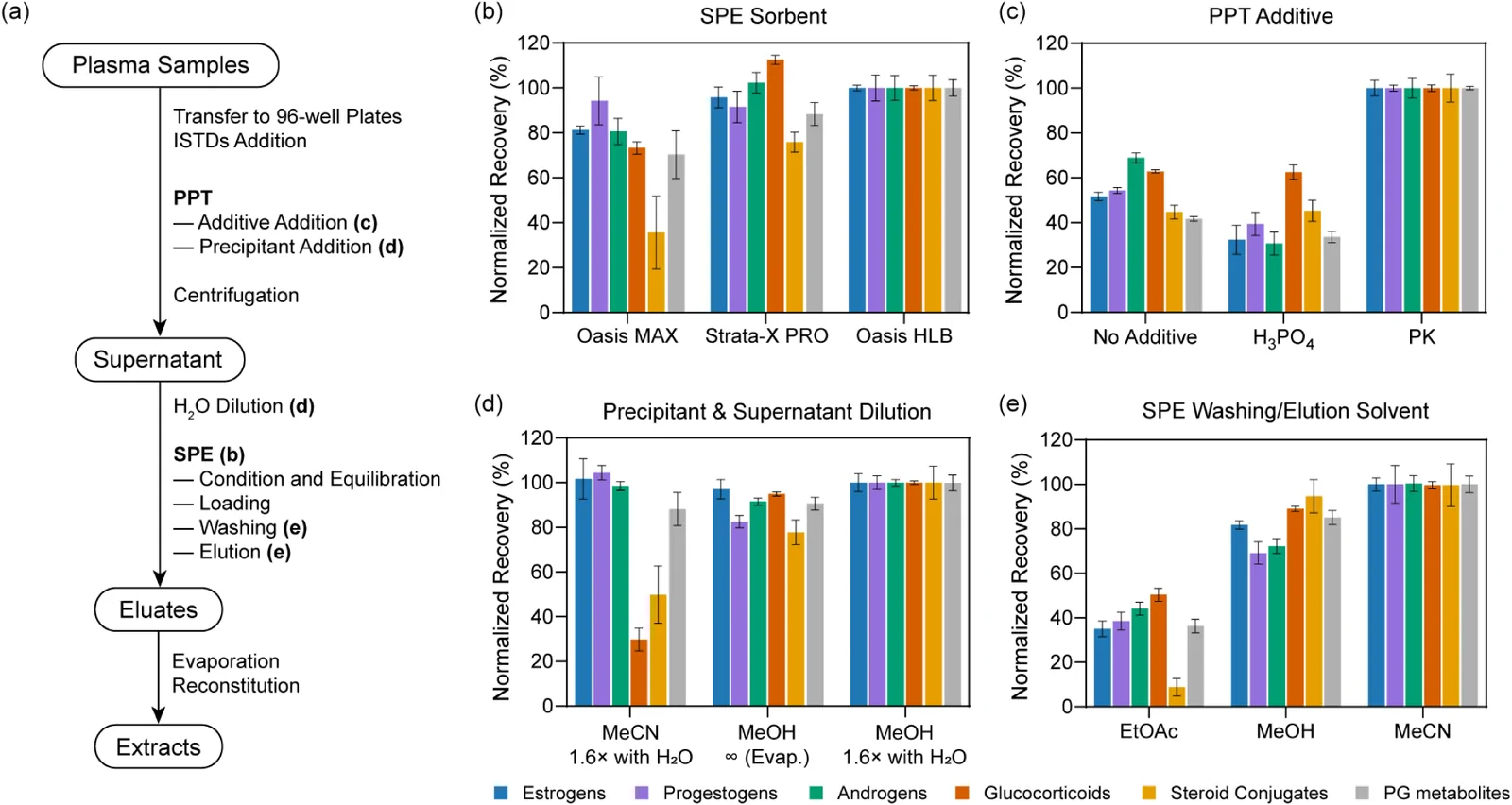
Optimization of the SPE sample preparation procedure. (a) Schematic of the streamlined procedure, including PPT and SPE. Normalized recovery during SPE optimization under different conditions: (b) SPE sorbents, (c) PPT additives, (d) PPT solvents and supernatant dilution factors (∞ indicates complete evaporation of the organic solvent in the supernatant, equivalent to an infinite dilution), and (e) SPE washing/elution solvents. Data are grouped by target category and presented as mean ± standard error of the mean (SEM).

Next, we found that SPE alone was insufficient for the efficient removal of plasma protein, as indicated by a marked increase in system backpressure and a decline in chromatographic column efficiency over prolonged runs. To mitigate this limitation, a protein precipitation (PPT) step was introduced before SPE, as recommended by previous studies.^15^ Two PPT additives were tested to dissociate metabolite–protein binding and enhance the extraction yield of the targets (Table S3b). As illustrated in Figure 2c, the addition of H_3_PO_4_ resulted in reduced recovery, while proteinase K (PK) supplementation significantly increased recovery. This aligns with previous reports showing that PK digestion releases metabolites trapped in precipitated proteins, thereby improving extraction efficiency.^33^ Further optimization of PPT solvents and the pre-SPE dilution step revealed that using MeOH-based PPT followed by a 1.6-fold dilution of the supernatant with H_2_O favored target retention on the SPE sorbent, and provided the highest recovery among the tested conditions (Table S3c and Figure 2d). The final SPE optimization focused on the composition of the washing/elution solvent (Table S3d). MeCN demonstrated improved recovery, which was attributed to its enhanced retention of the targets during the washing step (5% aqueous MeCN) compared to ethyl acetate (EtOAc), and superior elution efficiency (100% MeCN) compared to MeOH, consistent with their polarity differences (Figure 2e).

The optimized SPE procedure was then comprehensively compared to our published LLE method (Figure S1).^21^ As shown in Figure 3a, Tables S4 and S5, the optimized SPE resulted in favorable mean recoveries across all target categories (76–106%). In contrast, LLE yielded lower recoveries for free steroids (67%–71%) and failed to extract steroid conjugates (0%) and PG metabolites (9%). This inefficiency was confirmed by subjecting the residual aqueous phase from LLE to SPE (SPE following LLE, Figure S1), which restored recoveries to 75% for steroid conjugates and 84% for PG metabolites. As depicted in Figure 3b, Tables S4 and S5, satisfactory matrix effects were observed for all targets using the optimized SPE procedure. Free steroids showed mean matrix effects of 83–99%, whereas steroid conjugates were most affected by ion suppression (74%) and PG metabolites displayed slight ion enhancement (110%). For LLE, the matrix effects were acceptable for free steroids (84–118%), but values for steroid conjugates and PG metabolites could not be determined due to their poor recovery.

**Figure 3:**
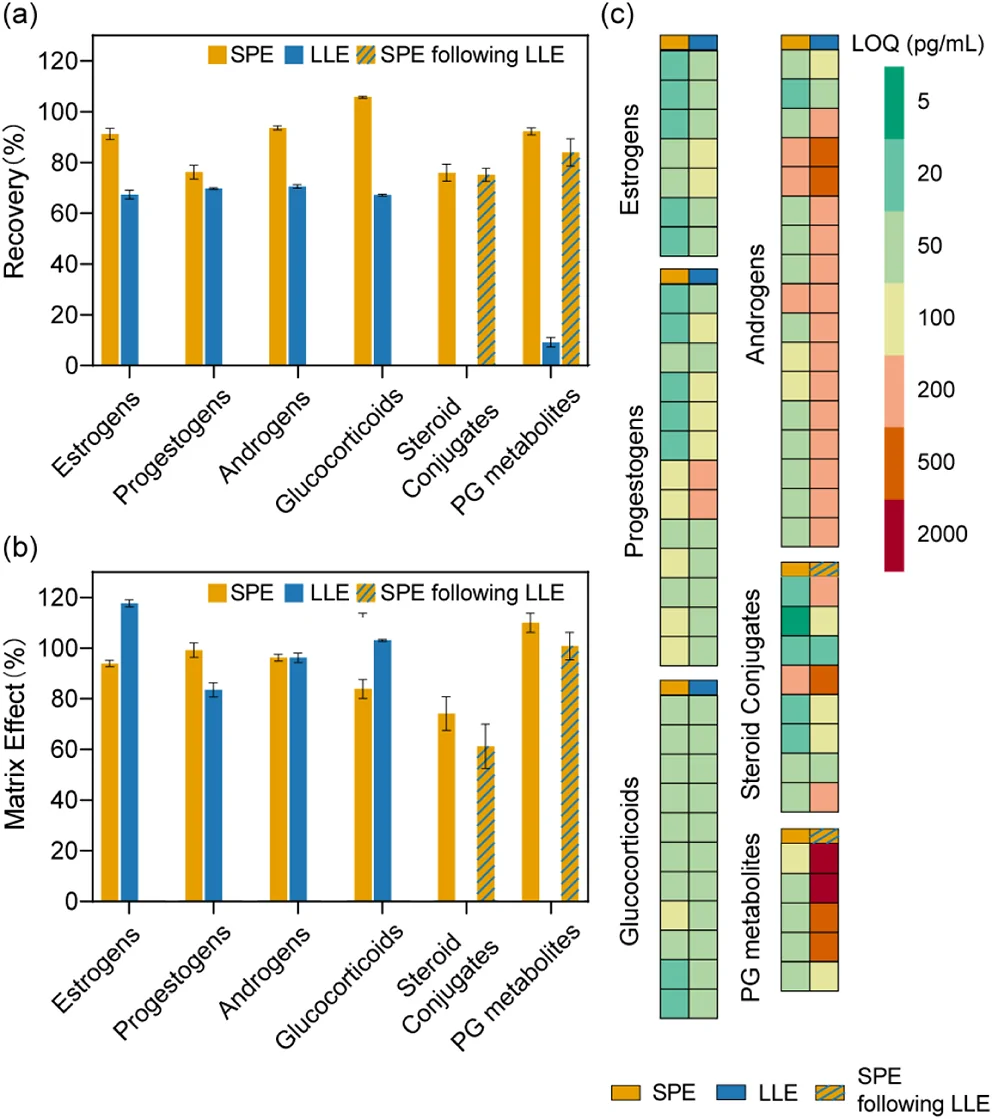
Comparison of sample preparation procedures. (a) Recovery and (b) matrix effects obtained with SPE, LLE, and SPE following LLE. Data are grouped by target category and presented as mean ± SEM. (c) LOQs obtained using different sample preparation procedures, color-coded according to concentration levels.

The sensitivity was compared between the two sample preparation procedures based on LOQs (Figure 3c, Tables S4 and S5). The optimized SPE demonstrated substantially lower LOQs than LLE and SPE following LLE for estrogens (20–50 vs 50–100 pg/mL), androgens (20–200 vs 50–500 pg/mL), steroid conjugates (5–200 vs 20–500 pg/mL), and PG metabolites (50–100 vs 100–2000 pg/mL). In contrast, the LOQs following SPE and LLE, respectively, were comparable for progestogens (20–100 vs 50–200 pg/mL) and glucocorticoids (20–100 vs 50 pg/mL). Since authentic plasma is not devoid of endogenous steroids, the LOQs were established using charcoal-stripped plasma as the best possible surrogate matrix. Admittedly, the actual LOQs in authentic samples may vary depending on endogenous background and analyte-specific matrix effects, particularly at trace concentrations. Alternative calibration strategies, including background subtraction, standard addition, and surrogate analytes, were therefore considered, but proved unfeasible because of the breadth of the panel of multiple targets.^19,34^

In summary, the optimized SPE enabled a broader extraction coverage of plasma steroidome and PG metabolites, resulting in higher recovery, reduced matrix effects, and improved sensitivity compared with LLE. Further comparison with state-of-the-art methods confirmed its superior analytical performance (Table S6).

### Optimization of the UHPLC-HRMS Conditions

Various stationary and mobile phases were thoroughly evaluated to achieve satisfactory target separation. ACQUITY columns with BEH C18, BEH C8, CSH C18, BEH C18 AX, and HSS T3 particle technologies were tested, along with H_2_O, MeOH, and MeCN eluents. Thereby, the best resolution was obtained using the HSS T3 column (2.1 × 100 mm, 1.8 μm) with the mobile phases (A) H_2_O + 0.1% HCOOH and (B) MeCN + 0.1% HCOOH, which is consistent with previous studies.^21^ Full-scan (FS) mode was chosen for mass spectrometric detection over parallel reaction monitoring (PRM) and targeted single ion monitoring (t-SIM). When analytes of different masses coelute, multiplexing is required for PRM and t-SIM but not for FS mode. Thus, in the FS mode, the injection time (247 ms) and mass resolution (70000) could be maximized to ensure excellent sensitivity and mass selectivity.^21,35^

Next, the UHPLC-HRMS method was optimized regarding the ionization efficiency of the target analytes. To achieve full coverage of all target steroids and PG metabolites, the optimization was initiated by evaluating an appropriate ionization source. A heated ESI and an atmospheric pressure chemical ionization (APCI) source were tested using the H_2_O-MeCN eluent system with 0.1% HCOOH. Due to the unfavorable ionization of steroid conjugates with APCI, heated ESI was selected as the ionization method (Figure S2). The ions detected in the (+)-ESI mode were [M + H]^+^, [M – H_2_O + H]^+^ and [M – 2H_2_O + H]^+^, while in the (−)-ESI mode [M – H]^−^ and [M + HCOO]^−^ were observed. The ionization of estrogens was challenging in the (+)-ESI mode due to their low proton affinity.^36^ Furthermore, no deprotonated estrogens (p*K*a = 10.7 for E2) were observed using the acidic mobile phase conditions in the (−)-ESI mode, prompting the evaluation of alternative eluent additives.^37^ To optimize the ionization efficiency for all 61 targets and address the challenges associated with estrogens, three additives including HCOOH, NH_4_F, and NH_4_OH were evaluated. For this comparison, a mixed STD solution (150 pg on-column per target) was injected onto an ACQUITY Premier BEH C18 (2.1 × 100 mm, 1.7 μm) column. As depicted in Figure 4a, progestogens, androgens, glucocorticoids, steroid conjugates, and PG metabolites were readily ionized using HCOOH. Moreover, NH_4_OH and NH_4_F improved estrogen ionization in the (−)-ESI mode but were detrimental to the separation and ionization of steroid conjugates and PG metabolites (Figure S3). Because estrogen levels are typically in the low pg/mL (0–25 pg/mL) range particularly in bovine plasma, maximal sensitivity of the analytical method was required.^38^ Consequently, a dedicated UHPLC-HRMS method was developed targeting estrogens, with NH_4_OH as the additive of choice owing to its superior sensitivity and signal stability compared to NH_4_F.^39,40^

**Figure 4:**
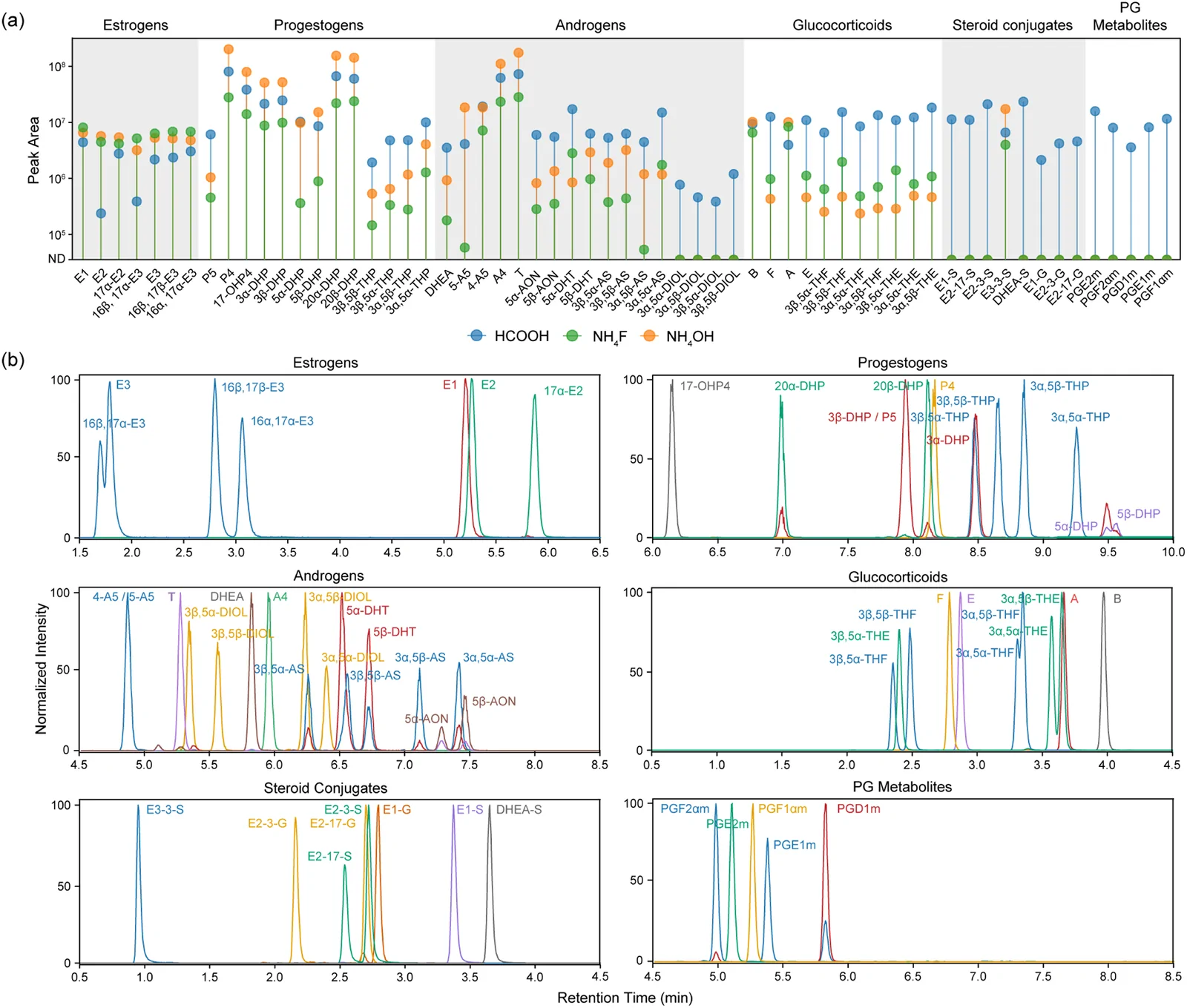
Optimization of UHPLC-HRMS conditions for efficient separation and ionization. (a) Mean peak areas of all targets (150 pg on-column) obtained using 0.1% HCOOH, 0.2 mM NH4F, or 0.1% NH4OH as eluent additives (*n* = 3), determined from their most abundant ions. ND, not detected. (b) Overlaid high-resolution extracted ion chromatograms (±5 ppm) of all targets (150 pg on-column) grouped by category and measured with the two-injection strategy.

Each analyte was mapped to a deuterated ISTD based on their structural similarities (Tables S1 and S2). Position C17 of the estrogen skeleton, which is either an alcohol (E2) or ketone group (E1), was decisive for the assignment of the estrogen analytes to their ISTDs (E2-d_5_ or E1-d_4_). Therefore, the two estradiol stereoisomers (E2 and 17α-E2) and four estriol stereoisomers (E3, 16α,17α-E3, 16β,17α-E3, and 16β,17β-E3) were assigned to E2-d_5_, while estrone (E1) was assigned to E1-d_4_. Furthermore, structurally similar ISTDs were selected for progestogens (P5-d_4_, P4-d_9_, or 17-OHP4-d_8_), androgens (A4-d_7_, T-*d*_3_, or AS-*d*_2_), glucocorticoids (F-*d*_4_), steroid sulfates (E2–3-S-*d*_4_), steroid glucuronides (E2–3-G-*d*_3_), and PG metabolites (PGF2αm-d_4_ or PGE1m-d_4_).

In the final method, all calibrators, QC, and authentic samples were analyzed using a two-injection strategy. *Injection 1* targeted 7 estrogens in the (−)-ESI mode using the BEH C18 column, which is stable under alkaline conditions (0.1% NH_4_OH). *Injection 2* covered the remaining 54 targets, including progestogens, androgens, glucocorticoids, steroid conjugates, and PG metabolites employing an acidic mobile phase (0.1% HCOOH) on the HSS T3 column. Due to additional sulfate, glucuronide, and hydroxy groups, steroid conjugates and glucocorticoids are more polar than androgens or progestogens,^31,41^ resulting in an elution order favorable for polarity switching. All steroid conjugates and glucocorticoids eluted first and were recorded in the (−)-ESI mode. At 4.5 min, the polarity was switched to (+)-ESI for the remaining analytes (Figure S4).

Figure 4b illustrates the chromatographic separation achieved with the two-injection strategy. Remarkably, despite the extensive coverage of 61 analytes, the total runtime remained short (13 min for *Injection 1* and 16 min for *Injection 2*, 0.48 min per target). Thus, the throughput was comparable to or even exceeded reported methods with smaller panels (Table S6). For a limited number of partially overlapping isomeric pairs, including 16β,17α-E3 and E3 (1.70 and 1.79 min), 3α,5α-THF and 3α,5β-THF (3.31 and 3.35 min), and 5α-DHP and 5β-DHP (9.49 and 9.57 min), individual identities were assigned based on RT, and separate quantification was performed by valley-split integration (Figure S5). Because the corresponding peak-to-valley ratios (p/v) did not consistently meet the commonly used criterion of 1.5, these results were classified as valley-split rather than compound-specific quantification. Although these targets were retained as separate entries based on their individual retention times and calibration, reciprocal chromatographic interference cannot be excluded and may affect compound-specific accuracy. Three pairs of isobaric targets could not be resolved by RT and were quantified as sums. The separation of 8 isobaric androgens (C_19_H_30_O_2_) posed the greatest challenge. For example, the four androsterone stereoisomers (3α,5α-AS, 3α,5β-AS, 3β,5α-AS, and 3β,5β-AS) were baseline separated (6.26–7.42 min), however, the isobaric 5α-DHT interfered with 3β,5β-AS at 6.56 min. Furthermore, the regioisomers 4-A5 and 5-A5 differing only by the position of a double bond at C4 and C5 coeluted at 4.87 min. The same issue was observed for the progestogens P5 and 3β-DHP coeluting at 7.94 min. Tandem mass spectrometry (MS/MS) spectra were acquired for these coeluting isobaric steroids, but no unique fragment ions could be identified (Figure S6). For instance, neutral H_2_O losses or a fragment ion at *m*/*z* 81.070 were deemed nonspecific. Therefore, the MS/MS data did not enhance the selectivity for these unresolved steroid pairs and the peak areas represent composite signals rather than compound-specific concentrations. In summary, 49 baseline-separated analytes were quantified individually, three pairs of partially overlapping analytes were quantified by valley-split integration, and three pairs of coeluting analytes were reported as composite concentrations.

### Method Validation

After optimizing the SPE-based sample preparation and the two-injection UHPLC-HRMS conditions, the combined workflow was validated for accuracy, precision, trueness, selectivity, carry-over, freeze–thaw stability, extract stability, long-term plasma stability and long-sequence robustness. As illustrated in Figure 5a–5c and Table S4, the workflow demonstrated robust quantification performance. Mean AREs were 1–16% (QC_L), 2–12% (QC_M), and 3–13% (QC_H). Intraday CVs were 1–19% (QC_L), 1–13% (QC_M), and 1–14% (QC_H), and interday CVs were 1–16% (QC_L), 2–15% (QC_M), and 1–11% (QC_H). All results met the acceptable accuracy and precision criteria. In addition, no group-wise deterioration in accuracy or precision indicative of normalization failure was observed for targets sharing the same assigned ISTD across QC levels. A 1:1 dilution experiment was further performed using an authentic pregnancy cattle plasma sample diluted with charcoal-stripped plasma. For all reliably quantified analytes whose concentrations were above their respective LOQs, dilution-corrected back-calculated concentrations were within 2–15% mean ARE relative to the original sample. These results support that, based on the surrogate matrix calibration and current ISTD assignment strategy, no pronounced systematic bias was evident (Table S7). Furthermore, quantification of SRM 1950 human plasma yielded AREs of 3.4, 5.4 and 10.8% for P4, F, and T, respectively, demonstrating the trueness of the workflow for these representative endogenous steroids (Table S8).

**Figure 5:**
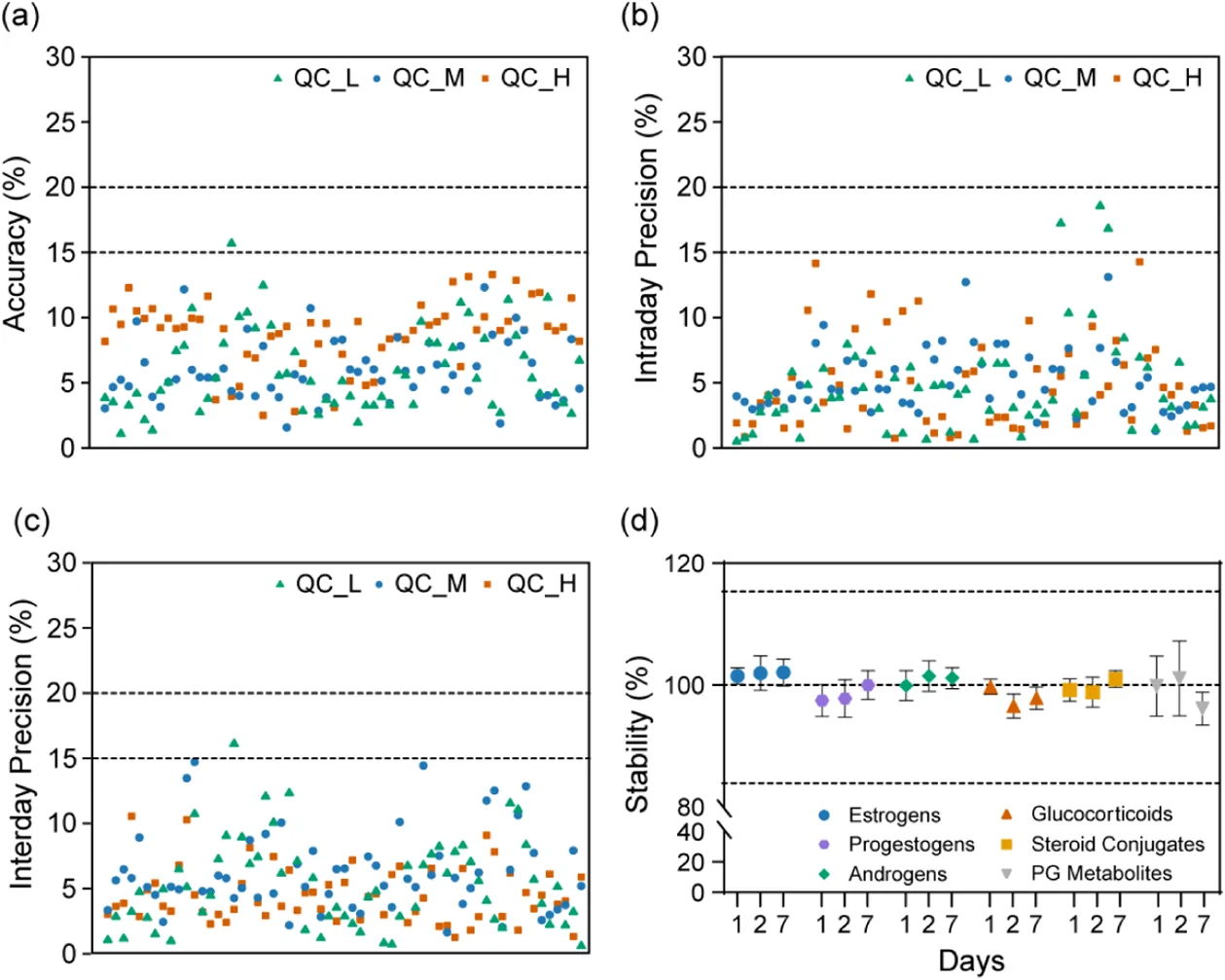
Method validation of the workflow using the optimized SPE and the two-injection UHPLC-HRMS strategy. (a) Mean accuracy, (b) intraday precision, and (c) interday precision of all targets at three QC levels. (d) Extract stability in the autosampler on days 1, 2, and 7. Data are shown as mean ± SEM, grouped by target category.

Interfering peaks were not observed in blank plasma samples, confirming the selectivity of the workflow. Likewise, no significant carry-over was observed after the injection of the highest calibrator, confirming complete analyte elution with the applied chromatographic gradients. Furthermore, target analytes in QC_L and QC_H samples and the cattle plasma sample collected ante-partum remained stable after three freeze–thaw cycles, with mean stability between 96% and 102% across target categories (Figure S7). The corresponding prepared extracts also remained stable in the autosampler at 4 °C for 7 days, with mean stability between 85% and 104% across target categories (Figure 5d). In addition, long-term storage of a plasma sample collected from pregnant cattle (storage at −20 °C) resulted in minimal variation across reanalysis on days 0, 25, and 48, with CVs per analyte ranging from 1% to 16% (Figure S8).

The long-sequence robustness was evaluated by measuring three 96-well plates consecutively using *Injection 2* of the UHPLC-HRMS method, which corresponded to a total of 333 injections. Representative ISTDs monitored at the raw response level showed plate-dependent variation, especially in the (−)-ESI segment with overall CVs of 23–26%, while the (+)-ESI segment had overall CVs of 10–14% (Table S9). In contrast, representative targets monitored in evenly interspersed QC samples showed stable normalized responses, with overall CVs of 2–10% (Table S10). The within-plate/batch RT drift of most targets was minimal (0–0.02 min), whereas only sulfate steroid conjugates showed larger variability (0–0.13 min), which was compatible with the RT matching criteria for target identification. Maximum system backpressure showed no progressive increase across the sequence (Figure S10). Excluding conditioning and column cleaning runs, the pressure span was 24.8 bar with a slight downward trend, providing no pressure-based evidence of progressive phospholipid buildup in the column. These results support the robustness and reliability of the workflow in large-scale studies.

### Steroidome and PG Metabolite Profiling in Authentic Plasma Samples

As a final step, the validated workflow was applied to authentic bovine and human plasma samples to assess its performance in real biological matrices within distinct physiological contexts. Unlike humans, rodent animal models are characterized by short polyovulatory estrous cycles and comparatively altricial offspring. Cattle as large animal model, on the other hand, exhibit striking similarities to humans, particularly regarding ovarian function, embryonic and fetal development, and reproductive senescence. These similarities underscore the relevance of cattle as a model organism for translational studies in the field of female reproduction.^42,43^ Based on these considerations, we utilized both human and cattle samples for the biological validation of the established method. The selected physiological states were chosen because they span well-defined endocrine conditions with marked differences in endogenous steroid and PG-related profiles, including cyclical fluctuations in ovarian steroids, pregnancy- and birth-related changes in progestogens, estrogens, and PGF2α-related metabolism, as well as androgen-rich male plasma. Accordingly, cattle samples included specimens collected across the estrous cycle, during pregnancy and parturition, and from a bull. Furthermore, human samples from a single adult woman across the menstrual cycle and pregnancy, as well as samples from a single adult male, were analyzed.

Each cattle plasma sample was prepared and analyzed in triplicate to evaluate reproducibility. Across all quantified analytes, CVs ranged from 0–15% above the respective LOQs, with 82% of targets exhibiting CVs below 10%, indicating stable sample preparation and quantification under high-throughput conditions (Figure S9). As shown in Figure 6 and Table S11, a total of 42 targets were quantitatively profiled above their respective LOQs, comprising 38 reported by compound-specific quantification, 2 by valley-split quantification, and 2 represented by a composite concentration. These targets include established endocrine markers together with multiple low-abundance and less-characterized metabolites spanning estrogen, progestogen, androgen, glucocorticoid, steroid conjugate, and PG pathways. The data set demonstrates simultaneous quantification across 5 orders of magnitude at endogenous concentrations. As expected, multiple progestogens were detected in pregnancy and parturition-associated samples, consistent with the reported vast steroidogenic capacity of the placenta before and during cattle parturition.^44,45^ PG metabolites such as PGE2 and PGF2α are known to induce uterine contraction and hemostasis and are rapidly metabolized to PGE2m and PGF2αm, respectively.^18,46^ We simultaneously detected PGE2m and PGF2αm, and their homologues PGE1m and PGF1αm around parturition, demonstrating concurrent coverage of prostaglandin metabolism and steroid pathways in the workflow.

**Figure 6:**
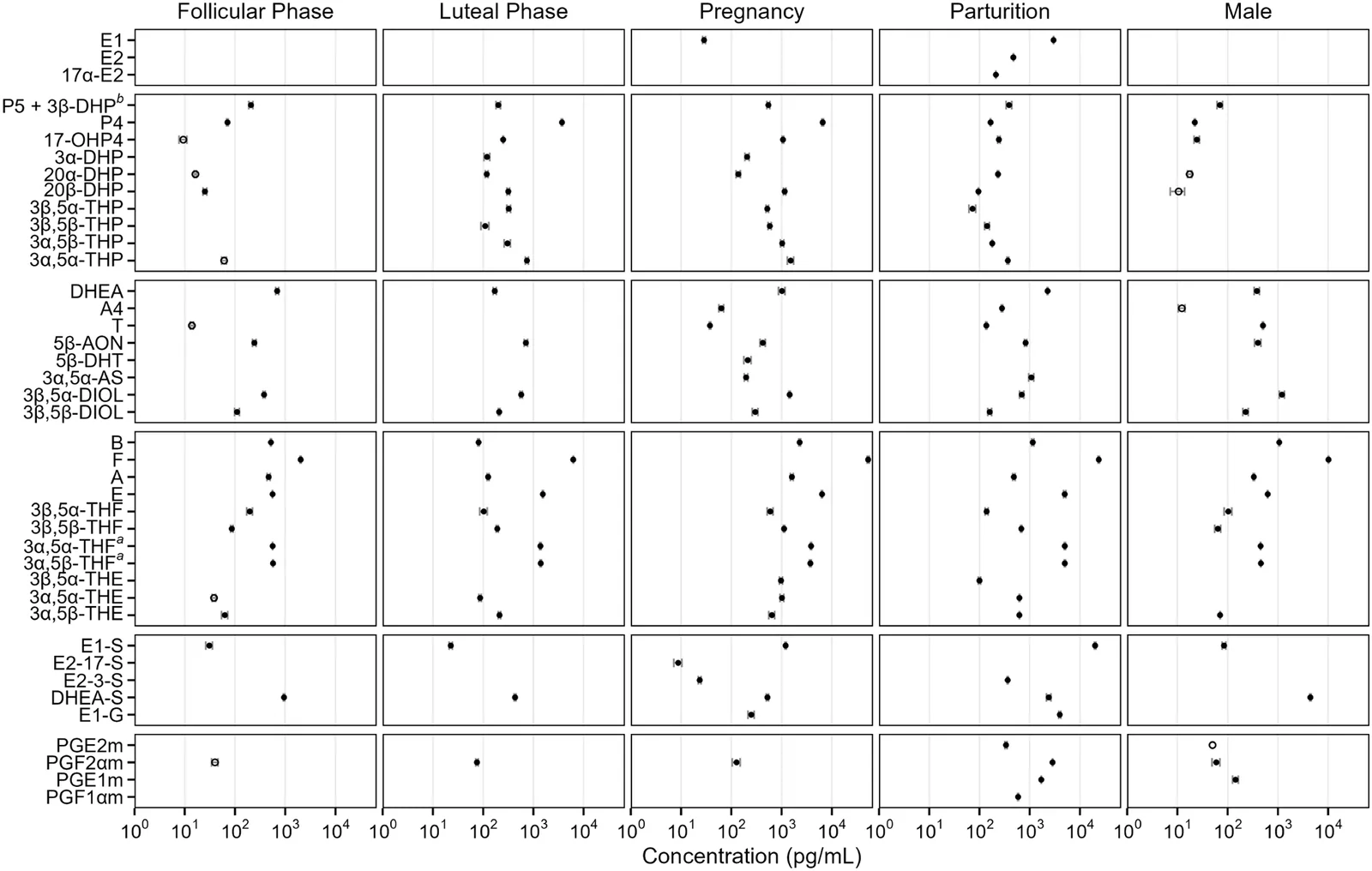
Concentrations of the 42 targets detected in cattle plasma samples, grouped by category and presented as mean ± standard deviation (SD). Hollow points indicate mean levels below the corresponding LOQs. *^a^* Concentrations reported by valley-split quantification. *^b^* Concentrations reported by composite quantification.

In human plasma, a total of 48 targets were quantitatively profiled above their respective LOQs. Of these, 38 were reported by compound-specific quantification, 4 by valley-split quantification, and 6 represented by three composite concentrations (Figure 7 and Table S12). An extensive spectrum of targets was encompassed, including 3 estrogens, 12 progestogens, 14 androgens, 11 glucocorticoids, 8 steroid conjugates, all detected across a wide range of concentrations. To further assess the biological plausibility of these measurements, the observed concentration ranges were compared with available human reference intervals (RIs). Of the 35 assessable comparisons, 22 were within and 13 partially overlapped the corresponding RIs, none fell entirely outside the RI or differed in order of magnitude (Table S13). Qualitative characteristics, including the presence of progestogens in samples taken during the luteal phase and during pregnancy, confirm the suitability of the workflow for detecting the well-established steroidogenesis by the corpus luteum and the placenta.^47,48^

**Figure 7:**
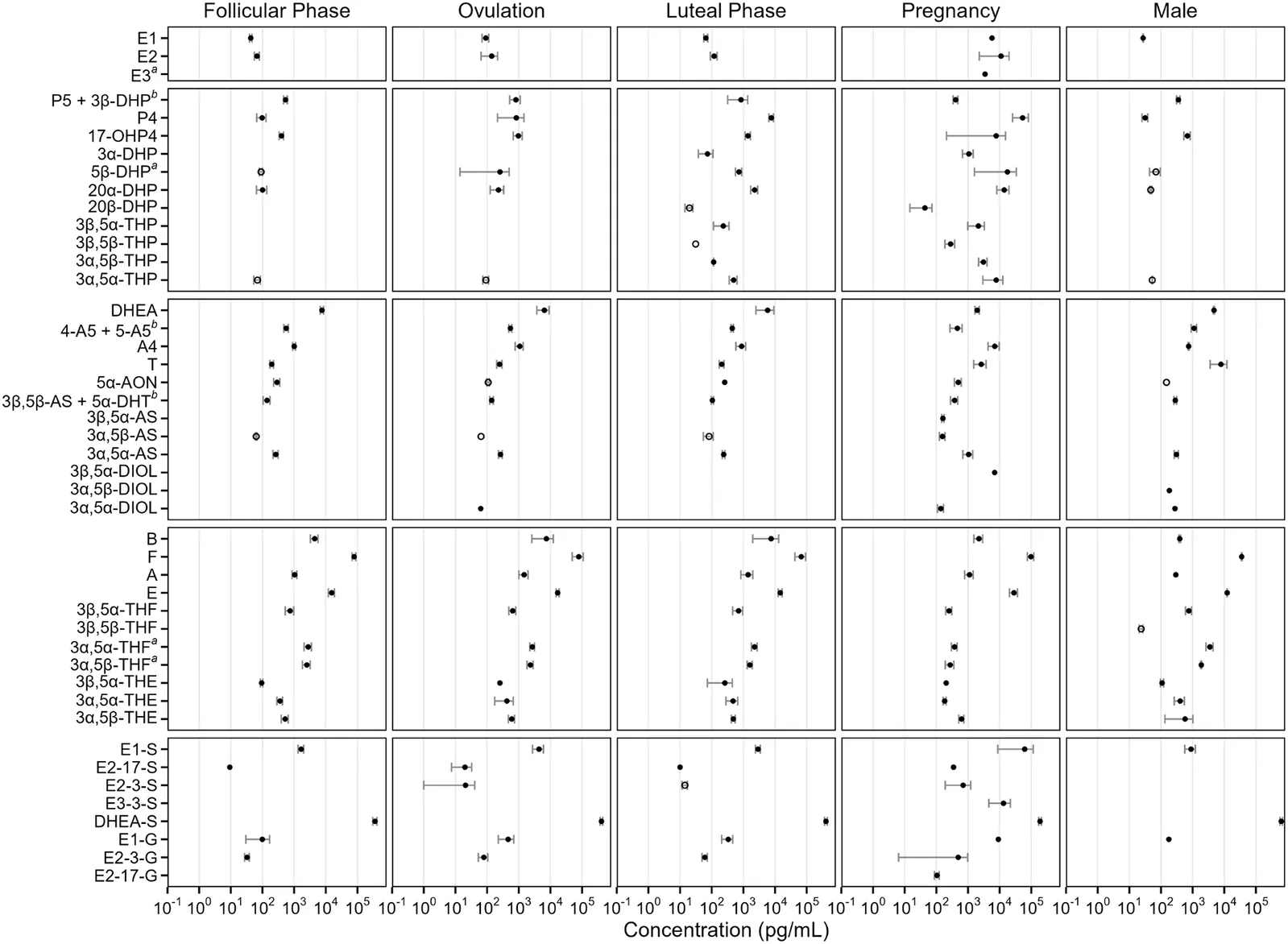
Concentrations of the 48 targets detected in human plasma samples, grouped by category and presented as mean ± SD. Hollow points indicate mean levels below the corresponding LOQs. *^a^* Concentrations are reported by valley-split quantification. *^b^* Concentrations are reported by composite quantification.

Overall, the application of the workflow to cattle and human plasma samples demonstrates its ability to provide robust and broad-panel quantification of steroidome and PG metabolites in authentic biological matrices. This underscores the workflow’s suitability for future translational studies across species. Given the limited number of individuals used in this study, the results represent a proof-of-concept demonstration of analytical performance rather than population-level biological inference. Larger-cohort studies are ongoing to systematically investigate interindividual variability and endocrine dynamics.

## Conclusions

In summary, we developed and validated an analytical workflow for quantitative profiling of 61 selected steroids and PG metabolites in 250 μL of plasma. The workflow included an automated SPE sample preparation with PPT pretreatment, followed by a two-injection UHPLC-ESI-HRMS analysis with runtimes of 13 and 16 min (total runtime of 29 min). Our calibration strategy based on charcoal-stripped plasma as a surrogate matrix resulted in low LOQs of 5–200 pg/mL. The performance and long-sequence robustness were validated through standard validation metrics. Application to authentic cattle and human plasma samples further demonstrated broad target coverage and reproducible quantification in biological matrices across distinct physiological contexts. Owing to the FS HRMS acquisition strategy, the validated target panel is readily expandable as standards of additional related analytes become available and also supports future semitargeted applications.^49^ Together, this workflow provides a foundation for cohort studies to investigate the complex endocrine dynamics of the steroidome and PG metabolism under physiologically and clinically relevant conditions in the context of female reproduction.

## Supplementary Material



## Notes

Chemicals and reagents; preparation of charcoal-stripped plasma as the surrogate matrix; automated sample preparation configuration; LLE procedure; schematic representation of the sample preparation procedures; HRMS signal intensities of the targets using ESI and APCI; overlaid extracted ion chromatograms of PG metabolites with different eluent additives; plot of monitored ions against their retention times for the two chromatographic methods; high-resolution extracted ion chromatograms illustrating the identification and quantification of partially overlapping isomeric target pairs; MS/MS spectra of three pairs of coeluting isobaric steroids; freeze–thaw stability of targets in plasma samples; long-term plasma stability of targets in a plasma sample; CVs of quantified targets in cattle plasma samples derived from triplicate sample preparation and analysis; maximum system backpressure recorded throughout the long-sequence across consecutive measurements of three 96-well plates; summary of target standards; summary of ISTDs; evaluation of SPE sorbents; optimization of PPT additives, PPT solvents, and supernatant dilution factors; optimization of SPE washing/elution solvents; method validation results for each target using SPE, LLE, and SPE following LLE; comparison of the overall performance of the current workflow with state-of-the-art methods; results for the dilution experiment using an authentic pregnancy cattle plasma sample diluted with charcoal-stripped plasma; trueness assessment using SRM 1950 human plasma; long-sequence response of representative ISTDs and targets across consecutive measurements of three 96-well plates; quantified profiles of the steroidome and PG metabolites in cattle and human plasma samples; comparison of target concentrations measured in human plasma samples with available reference intervals (PDF)
