# Supplementary material for: Extensive Quantitative Profiling of Plasma Steroidome and Prostaglandin Metabolites for Studies of Female Reproductive Physiology
Source: Anal Chem. 2026 Sep 2;98(36):26818–30. doi: 10.1021/acs.analchem.6c03626 (PMC13584695; doi:10.1021/acs.analchem.6c03626)
Supplement: Supplementary file 1 [file ac6c03626_si_001.pdf]

SUPPORTING INFORMATION

Extensive quantitative profiling of plasma steroidome and prostaglandin metabolites for studies of female reproductive physiology

Haolin Chen,[1,⊥] *Luca Bürgi,*[2,⊥] *Anna-Katharina Hankele,*[1] *Reto Giacometti,*[1] *Susanne E. Ulbrich,*[1,*] *and Laurent Bigler*[2,*]

[1] ETH Zurich, Animal Physiology, Institute of Agricultural Sciences, Universitaetstrasse 2, 8092 Zurich, Switzerland

[2] University of Zurich, Department of Chemistry, Winterthurerstrasse 190, 8057 Zurich, Switzerland

⊥ These authors contributed to the work equally.

* Corresponding authors

Table of Contents

Chemicals and Reagents S3

Preparation of Charcoal-Stripped Plasma as the Surrogate Matrix S3

Automated Sample Preparation Configuration S4

Liquid-Liquid Extraction Procedure S5

Figure S1. Schematic representation of the sample preparation procedures S6

Figure S2. HRMS signal intensities of the targets using ESI and APCI S7

Figure S3. EICs of PG metabolites with different eluent additives S8

Figure S4. Monitored ions (*m*/*z*) plotted against their retention times S9

Figure S5. EICs of partially overlapping isomeric target pairs S10

Figure S6. MS/MS spectra of three pairs of coeluting steroids S11

Figure S7. Stability of targets in plasma after freeze-thaw cycles S12

Figure S8. Long-term plasma stability of targets in a cattle sample S12

Figure S9. Coefficients of variation of quantified targets in cattle plasma samples S13

Figure S10. Maximum system backpressure S14

Table S1. Summary of target standards S15

Table S2. Summary of internal standards S17

Table S3a. Evaluation of SPE sorbents S18

Table S3b. Optimization of PPT additives S18

Table S3c. Optimization of PPT solvents and supernatant dilution factors S19

Table S3d. Optimization of SPE washing/elution solvents S19

Table S4. Method validation results using SPE S20

Table S5. Method validation results using LLE and SPE S22

Table S6. Comparison of the current workflow with state-of-the-art methods S24

Table S7. Results of cattle plasma sample diluted with charcoal-stripped plasma S25

Table S8. Trueness assessment using SRM 1950 human plasma S27

Table S9. Long-sequence response of ISTDs S28

Table S10. Long-sequence response of representative targets S28

Table S11. Quantitative profiles in cattle plasma samples S29

Table S12. Quantitative profiles in human plasma samples S31

Table S13. Comparison of target concentrations with reference intervals S33

References S35

Chemicals and Reagents

Steroid reference material was purchased from Steraloids (Newport, RI, USA). Prostaglandin (PG) reference material was purchased from Cayman Chemical (Ann Arbor, MI, USA). Sodium hydrogen phosphate dihydrate ($Na_2HPO_4 \cdot 2H_2O$), sodium chloride (NaCl), activated charcoal, ethyl acetate (EtOAc), calcium chloride ($CaCl_2$), zinc sulfate heptahydrate ($ZnSO_4 \cdot 7H_2O$), ammonium fluoride (NH_4F), and proteinase K (PK) lyophilized powder were obtained from Merck KGaA (Darmstadt, Germany). UHPLC-MS grade acetonitrile (MeCN), methanol (MeOH) and formic acid (HCOOH) were purchased from Biosolve BV (Valkenswaard, Netherlands). LC-MS grade eluent additive ammonium hydroxide (NH_4OH, 25%) was purchased from VWR International (Dietikon, Switzerland). HPLC grade methyl tert-butyl ether (MTBE) was purchased from Honeywell (Seelze, Germany). Ultra-pure H_2O (< 5 ppb TOC) was produced with a Milli-Q® Advantage A10 water purification system (Merck, Bedford, MA, USA). Standard reference material (SRM) 1950 human plasma was obtained from the National Institute of Standards and Technology (NIST, Gaithersburg, USA).

Preparation of Charcoal-Stripped Plasma as the Surrogate Matrix

A total of 5 g of activated charcoal was added to 41.7 mL of assay buffer (pH 8.0, containing 7.12 g/L $Na_2HPO_4 \cdot 2H_2O$ and 8.5 g/L NaCl) and stirred at 4 °C for 16 h. The charcoal solution (10 mL) was then mixed with 40 mL of bovine plasma (collected between one and five days post-partum) and incubated at 4 °C for another 16 h. Subsequently, the mixture was centrifuged at 1500 rpm for 10 min to collect the supernatant. An additional 5 mL of charcoal solution was added to the supernatant, followed by incubation at 37 °C for 2 h. After a second centrifugation at 1500 rpm for 10 min, the supernatant was collected, and the incubation with 5 mL charcoal solution and centrifugation was repeated. The final supernatant was filtered using 0.22 µm poly(ether sulfone) syringe filters (Merck KGaA, Darmstadt, Germany) to obtain charcoal-stripped plasma as a surrogate matrix, which was stored at −20 °C until further use.

Automated Sample Preparation Configuration

All liquid transfers were performed using the Andrew+ pipetting robot. An eight-channel pipette with a volume range of 5–120 µL (Sartorius Picus, Tagelswangen, Switzerland) was used to transfer the ISTD working solution, proteinase K, and $CaCl_2$, whereas an eight-channel pipette with a volume range of 50–1200 µL was used for all other larger-volume liquid transfers. Corresponding 5–120 and 50–1200 µL Optifit tips (Sartorius Picus, Tagelswangen, Switzerland) were positioned in the Tip Insertion System Domino modules (No. 218.1101). Deepwell Microplate Domino modules (No. 218.2301) were used to position 96-well plates containing the plasma samples, 96-well plates used for collection and dilution of the supernatants after protein precipitation, and the Oasis HLB 96-well SPE plates. A Deepwell Microplate Domino module was also used to position a four-well horizontal reservoir (Analytical Sales & Services, Inc., Flanders, USA) containing the larger-volume aqueous and organic solutions. The ISTD working solution, proteinase K, and $CaCl_2$ were placed in 10 mL multichannel reservoirs (INTEGRA Biosciences AG, Zizers, Switzerland) positioned in 8-channel Pipette Reservoir Domino modules (No. 218.2182).

Liquid transfers were performed in forward-pipetting mode with blow-out completion. Slow aspiration and dispensing speeds, and normal mechanical arm motion and pipette moving speeds were used. No air cushion was applied for aqueous solutions, whereas an air bottom cushion for low viscosity aspiration was applied to solutions containing organic solvents. During aspiration, the tip was positioned 0.5 mm below the liquid surface, and destination wells were accessed using the on-the-fly setting.

Liquid-Liquid Extraction Procedure

Liquid-liquid extraction (LLE) was conducted in 96-deep-well plates using an automated workflow. Each well was loaded with 250 µL of calibrators, QCs, or authentic plasma samples, followed by 10 µL of the ISTD working solution. The plate was then shaken at 2000 rpm for 5 minutes. Next, 1 mL of methyl tert-butyl ether (MTBE) was added to each well and the well plate was closed with a silicone sealing mat. The plate was shaken at 25 °C and 2000 rpm for 5 min and subsequently rested for 5 min to allow phase separation. Then, 900 µL of the upper organic phase was collected and evaporated under a N_2 stream at 45 °C. The resulting dry extracts were stored at −20 °C until analysis. Prior to UHPLC-HRMS analysis, dried extracts were reconstituted in 100 µL of MeOH/H_2O (2:3, v/v) and shaken at 2000 rpm for 5 min.

Extraction with LLE led to poor recoveries for steroid conjugates and PG metabolites. To confirm this finding, the aqueous phase remaining after LLE was extracted by SPE using the same procedure as described in the Materials and Methods section.

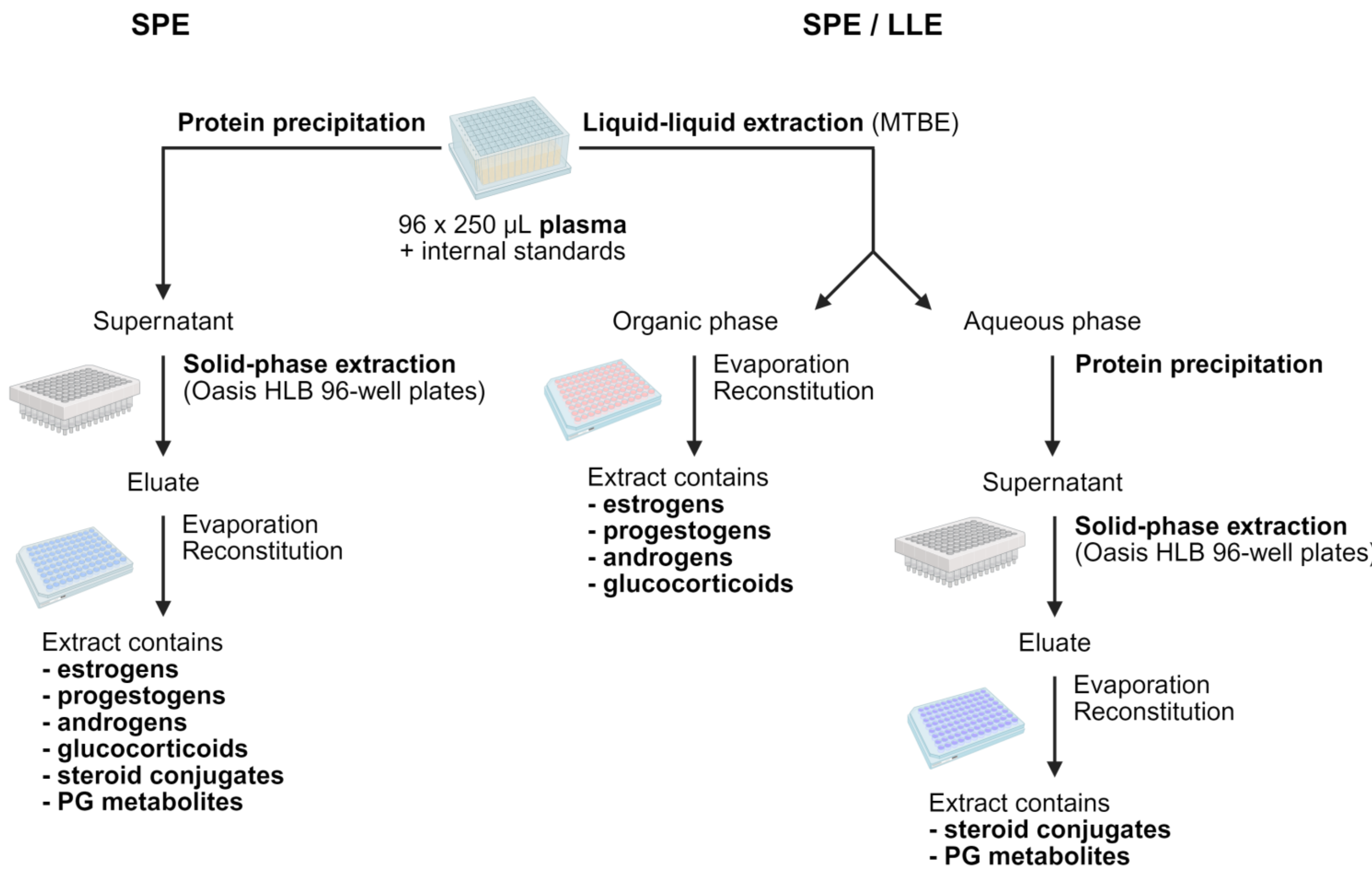

Figure S1. Schematic representation of the sample preparation procedures, including solid-phase extraction (SPE) and liquid-liquid extraction (LLE) supplemented by SPE. The LLE protocol was adopted from our previous study.[1]

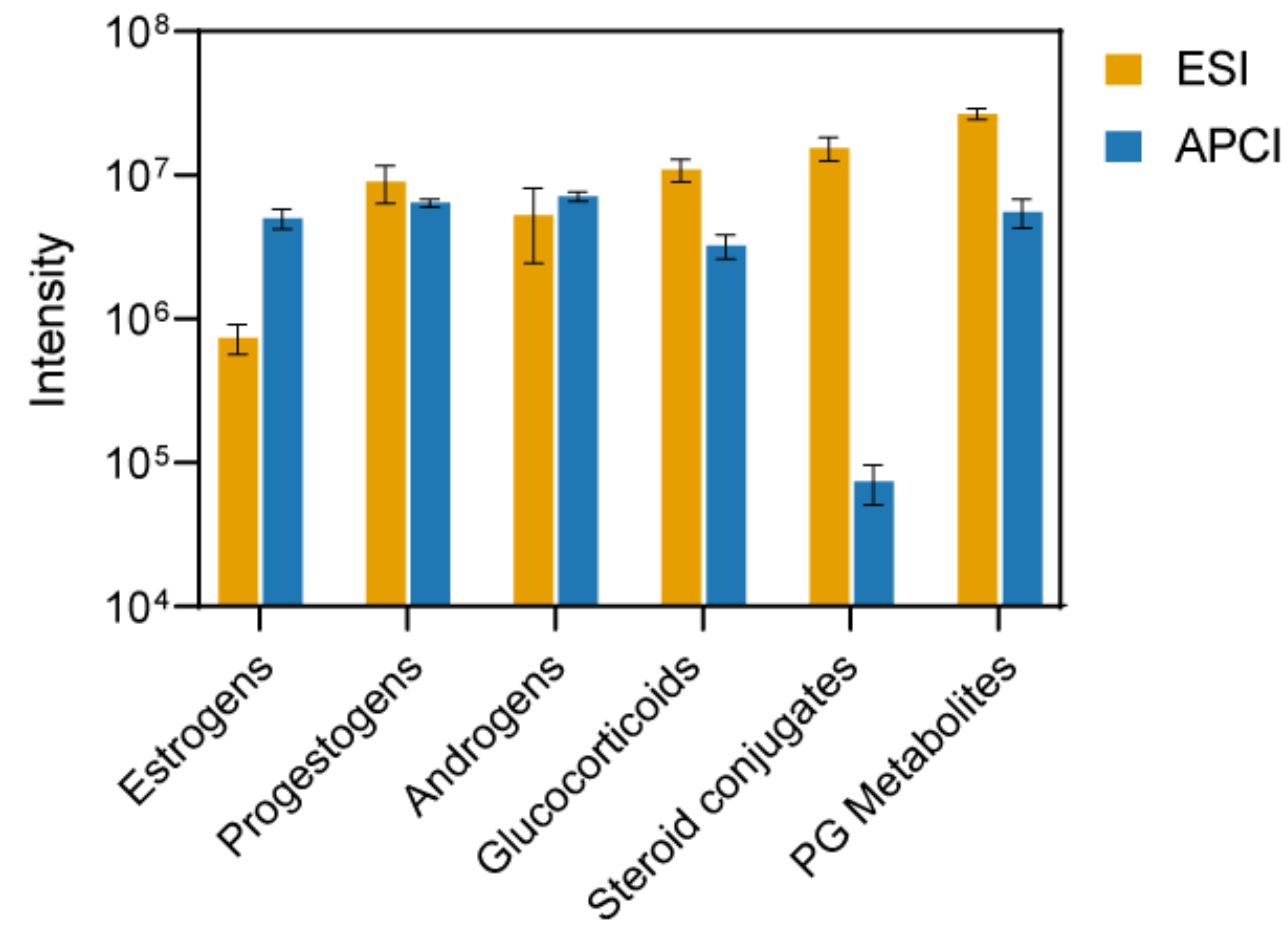

Figure S2. High-resolution mass spectrometry (HRMS) signal intensities of the targets at 5 ng acquired by on-flow injection of individual standards under the H_2O-MeCN eluent system with 0.1% HCOOH, using ESI and APCI. Intensities were calculated based on the most abundant ions. Data are grouped by target category and presented as mean ± standard error of the mean (SEM).

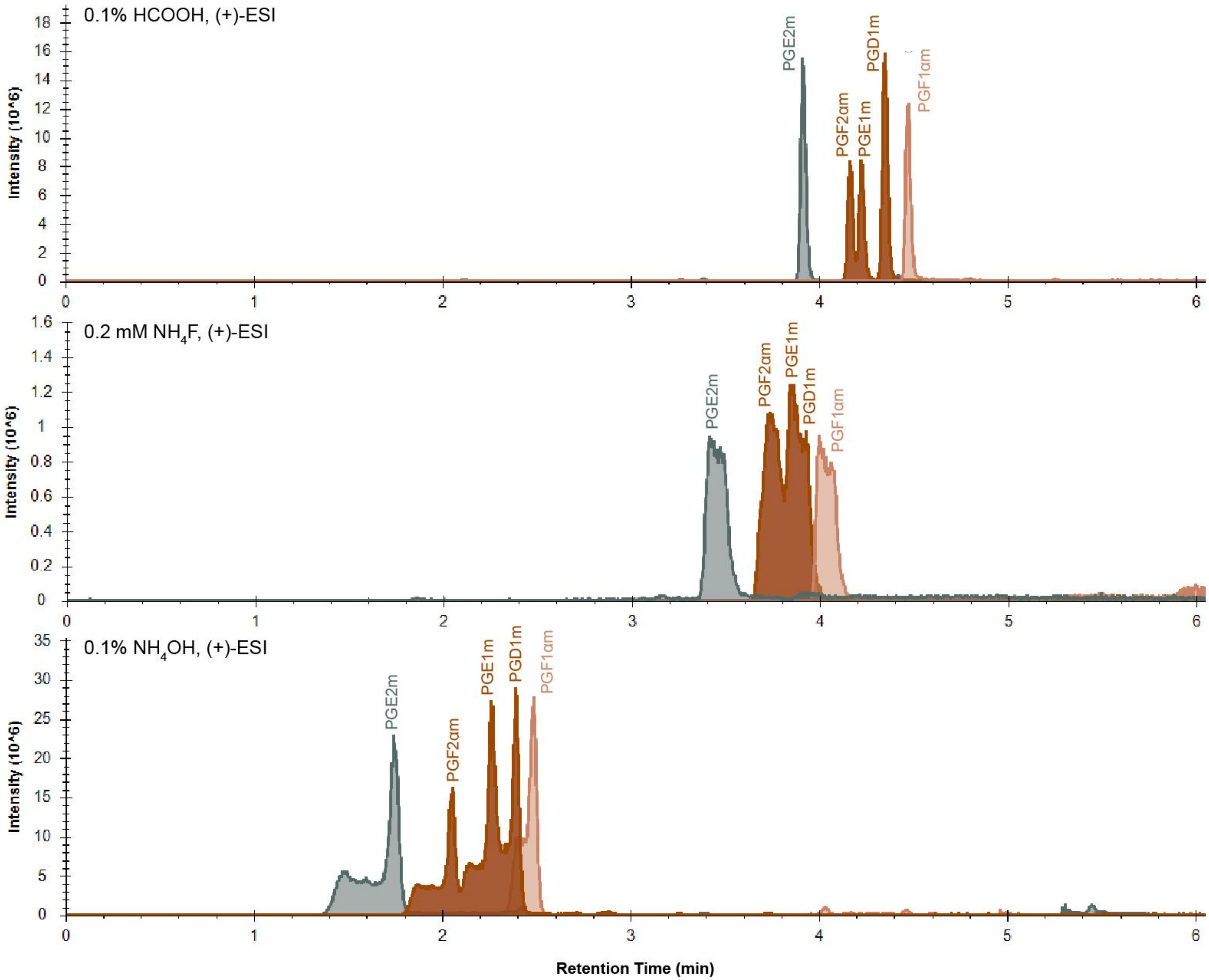

Figure S3. Overlaid extracted ion chromatograms (±5 ppm) of PG metabolites at 10 ng/mL, analyzed by UHPLC-HRMS in the (+)-ESI mode, using a BEH C18 column with different eluent additives, including 0.1% HCOOH, 0.2 mM NH_4F, and 0.1% NH_4OH.

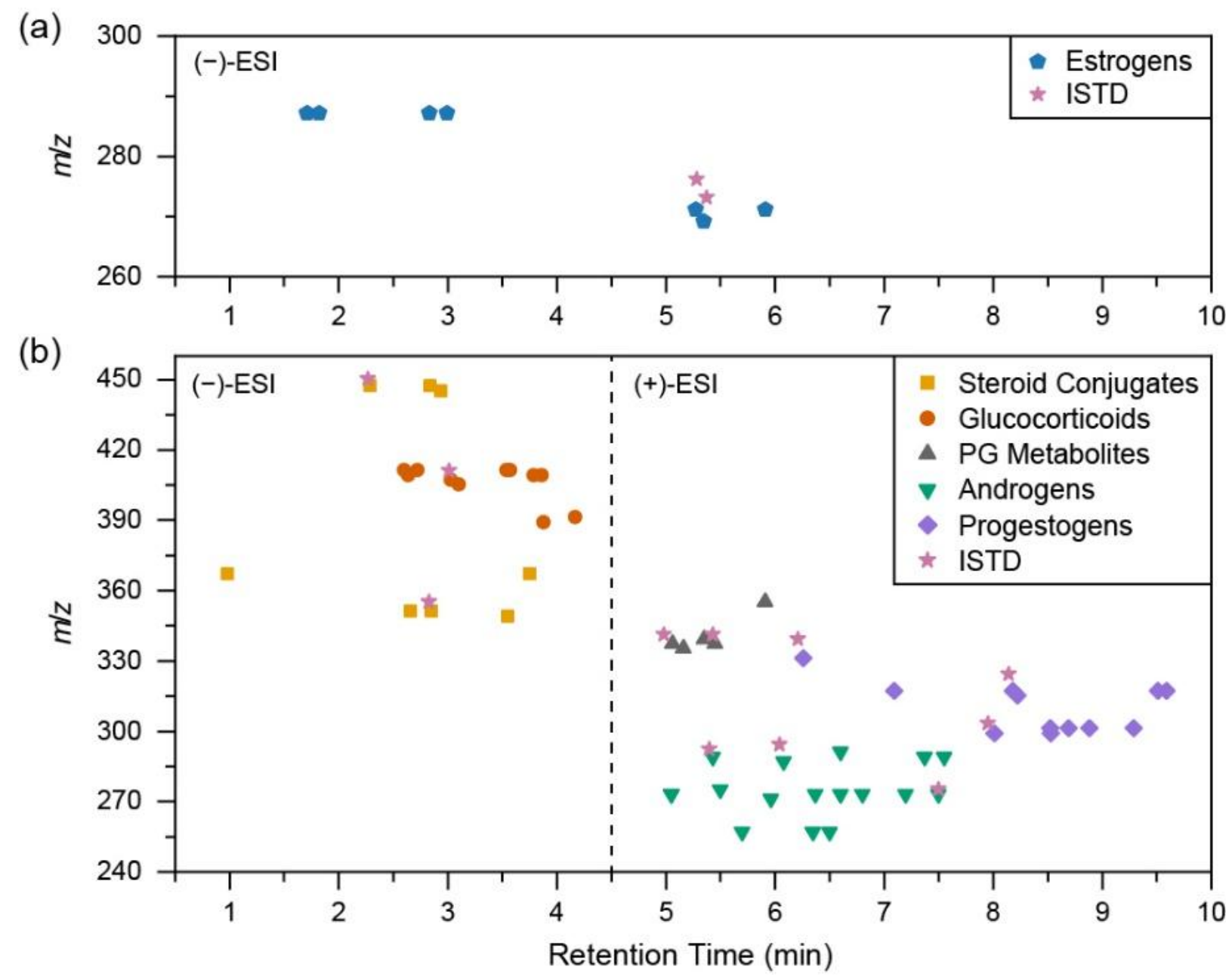

Figure S4. Plot of monitored ions (*m/z*) against their retention times for comparison of the two chromatographic methods: (a) *Injection 1* – Estrogens were separated on a BEH C18 column with 0.1% NH_4OH as additive and ionized in (−)-ESI mode; (b) *Injection 2* – Steroid conjugates, glucocorticoids, PG metabolites, androgens, and progestogens were separated on a HSS T3 column with 0.1% HCOOH as eluent additive and ionized with polarity switch from (−)-ESI to (+)-ESI mode at 4.5 min.

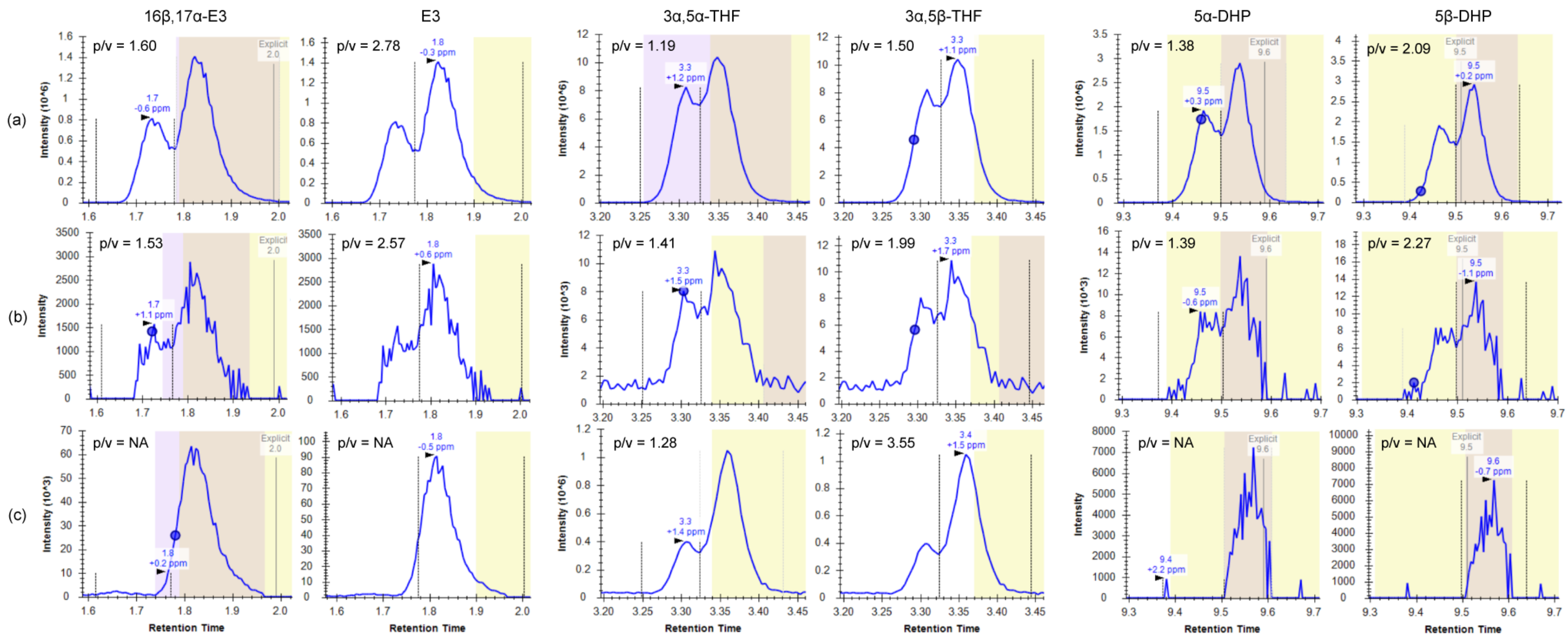

Figure S5. High-resolution extracted ion chromatograms (±5 ppm) illustrating the identification and quantification of partially overlapping isomeric target pairs in (a) the highest calibrator (25000 pg/mL), (b) the calibrators at corresponding LOQ levels, and (c) authentic plasma samples. Target identity was assigned by retention time matching within ± 0.05 min. The black dotted lines indicate the integration boundaries. Peak-to-valley ratios (p/v) are indicated.

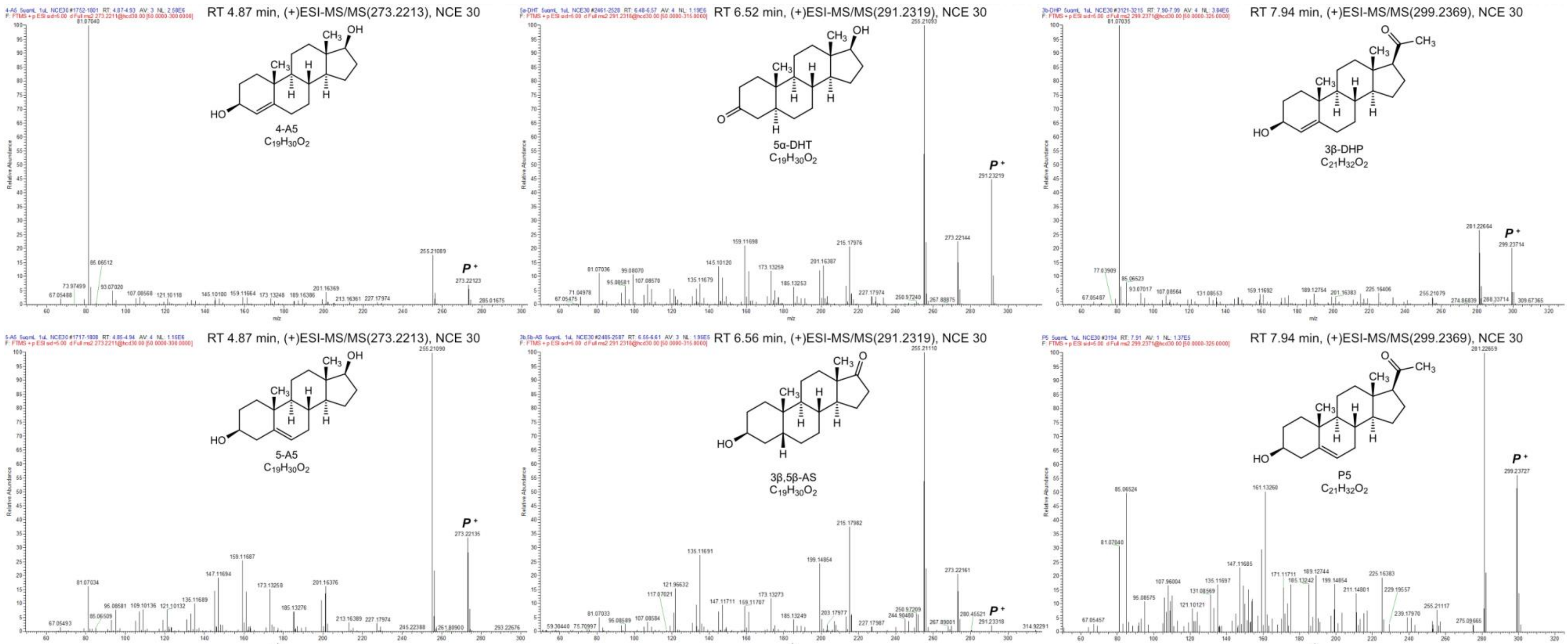

Figure S6. MS/MS spectra of three pairs of coeluting isobaric steroids (RT 4.87 min: 4-A5 and 5-A5; RT 6.52–6.56 min: 5α-DHT and 3β,5β-AS; RT 7.94 min: 3β-DHP and P5). Precursor ions (***P***$^+$) were fragmented at a normalized collision energy (NCE) of 30.

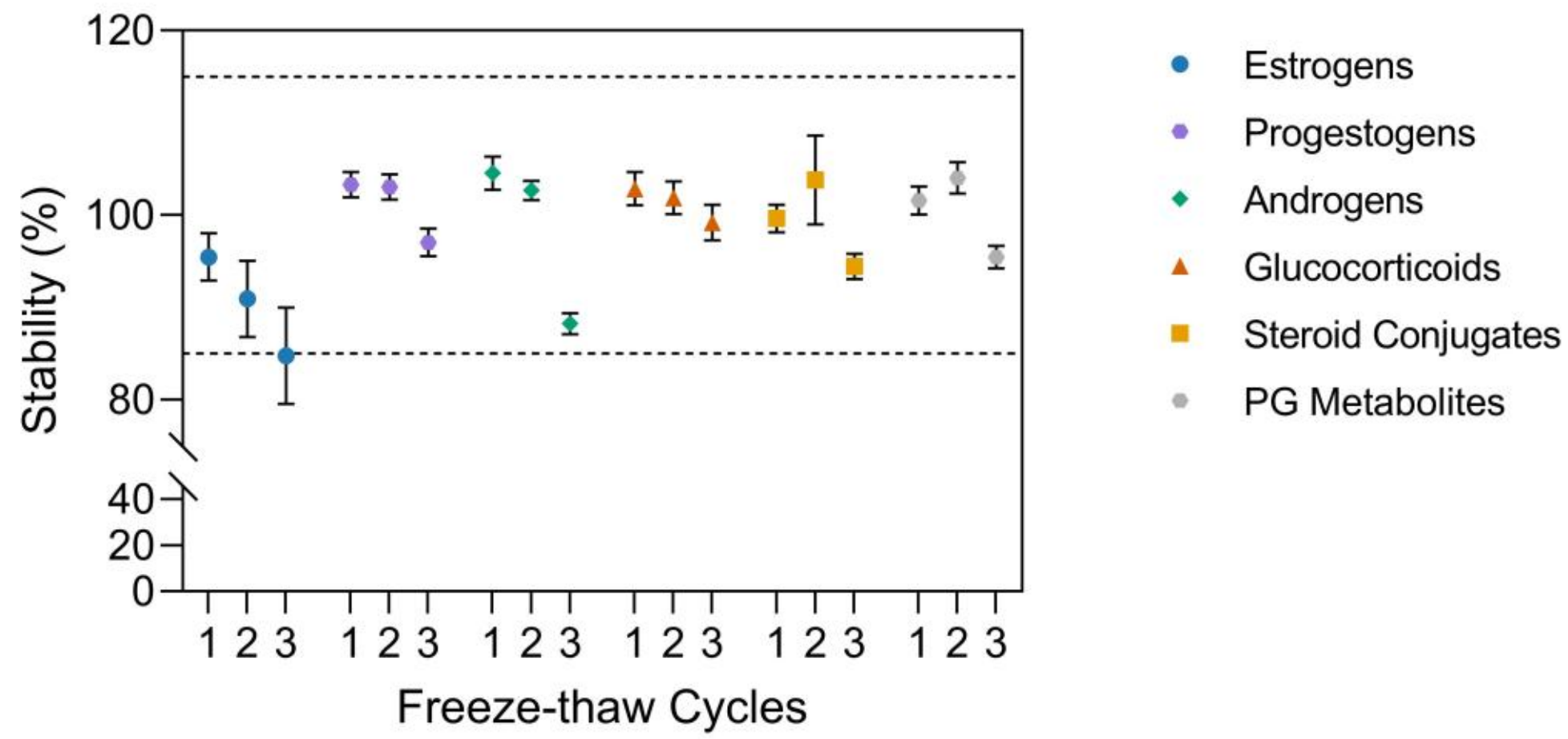

Figure S7. Stability of targets in QC_L and QC_H samples and a cattle plasma sample after 1, 2, and 3 freeze-thaw cycles. Data are shown as mean ± SEM, grouped by target category.

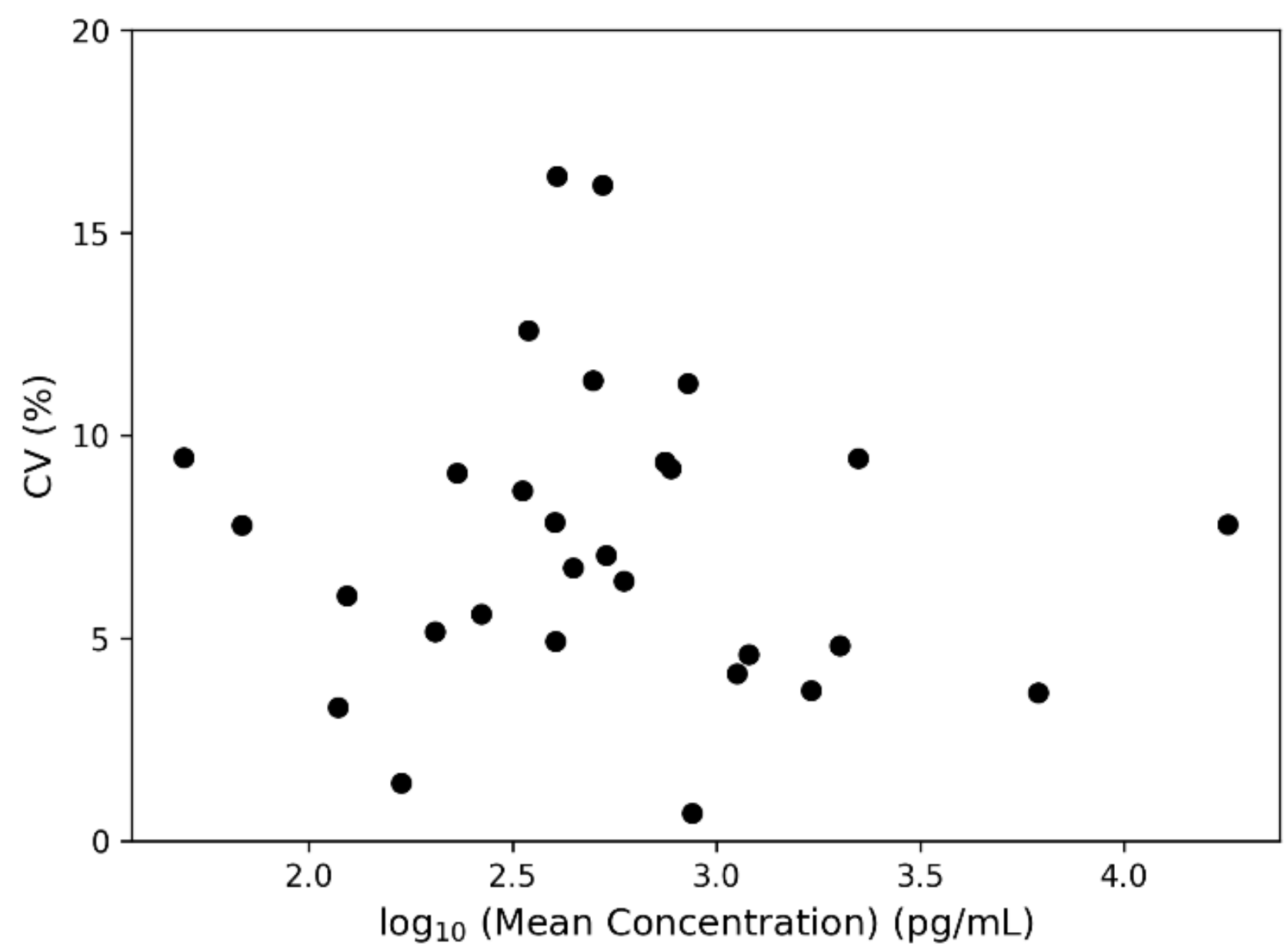

Figure S8. Long-term plasma stability of targets in a cattle plasma sample collected during pregnancy and stored at −20 °C. Each point represents the CV of one target across reanalysis on days 0, 25, and 48.

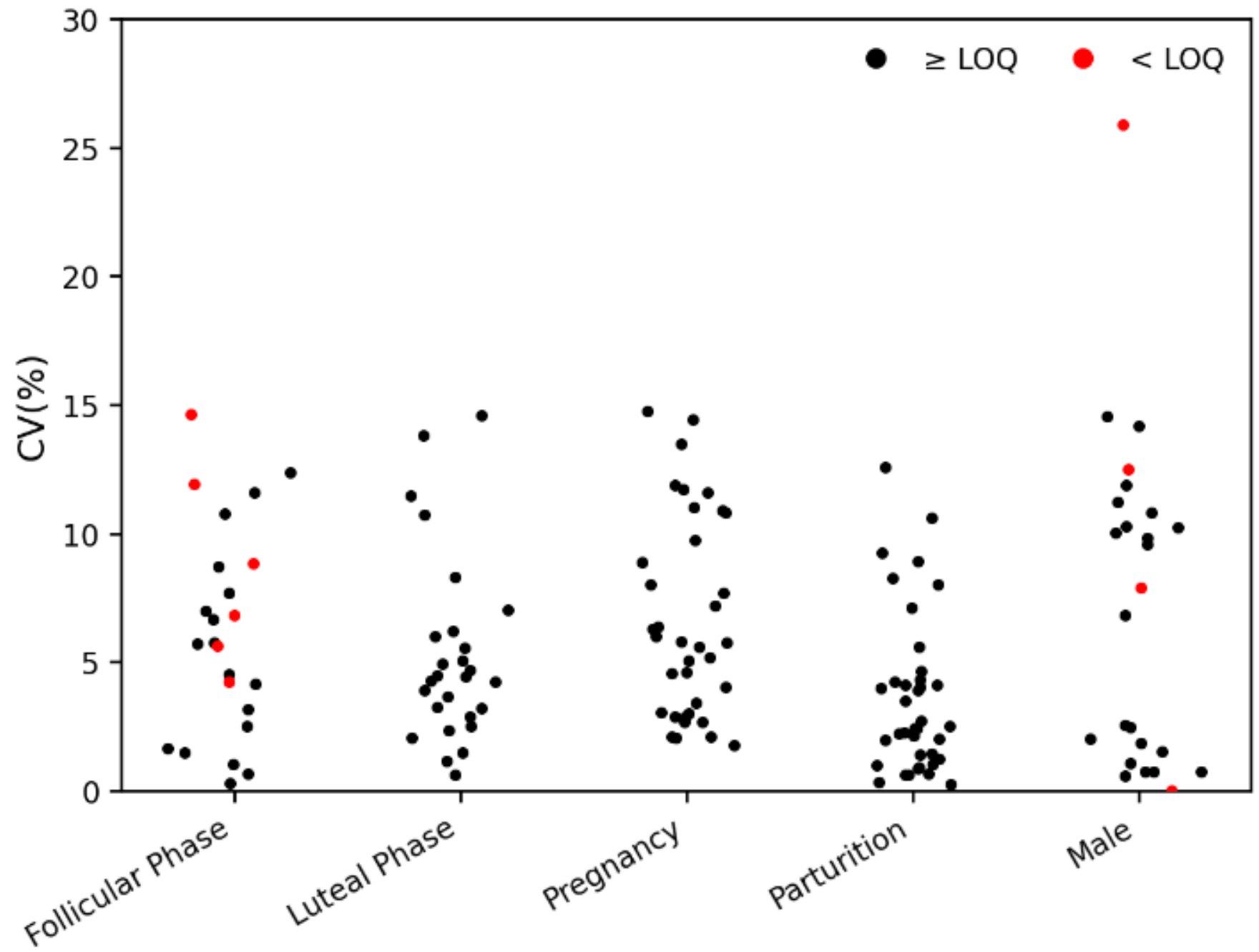

Figure S9. Coefficients of variation (CVs) of quantified targets in cattle plasma samples. Each point represents the CV derived from triplicate preparation and analysis of an individual sample within a given physiological state. Black points indicate targets with mean concentrations above the corresponding LOQs, whereas red points denote those below LOQs.

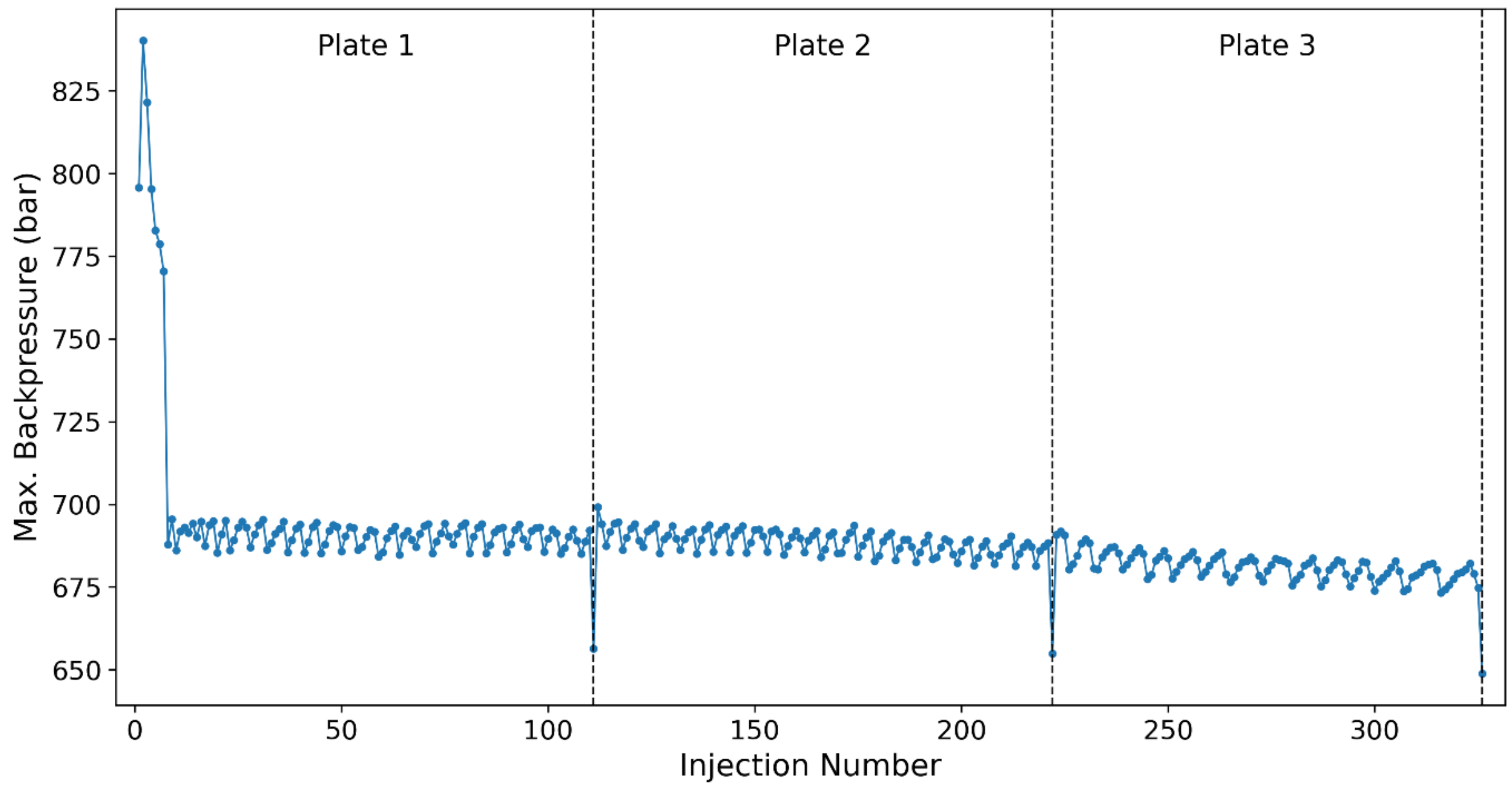

Figure S10. Maximum system backpressure recorded throughout the long-sequence across consecutive measurements of three 96-well plates. Each point represents the maximum system backpressure recorded during one run. The elevated pressures at the beginning of the sequence corresponded to the first set of conditioning injections, whereas the three abrupt decreases corresponded to column cleaning runs performed at a lower flow rate (0.2 mL/min) after each 96-well plate.

Table S1. Summary of target standards, including names, CAS numbers, chemical formulas, retention times (RT), *m/z* of monitored ions, corresponding adducts, ISTDs used, and selection rationale. ER, estrogen receptor; AR, androgen receptor.

Category	Abbreviation	Trivial Name	CAS Number	Name	Formula	RT (min)	Monitored Ion (*m/z*)	Adduct	ISTD	Selection Rationale
Estrogen	E1	Estrone	53-16-7	1, 3, 5(10)-estratrien-3-ol-17-one	$C_{18}H_{22}O_2$	5.30	269.1547	$[M-H]^-$	E1-d4	E2 precursor
	E2	17β-Estradiol	50-28-2	1, 3, 5(10)-estratrien-3,17β-diol	$C_{18}H_{24}O_2$	5.27	271.1704	$[M-H]^-$	E2-d5	Main estrogen
	17α-E2	17α-Estradiol	57-91-0	1, 3, 5(10)-estratrien-3,17α-diol	$C_{18}H_{24}O_2$	5.87	271.1704	$[M-H]^-$	E2-d5	Stereoisomer of E2
	E3	Estriol	50-27-1	1, 3, 5(10)-estratrien-3,16α, 17β-triol	$C_{18}H_{24}O_3$	1.79	287.1653	$[M-H]^-$	E2-d5	Main estrogen
	16β,17α-E3	16,17-Epiestriol	793-89-5	1, 3, 5(10)-estratrien-3,16β, 17α-triol	$C_{18}H_{24}O_3$	1.70	287.1653	$[M-H]^-$	E2-d5	Stereoisomer of E3
	16β,17β-E3	16-Epiestriol	547-81-9	1, 3, 5(10)-estratrien-3,16β, 17β-triol	$C_{18}H_{24}O_3$	2.80	287.1653	$[M-H]^-$	E2-d5	Stereoisomer of E3
	16α,17α-E3	17-Epiestriol	1228-72-4	1, 3, 5(10)-estratrien-3,16α, 17α-triol	$C_{18}H_{24}O_3$	3.07	287.1653	$[M-H]^-$	E2-d5	Stereoisomer of E3
Progestogen	P5	Pregnenolone	145-13-1	5-pregnen-3β-ol-20-one	$C_{21}H_{32}O_2$	7.94	299.2369	$[M-H_2O+H]^+$	P5-d4	P4 precursor
	P4	Progesterone	57-83-0	4-pregnen-3, 20-dione	$C_{21}H_{30}O_2$	8.17	315.2319	$[M+H]^+$	P4-d9	Main progestogen
	17-OHP4	17α-Hydroxyprogesterone	68-96-2	4-pregnen-17-ol-3,20-dione	$C_{21}H_{30}O_3$	6.15	331.2268	$[M+H]^+$	17-OHP4-d8	Neurosteroid
	3α-DHP	3α-Dihydroprogesterone	25680-68-6	4-pregnen-3α-ol-20-one	$C_{21}H_{32}O_2$	8.48	299.2369	$[M-H_2O+H]^+$	P5-d4	Neurosteroid
	3β-DHP	3β-Dihydroprogesterone	566-66-5	4-pregnen-3β-ol-20-one	$C_{21}H_{32}O_2$	7.94	299.2369	$[M-H_2O+H]^+$	P5-d4	Neurosteroid
	5α-DHP	5α-Dihydroprogesterone	566-65-4	5α-pregnan-3,20-dione	$C_{21}H_{32}O_2$	9.49	317.2475	$[M+H]^+$	P5-d4	Neurosteroid
	5β-DHP	5β-Dihydroprogesterone	128-23-4	5β-pregnan-3,20-dione	$C_{21}H_{32}O_2$	9.57	317.2475	$[M+H]^+$	P5-d4	Neurosteroid
	20α-DHP	20α-Dihydroprogesterone	145-14-2	4-pregnen-20α-ol-3-one	$C_{21}H_{32}O_2$	6.98	317.2475	$[M+H]^+$	17-OHP4-d8	Neurosteroid
	20β-DHP	20β-Dihydroprogesterone	145-15-3	4-pregnen-20β-ol-3-one	$C_{21}H_{32}O_2$	8.11	317.2475	$[M+H]^+$	17-OHP4-d8	Neurosteroid
	3β,5α-THP	3β,5α-Tetrahydroprogesterone	516-55-2	5α-pregnan-3β-ol-20-one	$C_{21}H_{34}O_2$	8.47	301.2526	$[M-H_2O+H]^+$	P5-d4	Neurosteroid
	3β,5β-THP	3β,5β-Tetrahydroprogesterone	128-21-2	5β-pregnan-3β-ol-20-one	$C_{21}H_{34}O_2$	8.66	301.2526	$[M-H_2O+H]^+$	P5-d4	Neurosteroid
	3α,5β-THP	Pregnanolone	128-20-1	5β-pregnan-3α-ol-20-one	$C_{21}H_{34}O_2$	8.85	301.2526	$[M-H_2O+H]^+$	P5-d4	Neurosteroid
	3α,5α-THP	3α,5α-Tetrahydroprogesterone	516-54-1	5α-pregnan-3α-ol-20-one	$C_{21}H_{34}O_2$	9.25	301.2526	$[M-H_2O+H]^+$	P5-d4	Neurosteroid
Androgen	DHEA	Dehydroepiandrosterone	53-43-0	5-androsten-3β-ol-17-one	$C_{19}H_{28}O_2$	5.82	271.2056	$[M-H_2O+H]^+$	A4-d7	T precursor, neurosteroid
	5-A5	5-Androstenediol	521-17-5	5-androsten-3β,17β-diol	$C_{19}H_{30}O_2$	4.87	273.2213	$[M-H_2O+H]^+$	T-d3	ER affinity
	4-A5	4-Androstenediol	1156-92-9	4-androsten-3β,17β-diol	$C_{19}H_{30}O_2$	4.87	273.2213	$[M-H_2O+H]^+$	T-d3	Stereoisomer of 5-A5
	A4	Androstenedione	63-05-8	4-androsten-3,17-dione	$C_{19}H_{26}O_2$	5.95	287.2006	$[M+H]^+$	A4-d7	T precursor
	T	Testosterone	58-22-0	4-androsten-17β-ol-3-one	$C_{19}H_{28}O_2$	5.28	289.2162	$[M+H]^+$	T-d3	Main androgen
	5α-AON	5α-Androstanedione	846-46-8	5α-androstan-3,17-dione	$C_{19}H_{28}O_2$	7.29	289.2162	$[M+H]^+$	AS-d2	A4 metabolite
	5β-AON	5β-Androstanedione	1229-12-5	5β-androstan-3,17-dione	$C_{19}H_{28}O_2$	7.47	289.2162	$[M+H]^+$	AS-d2	Stereoisomer of 5α-AON
	5α-DHT	5α-Dihydrotestosterone	521-18-6	5α-androstan-17β-ol-3-one	$C_{19}H_{30}O_2$	6.52	291.2319	$[M+H]^+$	AS-d2	Potent androgen
	5β-DHT	5β-Dihydrotestosterone	571-22-2	5β-androstan-17β-ol-3-one	$C_{19}H_{30}O_2$	6.73	291.2319	$[M+H]^+$	AS-d2	Stereoisomer of 5α-DHT
	3β,5α-AS	Epiandrosterone	481-29-8	5α-androstan-3β-ol-17-one	$C_{19}H_{30}O_2$	6.26	273.2213	$[M-H_2O+H]^+$	AS-d2	Neurosteroid

	3β,5β-AS	Epietiocholanolone	571-31-3	5β-androstan-3β-ol-17-one	$C_{19}H_{30}O_2$	6.56	273.2213	$[M-H_2O+H]^+$	AS-d2	Neurosteroid
	3α,5β-AS	Etiocholanolone	53-42-9	5β-androstan-3α-ol-17-one	$C_{19}H_{30}O_2$	7.12	273.2213	$[M-H_2O+H]^+$	AS-d2	Neurosteroid
	3α,5α-AS	Androsterone	53-41-8	5α-androstan-3α-ol-17-one	$C_{19}H_{30}O_2$	7.42	273.2213	$[M-H_2O+H]^+$	AS-d2	Neurosteroid
	3β,5α-DIOL	5α-Androstane-3β,17β-diol	571-20-0	5α-androstan-3β,17β-diol	$C_{19}H_{32}O_2$	5.50	257.2264	$[M-2H_2O+H]^+$	AS-d2	ER affinity
	3β,5β-DIOL	5β-Androstane-3β,17β-diol	6038-31-9	5β-androstan-3β,17β-diol	$C_{19}H_{32}O_2$	5.70	257.2264	$[M-2H_2O+H]^+$	AS-d2	ER affinity
	3α,5β-DIOL	5β-Androstane-3α,17β-diol	1851-23-6	5β-androstan-3α,17β-diol	$C_{19}H_{32}O_2$	6.35	257.2264	$[M-2H_2O+H]^+$	AS-d2	Stereoisomer of 3β,5α-DIOL
	3α,5α-DIOL	5α-Androstane-3α,17β-diol	1852-53-5	5α-androstan-3α,17β-diol	$C_{19}H_{32}O_2$	6.50	257.2264	$[M-2H_2O+H]^+$	AS-d2	ER affinity, neurosteroid
	B	Corticosterone	50-22-6	4-pregnen-11β,21-diol-3,20-dione	$C_{21}H_{30}O_4$	3.97	391.2126	$[M+HCOO]^-$	F-d4	Glucocorticoid synthesized from P4
	F	Cortisol	50-23-7	4-pregnen-11β,17,21-triol-3, 20-dione	$C_{21}H_{30}O_5$	2.79	407.2075	$[M+HCOO]^-$	F-d4	Main glucocorticoid
	A	11-Dehydrocorticosterone	72-23-1	4-pregnen-21-ol-3,11,20-trione	$C_{21}H_{28}O_4$	3.67	389.1970	$[M+HCOO]^-$	F-d4	B metabolite
	E	Cortisone	53-06-5	4-pregnen-17,21-diol-3,11,20-trione	$C_{21}H_{28}O_5$	2.87	405.1919	$[M+HCOO]^-$	F-d4	F metabolite
	3β,5α-THF	3β,5α-Tetrahydrocortisol	651-43-4	5α-pregnan-3β,11β,17,21-tetrol-20-one	$C_{21}H_{34}O_5$	2.35	411.2388	$[M+HCOO]^-$	F-d4	Neurosteroid
Glucocorticoid	3β,5β-THF	3β,5β-Tetrahydrocortisol	15734-50-6	5β-pregnan-3β,11β,17,21-tetrol-20-one	$C_{21}H_{34}O_5$	2.48	411.2388	$[M+HCOO]^-$	F-d4	Neurosteroid
	3α,5α-THF	3α,5α-Tetrahydrocortisol	302-91-0	5α-pregnan-3α,11β,17,21-tetrol-20-one	$C_{21}H_{34}O_5$	3.31	411.2388	$[M+HCOO]^-$	F-d4	Neurosteroid
	3α,5β-THF	3α,5β-Tetrahydrocortisol	53-02-1	5β-pregnan-3α,11β,17,21-tetrol-20-one	$C_{21}H_{34}O_5$	3.35	411.2388	$[M+HCOO]^-$	F-d4	Neurosteroid
	3β,5α-THE	3β,5α-Tetrahydrocortisone	516-45-0	5α-pregnan-3β,17,21-triol-11,20-dione	$C_{21}H_{32}O_5$	2.40	409.2232	$[M+HCOO]^-$	F-d4	Neurosteroid
	3α,5α-THE	3α,5α-Tetrahydrocortisone	547-77-3	5α-pregnan-3α,17,21-triol-11,20-dione	$C_{21}H_{32}O_5$	3.58	409.2232	$[M+HCOO]^-$	F-d4	Neurosteroid
	3α,5β-THE	3α,5β-Tetrahydrocortisone	53-05-4	5β-pregnan-3α,17,21-triol-11,20-dione	$C_{21}H_{32}O_5$	3.65	409.2232	$[M+HCOO]^-$	F-d4	Neurosteroid
	E1-S	Estrone-3-sulfate	481-97-0	1, 3, 5(10)-estratrien-3-ol-17-one sulphate	$C_{18}H_{22}O_5S$	3.37	349.1115	$[M-H]^-$	E2-3-S-d4	Potential reservoir
	E2-17-S	17β-Estradiol-17-sulfate	3233-69-0	1, 3, 5(10)-estratrien-3,17β-diol 17-sulphate	$C_{18}H_{24}O_5S$	2.54	351.1272	$[M-H]^-$	E2-3-S-d4	Potential reservoir
	E2-3-S	17β-Estradiol-3-sulfate	481-96-9	1, 3, 5(10)-estratrien-3,17β-diol 3-sulphate	$C_{18}H_{24}O_5S$	2.72	351.1272	$[M-H]^-$	E2-3-S-d4	Potential reservoir
Steroid Conjugate	E3-3-S	Estriol-3-sulfate	481-95-8	1, 3, 5(10)-estratrien-3,16α, 17β-triol 3-sulphate	$C_{18}H_{24}O_6S$	0.95	367.1221	$[M-H]^-$	E2-3-S-d4	Potential reservoir
	DHEA-S	Dehydroepiandrosterone sulfate	651-48-9	5-androsten-3β-ol-17-one-16 sulphate	$C_{19}H_{28}O_5S$	3.65	367.1585	$[M-H]^-$	E2-3-S-d4	Neurosteroid, ER/AR affinity
	E1-G	Estrone-3-glucuronide	2479-90-5	1, 3, 5(10)-estratrien-3-ol-17-one glucosiduronate	$C_{24}H_{30}O_8$	2.80	445.1868	$[M-H]^-$	E2-3-G-d3	Potential reservoir
	E2-3-G	17β-Estradiol-3-glucuronide	15270-30-1	1, 3, 5(10)-estratrien-3,17β-diol 3-glucosiduronate	$C_{24}H_{32}O_8$	2.16	447.2024	$[M-H]^-$	E2-3-G-d3	Potential reservoir
	E2-17-G	17β-Estradiol-17-glucuronide	1806-98-0	1, 3, 5(10)-estratrien-3,17β-diol 17-glucosiduronate	$C_{24}H_{32}O_8$	2.70	447.2024	$[M-H]^-$	E2-3-G-d3	Potential reservoir
	PGE2m	13,14-dihydro-15-keto-PGE2	363-23-5	(5z,11α)-11-hydroxy-9,15-dioxo-prost-5-en-1-oic acid	$C_{20}H_{32}O_5$	5.10	335.2217	$[M-H_2O+H]^+$	PGE1m-d4	Ovulation and parturition related
	PGF2αm	13,14-dihydro-15-keto-PGF2α	27376-76-7	9α,11α-dihydroxy-15-oxo-prost-5z-en-1-oic acid	$C_{20}H_{34}O_5$	4.98	337.2373	$[M-H_2O+H]^+$	PGF2αm-d4	Luteolysis and parturition related
PG Metabolite	PGD1m	13,14-dihydro-15-keto-PGD1	1392219-79-2	9α-hydroxy-11,15-dioxo-prost-1-oic acid	$C_{20}H_{34}O_5$	5.83	355.2479	$[M+H]^+$	PGE1m-d4	PG homologue
	PGE1m	13,14-dihydro-15-keto-PGE1	5094-14-4	11α-hydroxy-9,15-dioxo-prostan-1-oic acid	$C_{20}H_{34}O_5$	5.38	337.2373	$[M-H_2O+H]^+$	PGE1m-d4	PG homologue
	PGF1αm	13,14-dihydro-15-keto-PGF1α	29044-75-5	9α,11α-dihydroxy-15-oxo-prostan-1-oic acid	$C_{20}H_{36}O_5$	5.27	339.2530	$[M-H_2O+H]^+$	PGF2αm-d4	PG homologue

Table S2. Summary of ISTDs, including names, CAS numbers, chemical formulas, retention times (RT), *m/z* of monitored ions, and corresponding adducts.

Category	Abbreviation	Trivial Name	CAS Number	Name	Formula	RT (min)	Monitored Ion (*m/z*)	Adduct
Estrogen	E1-d4	Estrone-d4	53866-34-5	1, 3, 5(10)-estratrien-3-ol-17-one-2,4,16,16-d_4	$C_{18}H_{18}D_4O_2$	5.33	273.1798	$[M-H]^-$
	E2-d5	17β-Estradiol-d5	221093-45-4	1, 3, 5(10)-estratrien-3,17-diol-2,4,16,16,17-d_5	$C_{18}H_{19}D_5O_2$	5.28	276.2017	$[M-H]^-$
Progestogen	P5-d4	Pregnenolone-d4	61574-54-7	5-pregnen-3β-ol-20-one-17,21,21,21-d_4	$C_{21}H_{28}D_4O_2$	7.95	303.2620	$[M-H_2O+H]^+$
	P4-d9	Progesterone-d9	15775-74-3	4-pregnen-3,20-dione-2,2,4,6,6,17α,21,21,21-d_9	$C_{21}H_{21}D_9O_2$	8.14	324.2883	$[M+H]^+$
	17-OHP4-d8	17α-Hydroxyprogesterone-d8	850023-80-2	4-pregnen-17-ol-3,20-dione-2,2,4,6,6,21,21,21-d_8	$C_{21}H_{22}D_8O_3$	6.18	339.2770	$[M+H]^+$
Androgen	A4-d7	Androstenedione-d7	67034-85-9	4-androsten-3, 17-dione-2,2,4,6,6,16,16-d_7	$C_{19}H_{19}D_7O_2$	5.97	294.2445	$[M+H]^+$
	T-d3	Testosterone-d3	77546-39-5	4-androsten-17β-ol-3-one-16,16,17-d_3	$C_{19}H_{25}D_3O_2$	5.29	292.2350	$[M+H]^+$
	AS-d2	3α,5α-Androsterone-d2	89685-22-3	5α-androstan-3α-ol-17-one-16,16-d_2	$C_{19}H_{28}D_2O_2$	7.45	275.2339	$[M-H_2O+H]^+$
Glucocorticoid	F-d4	Cortisol-d4	73565-87-4	4-pregnen-11β,17,21-triol-3,20-dione-9,11,12,12-d_4	$C_{21}H_{26}D_4O_5$	2.82	411.2326	$[M+HCOO]^-$
Steroid Conjugate	E2-3-S-d4	17β-Estradiol-3-sulfate-d4	744189-67-1	1, 3, 5(10)-estratrien-3,17β-diol 3-sulphate-2,4,16,16-d_4	$C_{18}H_{20}D_4O_5S$	2.73	355.1523	$[M-H]^-$
	E2-3-G-d3	17β-Estradiol-3-glucuronide-d3	1260231-06-8	1, 3, 5(10)-estratrien-3,17β-diol 3-glucosiduronate-16,16,17-d_3	$C_{24}H_{29}D_3O_8$	2.18	450.2213	$[M-H]^-$
PG metabolite	PGF2αm-d4	13,14-dihydro-15-keto-PGF2α-d4	42932-59-2	9α,11α-dihydroxy-15-oxo-prost-5z-en-1-oic acid-3,3,4,4-d_4	$C_{20}H_{30}D_4O_5$	4.98	341.2624	$[M-H_2O+H]^+$
	PGE1m-d4	13,14-dihydro-15-keto-PGE1-d4	NA	11α-hydroxy-9,15-dioxo-prostan-1-oic acid-3,3,4,4-d_4	$C_{20}H_{30}D_4O_5$	5.41	341.2624	$[M-H_2O+H]^+$

Table S3a. Evaluation of SPE sorbents according to the manufacturer's protocol recommended for steroid extraction. Variables between the procedures are highlighted in bold.

Procedure	Oasis MAX	Strata-X Pro	Oasis HLB
Sample	- - - - - - - - - - - - - - - - 250 µL plasma - - - - - - - - - - - - - - - -		
Dilution	250 µL aq. H_3PO_4 (4%)	250 µL aq. FA (1%)	250 µL aq. H_3PO_4 (4%)
SPE Plate	**Oasis MAX**	**Strata-X Pro**	**Oasis HLB**
Condition	500 µL MeOH	1000 µL MeOH	500 µL MeOH
Equilibration	500 µL aq. H_3PO_4 (4%)	1000 µL H_2O	500 µL aq. H_3PO_4 (4%)
Loading	- - - - - - - - - - - - - - - - Diluted sample - - - - - - - - - - - - - - - -		
Washing 1	500 µL aq. NH_3 (5%)	1000 µL aq. FA (1%)	500 µL aq. MeOH (5%)
Washing 2	500 µL aq. MeOH (5%)	1000 µL aq. MeOH (30%)	-
Elution 1	500 µL MeOH	500 µL MeOH/MeCN (1:4)	500 µL MeOH
Elution 2	500 µL MeOH + 2% FA	500 µL MeOH/MeCN (1:4)	-
Drying	- - - - - - - - - - - - - - - - Under a N_2 stream at 45 °C - - - - - - - - - - - - - - - -		
Reconstitution	- - - - - - - - - - - - - - - - 100 µL aq. MeOH (40%) - - - - - - - - - - - - - - - -		

Table S3b. Optimization of additives used during PPT. Variables between the procedures are highlighted in bold.

Procedure	Control Group (No Additive)	H_3PO_4	PK
Sample	- - - - - - - - - - - - - - - - 250 µL plasma - - - - - - - - - - - - - - - -		
PPT Pretreatment	125 µL aqueous (aq.) $ZnSO_4$ (0.1 M) and 250 µL MeOH	125 µL aq. $ZnSO_4$ (0.1 M), 250 µL MeOH and **aq. H_3PO_4 (10%)**	5 µL **PK (20 mg/mL)** and **5 µL aq. $CaCl_2$ (0.25 M)**<br>125 µL aq. $ZnSO_4$ (0.1 M) and 250 µL MeOH
Dilution	- - - - - - - - - - - - - - - - Take 375 µL supernatant, dilute with 225 µL H_2O - - - - - - - - - - - - - - - -		
SPE Plate	- - - - - - - - - - - - - - - - Oasis HLB - - - - - - - - - - - - - - - -		
Condition	- - - - - - - - - - - - - - - - 500 µL MeCN - - - - - - - - - - - - - - - -		
Equilibration	- - - - - - - - - - - - - - - - 500 µL H_2O - - - - - - - - - - - - - - - -		
Loading	- - - - - - - - - - - - - - - - Pretreated sample - - - - - - - - - - - - - - - -		
Washing	- - - - - - - - - - - - - - - - 500 µL aq. MeCN (5%) - - - - - - - - - - - - - - - -		
Elution	- - - - - - - - - - - - - - - - 500 µL MeCN - - - - - - - - - - - - - - - -		
Drying	- - - - - - - - - - - - - - - - Under a N_2 stream at 45 °C - - - - - - - - - - - - - - - -		
Reconstitution	- - - - - - - - - - - - - - - - 100 µL aq. MeOH (40%) - - - - - - - - - - - - - - - -		

Table S3c. Optimization of PPT solvents and supernatant dilution factors. Variables between the procedures are highlighted in bold.

Procedure	MeCN, 1.6× with H_2O	MeOH, ∞ (Complete Evaporation of the PPT solvent)	MeOH, 1.6× with H_2O
Sample	250 µL plasma		
PPT Pretreatment	125 µL aq. $ZnSO_4$ (0.1 M) and 250 µL **MeCN**	125 µL aq. $ZnSO_4$ (0.1 M) and 250 µL **MeOH**	125 µL aq. $ZnSO_4$ (0.1 M) and 250 µL **MeOH**
Dilution	Take 375 µL supernatant, **dilute with 225 µL H_2O**	Take 375 µL supernatant, **concentrate under a N_2 stream at 45 °C for 30 min**	Take 375 µL supernatant, **dilute with 225 µL H_2O**
SPE Plate	Oasis HLB		
Condition	500 µL MeCN		
Equilibration	500 µL H_2O		
Loading	Pretreated sample		
Washing	500 µL aq. MeCN (5%)		
Elution	500 µL MeCN		
Drying	Under a N_2 stream at 45 °C		
Reconstitution	100 µL aq. MeOH (40%)		

Table S3d. Optimization of SPE washing/elution solvents. Variables between the procedures are highlighted in bold.

Procedure	MeOH	MeCN	EtOAc
Sample	250 µL plasma		
Pretreatment	250 µL aq. H_3PO_4 (4%)		
SPE Plate	Oasis HLB		
Condition	500 µL **MeOH**	500 µL **MeCN**	500 µL **EtOAc** and 500 µL **MeOH**
Equilibration	500 µL aq. H_3PO_4 (4%)		
Loading	Pretreated sample		
Washing	500 µL aq. **MeOH** (5%)	500 µL aq. **MeCN** (5%)	500 µL aq. **EtOAc** (5%)
Elution	500 µL **MeOH**	500 µL **MeCN**	500 µL **EtOAc**
Drying	Under a N_2 stream at 45 °C		
Reconstitution	100 µL aq. MeOH (40%)		

Table S4. Method validation results for each target using SPE determined in charcoal-stripped plasma. LOQs and LODs are presented in pg/mL; recovery and matrix effects are shown as mean (SD) in %, accuracy are expressed as mean absolute relative error (SD) in %; intraday and interday precision are shown as CV in %.

Analyte	LOQ (pg/mL)	LOD (pg/mL)	Recovery (%)			Matrix Effect (%)			Accuracy (%)			Intraday Precision (%)			Interday Precision (%)		
			QC_L	QC_M	QC_H	QC_L	QC_M	QC_H	QC_L	QC_M	QC_H	QC_L	QC_M	QC_H	QC_L	QC_M	QC_H
E1	20	10	97.7 (8.4)	89.7 (8.3)	74.8 (3.1)	91.1 (2.8)	97.2 (2.0)	98.0 (2.4)	3.8 (1.0)	3.0 (1.5)	8.2 (3.2)	0.5	4.0	1.9	1.0	3.3	3.0
E2	20	5	92.1 (4.9)	85.7 (8.3)	71.7 (2.1)	92.5 (5.3)	98.7 (1.6)	99.1 (2.5)	3.5 (2.7)	4.7 (3.1)	10.7 (4.0)	0.8	3.5	0.8	2.8	5.6	3.6
17α-E2	20	5	93.1 (5.6)	87.1 (7.8)	71.8 (1.2)	90.4 (2.1)	95.9 (1.1)	100.2 (5.7)	1.1 (0.8)	5.2 (3.8)	9.5 (4.2)	1.0	3.0	1.8	1.1	6.4	3.8
E3	50	20	107.1 (7.7)	100.2 (9.6)	88.3 (4.7)	86.1 (6.0)	89.5 (7.3)	92.8 (8.1)	4.2 (2.1)	9.7 (1.6)	10.5 (3.1)	4.0	3.5	4.0	4.7	8.9	2.8
16β,17α-E3	50	20	110.1 (9.8)	101.8 (9.9)	78.6 (12.0)	87.2 (7.9)	92.4 (6.4)	94.3 (5.5)	3.3 (0.8)	4.7 (4.3)	12.3 (6.7)	2.7	3.1	3.4	3.2	5.8	10.5
16β,17β-E3	20	10	105.1 (8.6)	95.1 (8.1)	79.6 (3.0)	84.9 (1.5)	91.3 (1.4)	95.4 (4.8)	2.1 (2.0)	6.6 (2.7)	9.9 (5.4)	2.7	4.2	3.6	2.7	5.1	4.9
16α,17α-E3	20	10	112.7 (9.1)	92.7 (10.0)	81.3 (3.3)	96.3 (11.2)	102.2 (9.3)	97.5 (3.6)	1.4 (0.8)	3.9 (2.1)	10.7 (6.0)	3.0	3.1	1.5	1.5	4.5	5.4
P5	20	10	93.8 (10.8)	75.5 (6.1)	66.6 (3.7)	103.9 (6.0)	107.3 (0.6)	104.5 (0.9)	3.3 (1.1)	4.7 (2.1)	9.0 (3.5)	2.6	4.5	1.8	3.5	5.1	3.2
P4	20	5	95.8 (8.9)	86.7 (6.7)	75.4 (1.6)	92.0 (3.2)	95.8 (3.0)	95.5 (1.5)	3.9 (0.7)	5.2 (1.2)	7.7 (3.2)	3.1	5.7	1.5	0.8	5.2	3.0
17-OHP4	20	5	110.9 (13.3)	98.5 (9.4)	88.4 (2.5)	94.6 (3.0)	100.8 (3.1)	98.6 (4.3)	3.3 (0.7)	3.4 (2.1)	8.4 (5.9)	0.8	4.1	1.4	0.7	3.5	6.0
3α-DHP	50	20	84.1 (13.2)	75.2 (5.6)	63.7 (3.7)	99.8 (4.9)	106.0 (2.5)	101.0 (1.5)	6.4 (3.7)	4.5 (1.5)	10.1 (2.3)	2.7	2.3	1.8	6.1	1.6	2.1
3β-DHP	20	10	84.9 (10.5)	75.4 (6.1)	63.6 (3.0)	103.9 (6.1)	107.2 (0.6)	104.5 (1.0)	5.6 (3.7)	5.9 (3.5)	8.3 (2.5)	3.3	1.9	6.1	6.7	5.7	2.3
5α-DHP	100	50	79.7 (14.2)	71.3 (5.3)	59.7 (3.8)	95.4 (5.7)	98.5 (2.1)	97.6 (1.7)	8.1 (2.0)	8.1 (5.5)	9.4 (7.1)	17.2	6.0	5.5	7.6	6.0	6.5
5β-DHP	100	50	79.6 (17.6)	69.0 (8.3)	61.0 (4.4)	89.6 (3.2)	98.5 (2.2)	100.5 (4.3)	8.0 (1.5)	6.4 (4.1)	9.7 (2.2)	10.3	7.6	7.2	8.2	7.5	2.0
20α-DHP	50	10	89.3 (8.6)	95.3 (2.6)	78.7 (4.1)	65.8 (2.2)	64.4 (5.9)	69.0 (2.7)	5.9 (2.6)	8.5 (5.4)	8.5 (3.0)	2.5	6.9	9.8	2.8	10.1	6.7
20β-DHP	20	5	93.7 (9.5)	85.9 (5.9)	72.2 (2.4)	95.5 (2.8)	97.3 (3.1)	100.7 (0.7)	9.7 (6.1)	6.0 (4.9)	11.0 (4.4)	3.6	6.1	4.3	6.8	14.4	4.2
3β,5α-THP	50	20	79.2 (15.8)	69.3 (6.0)	57.6 (3.6)	102.7 (0.9)	112.3 (6.4)	107.2 (4.2)	11.2 (7.4)	7.8 (3.5)	6.2 (4.5)	10.2	3.6	9.3	8.3	3.8	6.5
3β,5β-THP	100	20	77.0 (11.0)	67.5 (5.3)	58.1 (4.1)	102.7 (7.5)	110.6 (3.9)	104.4 (4.2)	7.7 (1.9)	5.6 (1.5)	12.8 (1.4)	5.6	5.6	2.5	7.8	5.8	1.2
3α,5β-THP	100	50	77.8 (11.4)	74.0 (6.0)	57.8 (3.8)	105.4 (4.1)	109.9 (1.8)	109.1 (2.8)	10.4 (6.3)	4.4 (2.6)	13.2 (2.0)	18.5	7.7	4.1	7.0	5.0	1.8
3α,5α-THP	100	20	72.2 (12.0)	64.5 (5.7)	53.2 (3.1)	100.2 (5.8)	108.2 (2.0)	106.6 (0.3)	5.3 (1.9)	6.3 (1.4)	9.1 (3.1)	16.8	13.1	4.7	5.5	6.2	2.8
DHEA	50	20	101.8 (15.8)	95.0 (10.4)	81.7 (3.1)	99.2 (7.9)	97.7 (4.5)	97.5 (2.0)	7.4 (3.0)	5.3 (1.6)	9.2 (5.5)	4.8	3.7	10.6	6.4	4.9	6.8
5-A5	50	20	102.9 (10.4)	96.8 (9.2)	83.4 (3.2)	94.3 (4.0)	93.6 (2.7)	96.6 (2.1)	3.8 (3.2)	5.4 (0.9)	11.6 (2.5)	3.9	4.3	4.8	4.4	4.7	2.2
4-A5	50	20	103.0 (10.5)	96.8 (9.2)	83.4 (3.2)	93.9 (4.6)	93.6 (2.7)	96.5 (2.0)	2.8 (1.5)	5.4 (1.2)	9.9 (3.5)	3.8	4.5	5.9	3.1	4.8	3.1
A4	50	20	111.1 (13.1)	99.0 (6.9)	88.1 (3.4)	90.7 (4.5)	91.5 (2.2)	94.2 (1.4)	4.4 (2.4)	3.1 (1.9)	9.2 (3.9)	5.8	3.8	5.4	4.9	2.4	3.6
T	20	5	111.0 (11.5)	102.9 (5.4)	88.9 (3.5)	88.4 (5.3)	87.6 (4.3)	85.3 (0.6)	5.0 (0.9)	5.0 (1.2)	10.0 (3.5)	0.7	4.8	1.9	0.9	5.1	3.2
5α-AON	200	50	118.3 (21.5)	93.9 (5.3)	78.6 (4.0)	99.8 (14.9)	91.9 (4.8)	98.7 (1.2)	7.8 (4.7)	12.2 (7.1)	9.3 (4.8)	3.0	8.0	14.1	5.1	13.4	10.2
5β-AON	200	100	108.1 (17.2)	96.1 (12.2)	79.1 (4.0)	117.6 (19.6)	105.3 (2.6)	102.4 (2.4)	10.7 (8.9)	6.0 (6.6)	9.9 (4.9)	6.1	9.4	3.5	10.7	14.7	4.5
5α-DHT	50	20	107.8 (15.5)	90.9 (7.7)	78.5 (3.9)	85.8 (7.4)	94.5 (1.8)	95.4 (2.4)	15.7 (2.9)	4.4 (1.3)	4.0 (1.8)	4.6	6.5	3.1	16.1	4.2	3.4
5β-DHT	100	50	106.0 (15.8)	92.3 (7.0)	80.6 (4.4)	99.9 (6.7)	103.7 (2.9)	102.6 (1.9)	10.0 (2.9)	4.0 (3.4)	4.7 (3.6)	7.4	2.7	11.8	8.9	5.0	5.3
3β,5α-AS	50	50	108.5 (16.7)	91.2 (7.1)	80.4 (4.1)	92.1 (9.1)	94.1 (4.4)	96.0 (0.9)	5.4 (6.1)	5.4 (3.3)	3.7 (1.5)	7.9	6.7	1.5	7.2	6.0	3.0

3β,5β-AS	200	50	108.0 (18.4)	92.4 (7.3)	80.7 (3.2)	88.1 (8.2)	92.4 (1.4)	94.8 (1.8)	8.0 (4.1)	6.1 (2.7)	9.1 (2.6)	7.0	4.4	9.1	9.0	5.8	2.4
3α,5β-AS	100	50	111.3 (16.9)	93.1 (8.1)	80.8 (4.3)	97.9 (6.9)	105.8 (0.3)	108.2 (2.3)	10.4 (6.1)	9.1 (0.6)	7.2 (4.4)	3.0	4.5	5.6	6.8	8.7	8.1
3α,5α-AS	50	5	103.1 (15.8)	89.9 (6.3)	77.5 (3.3)	102.6 (7.2)	103.4 (1.7)	103.2 (1.1)	9.2 (2.6)	4.0 (1.8)	6.9 (4.2)	1.0	4.5	9.7	7.4	4.3	3.9
3β,5α-DIOL	50	50	98.9 (15.2)	93.1 (7.0)	81.7 (4.1)	96.5 (5.8)	96.9 (2.0)	95.2 (2.9)	5.5 (3.4)	3.9 (1.9)	8.8 (3.9)	6.2	3.4	5.2	6.1	10.0	3.6
3β,5β-DIOL	50	50	98.9 (11.0)	91.7 (10.7)	79.1 (3.2)	96.6 (6.3)	95.4 (1.0)	96.4 (1.7)	5.7 (1.3)	1.6 (1.9)	9.3 (5.7)	4.6	2.7	11.3	12.3	2.1	6.4
3α,5β-DIOL	50	50	99.7 (16.8)	90.5 (7.7)	76.6 (3.9)	90.3 (7.8)	92.0 (2.6)	96.0 (1.8)	12.5 (2.6)	7.8 (5.0)	2.5 (1.4)	5.4	6.0	0.7	12.0	9.2	2.9
3α,5α-DIOL	50	50	106.0 (13.7)	83.9 (8.1)	78.0 (3.3)	86.0 (3.4)	97.9 (4.3)	94.6 (2.6)	9.4 (3.4)	4.6 (0.5)	8.6 (4.3)	1.1	3.5	10.5	10.0	4.6	7.4
B	50	20	120.8 (10.0)	99.7 (7.5)	91.5 (3.0)	88.9 (3.8)	93.1 (1.7)	93.3 (0.7)	5.1 (3.0)	10.7 (5.0)	9.6 (5.1)	4.8	8.2	2.4	5.8	7.9	4.7
F	50	10	118.1 (12.5)	102.8 (7.1)	96.1 (4.6)	81.4 (0.8)	84.3 (2.9)	86.9 (1.4)	2.5 (1.1)	2.8 (2.0)	8.0 (3.6)	1.2	4.8	0.8	1.2	2.8	3.4
A	50	20	122.0 (11.2)	102.2 (9.3)	91.7 (4.2)	89.5 (5.5)	94.6 (2.1)	94.9 (2.1)	7.3 (0.7)	5.6 (5.0)	2.8 (3.4)	0.7	7.9	2.1	7.1	6.8	3.3
E	50	10	121.0 (12.0)	104.5 (8.3)	93.8 (3.0)	91.1 (3.3)	96.1 (2.0)	99.6 (1.2)	2.8 (1.7)	5.3 (2.5)	6.5 (4.5)	4.8	6.8	1.1	1.8	5.1	4.6
3β,5α-THF	100	50	122.6 (10.5)	104.7 (4.9)	97.7 (5.6)	54.5 (1.2)	56.2 (1.9)	56.0 (2.8)	4.0 (2.3)	6.0 (3.3)	5.1 (4.9)	6.6	6.4	7.7	2.3	3.5	7.1
3β,5β-THF	50	20	115.9 (5.6)	104.2 (6.8)	97.3 (5.6)	69.6 (10.8)	68.6 (10.1)	69.5 (9.2)	2.0 (1.6)	5.8 (3.2)	9.7 (2.8)	2.9	3.8	2.0	1.6	3.0	2.6
3α,5α-THF	20	10	120.7 (11.3)	103.5 (8.1)	95.1 (4.5)	85.9 (3.1)	89.7 (1.5)	92.7 (1.1)	3.3 (4.4)	6.7 (3.3)	4.8 (2.0)	6.5	8.0	2.3	4.3	7.4	4.3
3α,5β-THF	20	10	116.6 (11.6)	103.7 (11.6)	93.6 (5.2)	84.9 (2.1)	88.6 (4.9)	96.7 (2.4)	3.3 (4.6)	6.0 (3.2)	5.0 (2.0)	6.5	8.0	2.3	4.8	6.7	4.6
3β,5α-THE	50	50	112.6 (12.9)	106.9 (4.4)	96.9 (5.4)	70.9 (5.8)	70.8 (6.3)	68.1 (4.3)	3.7 (2.7)	3.9 (4.2)	9.6 (5.7)	4.1	6.0	1.0	2.8	4.5	5.2
3α,5α-THE	50	20	120.0 (11.2)	103.1 (7.1)	94.7 (3.8)	88.1 (2.8)	91.7 (2.6)	92.9 (1.3)	3.4 (3.4)	8.2 (4.2)	3.1 (1.6)	4.5	12.7	5.7	3.5	6.5	2.4
3α,5β-THE	50	20	115.6 (13.0)	101.4 (9.2)	91.8 (4.0)	91.1 (4.7)	93.9 (0.6)	97.0 (1.7)	5.1 (2.7)	8.3 (5.2)	7.2 (3.8)	0.7	8.1	5.9	2.8	6.5	5.4
E1-S	20	5	86.3 (9.9)	74.5 (6.1)	66.3 (3.7)	88.0 (1.9)	93.3 (1.0)	95.5 (1.1)	8.4 (4.4)	12.3 (12.3)	10.1 (0.7)	7.3	6.6	8.2	4.0	11.7	9.1
E2-17-S	5	5	67.8 (7.3)	61.4 (5.0)	52.6 (2.1)	68.5 (2.9)	69.7 (5.2)	71.7 (4.7)	3.3 (1.8)	8.7 (6.0)	13.3 (2.9)	8.4	2.7	6.4	2.6	12.5	7.8
E2-3-S	20	5	79.2 (9.6)	70.2 (6.1)	62.8 (3.6)	79.8 (2.0)	82.0 (1.5)	82.9 (1.4)	2.7 (1.6)	1.9 (1.7)	9.0 (3.1)	1.3	3.1	2.1	1.9	2.0	2.8
E3-3-S	200	100	108.4 (12.9)	88.6 (4.3)	84.0 (5.8)	29.7 (3.4)	30.4 (0.1)	33.3 (2.0)	11.4 (1.6)	8.1 (2.8)	9.7 (4.6)	6.9	4.8	14.3	11.5	6.4	6.2
DHEA-S	20	10	90.5 (10.1)	78.9 (6.5)	69.9 (3.3)	90.0 (3.6)	94.7 (0.9)	96.5 (1.7)	8.6 (7.2)	10.0 (9.8)	12.9 (2.0)	6.1	5.4	6.9	11.0	10.6	1.8
E1-G	20	10	92.1 (9.8)	81.8 (4.4)	74.8 (4.6)	79.7 (2.0)	81.9 (3.1)	84.3 (2.7)	7.1 (5.2)	9.0 (5.8)	9.1 (5.1)	1.5	1.3	7.5	8.3	12.8	4.6
E2-3-G	50	10	90.1 (14.1)	72.8 (4.1)	66.1 (2.6)	62.0 (5.1)	65.4 (3.1)	66.3 (4.9)	5.3 (2.0)	6.5 (4.1)	11.8 (3.8)	3.7	2.8	4.6	5.6	7.7	3.4
E2-17-G	50	20	75.3 (10.2)	67.3 (4.8)	59.6 (2.9)	76.2 (0.8)	80.1 (2.0)	79.5 (0.8)	4.2 (0.7)	3.9 (2.4)	11.9 (5.0)	3.1	2.4	4.1	3.8	2.5	4.5
PGE2m	100	50	93.6 (9.1)	86.8 (7.0)	79.2 (3.4)	106.6 (4.7)	105.2 (2.8)	105.6 (3.2)	11.5 (1.9)	4.0 (2.3)	9.3 (4.9)	6.6	2.9	4.7	2.2	2.9	6.1
PGF2αm	50	20	103.3 (7.9)	93.3 (10.7)	82.8 (3.7)	117.7 (6.0)	119.2 (6.4)	120.2 (1.8)	3.4 (2.1)	3.7 (1.0)	9.3 (4.4)	1.7	4.5	3.3	2.1	3.7	4.0
PGD1m	50	20	106.8 (11.2)	91.4 (8.3)	83.1 (3.2)	86.6 (1.3)	96.0 (3.9)	104.0 (2.1)	2.6 (1.8)	8.3 (5.9)	11.5 (1.4)	3.1	4.7	1.5	3.2	7.9	1.3
PGE1m	50	20	102.4 (9.3)	90.8 (6.5)	82.3 (3.7)	121.9 (7.4)	117.3 (3.2)	109.0 (1.8)	4.2 (3.6)	3.3 (1.6)	9.0 (4.1)	1.7	3.3	1.3	5.1	3.3	3.8
PGF1αm	50	10	107.9 (12.6)	95.8 (8.3)	84.2 (3.4)	113.9 (5.9)	113.9 (2.9)	113.6 (3.8)	6.7 (0.5)	4.6 (2.4)	8.2 (5.6)	3.7	4.7	1.7	0.5	5.2	5.8

Table S5. Method validation results for each target using LLE and SPE following LLE determined in charcoal-stripped plasma. LOQs and LODs are presented in pg/mL; recovery, matrix effects are expressed as mean (SD) in %.

Analyte	LOQ (pg/mL)	LOD (pg/mL)	Recovery (%)			Matrix Effect (%)		
			QC_L	QC_M	QC_H	QC_L	QC_M	QC_H
E1	20	10	70.9 (1.2)	65.5 (1.0)	66.1 (0.9)	125.3 (1.9)	113.9 (2.3)	108.5 (1.1)
E2	20	5	70.8 (0.6)	67.9 (1.3)	68.3 (1.2)	129.8 (2.4)	116.1 (2.7)	108.9 (0.4)
17α-E2	20	5	72.0 (0.2)	68.3 (0.8)	68.2 (0.2)	125.9 (0.7)	108.0 (2.4)	106.7 (1.0)
E3	50	20	57.1 (3.8)	55.0 (1.3)	57.6 (1.4)	144.1 (7.6)	125.3 (1.0)	107.4 (0.8)
16β,17α-E3	50	20	71.8 (2.3)	64.5 (0.4)	68.0 (1.5)	133.9 (0.5)	114.1 (0.8)	100.1 (2.3)
16β,17β-E3	20	10	73.7 (0.6)	67.6 (1.1)	69.0 (0.3)	126.5 (1.6)	113.2 (1.9)	107.9 (1.7)
16α,17α-E3	20	10	73.8 (0.4)	68.9 (1.1)	68.3 (0.3)	129.4 (2.9)	113.5 (2.4)	114.5 (0.4)
**P5**	20	10	70.3 (1.0)	77.6 (0.0)	61.8 (1.0)	107.2 (0.7)	86.7 (0.7)	92.3 (2.7)
P4	20	5	70.3 (3.3)	75.4 (1.7)	63.7 (0.8)	88.1 (2.9)	73.4 (0.4)	82.8 (1.5)
17-OHP4	20	5	70.5 (1.8)	79.4 (2.5)	62.9 (2.0)	97.6 (0.6)	84.2 (5.3)	90.8 (1.8)
3α-DHP	50	20	69.9 (3.7)	76.7 (1.3)	63.3 (1.6)	98.8 (2.3)	81.0 (2.2)	84.6 (3.0)
3β-DHP	20	10	70.3 (1.0)	77.7 (0.7)	61.6 (1.1)	106.5 (1.0)	86.4 (0.5)	91.9 (2.7)
5α-DHP	100	50	68.0 (1.7)	77.6 (3.0)	61.9 (6.5)	85.9 (3.6)	70.6 (5.6)	71.8 (2.1)
5β-DHP	100	50	75.1 (15.4)	77.6 (3.0)	61.9 (6.5)	77.8 (14.6)	70.6 (5.6)	71.8 (2.1)
20α-DHP	50	10	65.9 (0.5)	72.0 (2.0)	60.6 (0.9)	59.2 (0.6)	51.9 (2.7)	58.5 (0.3)
20β-DHP	20	5	69.9 (1.7)	76.2 (1.2)	63.4 (1.8)	91.8 (1.3)	77.1 (1.0)	85.6 (0.9)
3β,5α-THP	50	20	67.7 (1.9)	76.3 (1.1)	63.8 (1.9)	97.6 (1.4)	83.5 (1.6)	86.6 (4.5)
3β,5β-THP	100	20	69.2 (1.6)	77.2 (2.7)	62.3 (1.4)	98.1 (1.6)	83.3 (2.1)	87.0 (2.0)
3α,5β-THP	100	50	68.7 (3.4)	78.3 (2.0)	65.7 (3.6)	92.9 (3.6)	81.3 (4.1)	82.5 (3.6)
3α,5α-THP	100	20	68.5 (3.5)	78.1 (1.4)	62.1 (2.3)	89.1 (2.5)	75.0 (2.3)	78.1 (4.0)
DHEA	50	20	69.8 (3.7)	76.1 (1.1)	61.9 (1.0)	104.4 (1.0)	87.4 (3.4)	94.1 (1.4)
5-A5	50	20	71.9 (4.0)	78.8 (0.9)	65.2 (0.2)	95.7 (2.7)	84.2 (1.2)	93.6 (1.6)
4-A5	50	20	72.2 (4.1)	78.9 (1.0)	65.4 (0.3)	95.6 (2.8)	84.3 (1.0)	93.8 (1.7)
A4	50	20	71.9 (1.4)	77.2 (1.3)	63.6 (0.8)	93.4 (2.4)	80.1 (2.4)	87.0 (1.6)
T	20	5	70.3 (1.0)	76.3 (2.0)	65.1 (2.0)	115.5 (3.1)	99.2 (5.7)	106.4 (1.5)
5α-AON	200	50	65.3 (7.0)	72.1 (2.8)	65.0 (2.3)	123.4 (8.9)	93.7 (1.1)	90.8 (2.4)
5β-AON	200	100	86.2 (12.2)	78.0 (4.8)	61.1 (1.7)	99.7 (12.8)	87.0 (3.7)	96.5 (2.3)
5α-DHT	50	20	77.5 (7.0)	78.7 (2.2)	64.4 (0.9)	103.1 (5.8)	87.6 (3.5)	93.3 (1.6)
5β-DHT	100	50	63.7 (1.0)	75.0 (2.6)	62.9 (1.4)	110.5 (6.1)	95.4 (2.9)	94.7 (2.2)
3β,5α-AS	50	50	63.3 (4.1)	74.5 (1.2)	63.6 (0.8)	101.7 (7.5)	85.6 (4.6)	91.7 (2.3)
3β,5β-AS	200	50	72.9 (0.6)	80.1 (2.3)	63.8 (1.8)	108.0 (2.1)	92.4 (1.7)	96.5 (1.9)

3α,5β-AS	100	50	56.0 (0.1)	70.3 (1.2)	64.8 (2.1)	151.0 (8.8)	105.8 (3.7)	98.6 (1.5)
3α,5α-AS	50	5	71.1 (1.6)	75.0 (2.7)	62.7 (1.2)	107.8 (1.7)	93.0 (0.4)	98.3 (1.7)
3β,5α-DIOL	50	50	74.9 (1.4)	78.9 (1.9)	65.6 (2.2)	91.2 (5.6)	81.4 (4.0)	87.8 (3.3)
3β,5β-DIOL	50	50	71.4 (1.0)	81.2 (0.8)	63.8 (0.5)	93.7 (3.8)	83.8 (1.4)	92.8 (2.1)
3α,5β-DIOL	50	50	71.1 (1.5)	79.8 (0.4)	63.6 (2.1)	94.9 (3.2)	89.6 (1.6)	93.5 (1.2)
3α,5α-DIOL	50	50	71.1 (1.5)	79.8 (0.4)	63.6 (2.1)	94.9 (3.2)	89.6 (1.6)	93.5 (1.2)
B	50	20	67.3 (1.7)	77.2 (0.8)	63.3 (0.9)	107.7 (1.3)	96.7 (5.1)	103.0 (1.1)
F	50	10	62.5 (1.0)	73.8 (1.7)	59.0 (2.1)	109.7 (2.2)	97.7 (4.3)	101.6 (4.2)
A	50	20	66.9 (1.3)	73.0 (0.6)	60.3 (1.1)	104.8 (0.3)	93.2 (2.3)	101.4 (1.7)
E	50	10	63.9 (2.0)	73.1 (2.7)	60.4 (1.0)	109.0 (0.4)	98.0 (4.3)	101.7 (4.0)
3β,5α-THF	100	50	64.1 (0.4)	74.6 (1.0)	60.8 (1.7)	109.3 (1.6)	99.7 (4.5)	105.2 (4.6)
3β,5β-THF	50	20	64.2 (1.6)	76.7 (0.8)	60.6 (2.1)	109.7 (2.6)	96.1 (4.9)	101.3 (3.5)
3α,5α-THF	20	10	63.7 (2.1)	75.9 (2.3)	65.7 (2.2)	112.4 (0.2)	97.6 (3.5)	100.9 (4.4)
3α,5β-THF	20	10	63.7 (2.1)	75.9 (2.3)	65.7 (2.2)	112.4 (0.2)	97.6 (3.5)	100.9 (4.4)
3β,5α-THE	50	50	65.5 (1.4)	74.4 (2.1)	60.3 (2.1)	109.5 (1.0)	97.6 (4.1)	103.0 (6.2)
3α,5α-THE	50	20	65.1 (1.5)	75.9 (1.2)	63.6 (2.4)	111.2 (1.7)	97.5 (3.7)	101.7 (3.0)
3α,5β-THE	50	20	61.3 (1.6)	74.5 (1.3)	63.3 (2.7)	114.1 (1.4)	97.9 (2.7)	102.1 (4.2)
E1-S	20	5	74.3 (10.3)	68.4 (1.9)	63.4 (4.3)	75.4 (11.2)	74.8 (16.6)	74.3 (13.6)
E2-17-S	5	5	70.9 (6.0)	63.3 (2.8)	54.4 (3.0)	41.0 (14.9)	42.3 (17.2)	41.7 (14.7)
E2-3-S	20	5	77.9 (6.7)	68.8 (0.6)	64.8 (2.2)	46.3 (18.3)	46.8 (19.5)	48.2 (17.4)
E3-3-S	200	100	92.4 (5.8)	83.5 (4.2)	81.2 (1.6)	9.9 (5.8)	11.5 (7.6)	12.9 (7.8)
DHEA-S	20	10	85.0 (10.0)	77.5 (0.7)	73.3 (3.9)	88.9 (3.2)	87.7 (8.5)	87.6 (4.5)
E1-G	20	10	91.5 (10.8)	85.2 (1.9)	77.9 (5.7)	90.4 (2.4)	86.1 (2.5)	82.6 (1.5)
E2-3-G	50	10	82.7 (7.0)	75.6 (0.8)	69.3 (2.8)	64.4 (5.6)	61.1 (7.8)	58.1 (7.4)
E2-17-G	50	20	81.0 (10.5)	73.5 (2.7)	66.8 (3.6)	82.2 (1.0)	78.3 (5.7)	77.0 (4.9)
PGE2m	100	50	NA	69.0 (3.5)	48.5 (0.5)	NA	92.6 (11.6)	86.0 (23.7)
PGF2αm	50	20	NA	102.0 (9.4)	86.2 (4.2)	NA	132.7 (15.3)	116.6 (11.6)
PGD1m	50	20	86.2 (8.1)	77.9 (1.7)	70.3 (4.5)	73.3 (16.6)	87.6 (13.5)	88.0 (15.0)
PGE1m	50	20	NA	87.8 (2.0)	74.4 (2.1)	NA	100.7 (9.2)	87.7 (22.1)
PGF1αm	50	10	101.1 (3.9)	96.1 (3.2)	84.8 (2.8)	92.7 (3.5)	115.3 (7.8)	100.0 (3.0)

Table S6. Comparison of the overall performance of the current workflow with state-of-the-art methods.

Matrix and Sample Volume	Coverage						Target Number	Sensitivity (LOQ range, pg/mL)	Runtime (min/target)	Reference
	Estrogens	Glucocorticoids	Androgens	Progestogens	Steroid Conjugates	PG Metabolites				
Plasma, 500 μL		✓		✓			23	50−500	0.65	[1]
Plasma, 500 μL	✓	✓	✓	✓			17	0.91−701.5	0.53	[2]
Plasma, 40 μL	✓	✓	✓	✓			38	50−50000	0.52	[3]
Serum, 500 μL		✓	✓	✓			17	30.4-2302	1	[4]
Serum, 200 μL		✓	✓	✓			23	100−1000	0.26	[5]
Serum, 100 μL	✓	✓	✓	✓			12	5−1000	1.75	[6]
Urine, 200 μL	✓	✓	✓	✓	✓	✓	56	1000−200000	0.22	[7]
Plasma, 250 μL	✓	✓	✓	✓	✓	✓	61	5−200	0.48	**Current Workflow**

Table S7. Results of the dilution experiment using an authentic pregnancy cattle plasma sample diluted with charcoal-stripped plasma (1:1). Samples were prepared and analyzed in triplicate. LOQs and LODs are presented in pg/mL; concentrations are reported as mean in pg/mL; accuracy are reported as mean absolute relative error (SD) in % based on dilution-corrected back-calculated concentrations relative to those in the original sample. [a] Concentrations are reported by valley-split quantification. [b] Concentrations are reported by composite quantification.

Analyte	ISTD	LOQ (pg/mL)	LOD (pg/mL)	Concentration (pg/mL)	Accuracy (%)
E1	E1-d4	20	10	684.4	2.0 (1.1)
E2	E2-d5	20	5	94.3	5.0 (1.7)
17α-E2	E2-d5	20	5	99.5	1.9 (0.9)
E3[a]	E2-d5	50	20	ND	NA
16β,17α-E3[a]	E2-d5	50	20	ND	NA
16β,17β-E3	E2-d5	20	10	15.4	58.0 (3.6)
16α,17α-E3	E2-d5	20	10	14.8	22.0 (9.8)
P4	P4-d9	20	5	177.1	8.4 (2.9)
17-OHP4	17-OHP4-d8	20	5	183	6.4 (5.2)
P5 + 3β-DHP[b]	P5-d4	20	10	265.2	5.3 (1.8)
5α-DHP[a]	P5-d4	100	50	ND	NA
5β-DHP[a]	P5-d4	100	50	ND	NA
20α-DHP	17-OHP4-d8	50	10	96.4	9.5 (5.9)
20β-DHP	17-OHP4-d8	20	5	99.7	10.3 (6.5)
3β,5α-THP	P5-d4	50	20	140.3	5.8 (3.5)
3β,5β-THP	P5-d4	100	20	112.1	4.1 (0.5)
3α,5β-THP	P5-d4	100	50	225.3	5.7 (4.0)
3α,5α-THP	P5-d4	100	20	270	8.7 (4.4)
DHEA	A4-d7	50	20	418.9	10.0 (2.7)
4-A5 + 5-A5[b]	T-d3	50	20	ND	NA
A4	A4-d7	50	20	43.1	19.4 (5.3)
T	T-d3	20	5	38.1	10.9 (8.7)
5α-AON	AS-d2	200	50	ND	NA
5β-AON	AS-d2	200	100	1279.89	11.8 (2.6)
3β,5β-AS + 5α-DHT[b]	AS-d2	50	20	ND	NA
5β-DHT	AS-d2	100	50	ND	NA
3β,5α-AS	AS-d2	50	50	ND	NA
3α,5β-AS	AS-d2	100	50	ND	NA
3α,5α-AS	AS-d2	50	5	4233.2	4.0 (0.9)
3β,5α-DIOL	AS-d2	50	50	ND	NA

3β,5β-DIOL	AS-d2	50	50	ND	NA
3α,5β-DIOL	AS-d2	50	50	1128.5	9.6 (2.5)
3α,5α-DIOL	AS-d2	50	50	ND	NA
B	F-d4	50	20	55.6	11.3 (6.9)
F	F-d4	50	10	2754.6	4.2 (2.8)
A	F-d4	50	20	105.8	6.3 (3.0)
E	F-d4	50	10	1080.3	5.1 (2.5)
3β,5α-THF	F-d4	100	50	ND	NA
3β,5β-THF	F-d4	50	20	137.3	6.2 (2.2)
3α,5α-THF[a]	F-d4	20	10	281.3	3.8 (1.4)
3α,5β-THF[a]	F-d4	20	10	1670	5.3 (3.9)
3β,5α-THE	F-d4	50	50	57.9	6.6 (3.1)
3α,5α-THE	F-d4	50	20	120.3	9.9 (4.2)
3α,5β-THE	F-d4	50	20	160.3	12.8 (2.8)
E1-S	E2-3-S-d4	20	5	8223.6	4.9 (2.5)
E2-17-S	E2-3-S-d4	5	5	ND	NA
E2-3-S	E2-3-S-d4	20	5	75	4.8 (2.5)
E3-3-S	E2-3-S-d4	200	100	ND	NA
DHEA-S	E2-3-S-d4	20	10	231.6	6.7 (3.0)
E1-G	E2-3-G-d3	20	10	3819	4.4 (3.0)
E2-3-G	E2-3-G-d3	50	10	101.7	3.5 (3.0)
E2-17-G	E2-3-G-d3	50	20	ND	NA
PGE2m	PGE1m-d4	100	50	76.6	13.1 (9.1)
PGF2αm	PGF2αm-d4	50	20	431.1	4.7 (2.5)
PGD1m	PGE1m-d4	50	20	ND	NA
PGE1m	PGE1m-d4	50	20	268	5.2 (1.8)
PGF1αm	PGF2αm-d4	50	10	152.5	6.7 (2.8)

Table S8. Trueness assessment results using standard reference material (SRM) 1950 human plasma. Samples were prepared and analyzed in triplicate. Concentrations are presented as mean (SD) in ng/mL. Trueness was reported as absolute relative error in % calculated by comparing the mean measured concentrations with the corresponding mean values assigned by National Institute of Standards and Technology (NIST). [a] Certified value. [b] The cortisol (F) value was reclassified from certified to non-certified by NIST in 2023.

Analyte	NIST-assigned Concentration (ng/mL)	Measured Concentration (ng/mL)	Trueness (%)
P4	1.482 (0.038)[a]	1.432 (0.049)	3.4
T	2.214 (0.047)[a]	1.975 (0.018)	10.8
F	83.9 (1.7)[b]	79.4 (0.8)	5.4

Table S9. Long-sequence response of representative ISTDs across consecutive measurements of three 96-well plates. Target responses are summarized as non-normalized areas, reported as overall CVs and plate-specific mean (SD) values.

ISTD	Category	Polarity Switch Segment	Overall CV (%)	Plate 1 Peak Area	Plate 2 Peak Area	Plate 3 Peak Area
F-d4	Glucocorticoid	(−)-ESI	26.1	100593.3 (24239.8)	149037.6 (12381.3)	103804.3 (25900.3)
E2-3-S-d4	Steroid Conjugate	(−)-ESI	23.1	174161.7 (39323.1)	233168.7 (23258.3)	175315.6 (42345.6)
E2-3-G-d3	Steroid Conjugate	(−)-ESI	25.3	102370.4 (23802.8)	137444.0 (13253.6)	91996.6 (23331.9)
T-d3	Androgen	(+)-ESI	11.6	2259662.8 (150980.6)	2094234.9 (258859.2)	1933721.9 (192745.0)
PGF2αm-d4	PG Metabolite	(+)-ESI	14.2	733502.6 (80799.8)	803531.5 (77407.8)	836823.8 (146709.8)
17-OHP4-d8	Progestogen	(+)-ESI	10.3	1751900.7 (125512.9)	1862769.2 (162584.7)	1613755.2 (163291.0)

Table S10. Long-sequence response of representative targets across consecutive measurements of three 96-well plates. Target responses are summarized as target-to-ISTD-normalized areas for orderly interspersed QC_L, QC_M, and QC_H samples, reported as overall CVs and plate-specific mean (SD) values. RT drift values summarize chromatographic variability across and within plates.

Target	Category	Polarity Switch Segment	QC Level	Overall CV (%)	Plate 1 Peak Area	Plate 2 Peak Area	Plate 3 Peak Area	Total RT Drift (min)	Plate 1 RT Drift (min)	Plate 2 RT Drift (min)	Plate 3 RT Drift (min)
B	Glucocorticoid	(−)-ESI	QC_L	9.7	0.8 (0.0)	0.9 (0.0)	0.8 (0.1)	0.03	0	0.01	0
			QC_M	7.2	3.2 (0.1)	3.3 (0.1)	3.3 (0.5)	0.05	0.01	0.02	0
			QC_H	7.9	21.4 (1.2)	24.3 (0.5)	23.0 (2.4)	0.04	0	0.01	0.01
E2-3-S	Steroid Conjugate	(−)-ESI	QC_L	4.5	0.6 (0.0)	0.6 (0.0)	0.6 (0.0)	0.27	0.05	0	0.11
			QC_M	5.8	2.4 (0.0)	2.1 (0.0)	2.4 (0.1)	0.29	0.07	0.02	0.12
			QC_H	2	14.9 (0.0)	14.6 (0.1)	15.2 (0.3)	0.26	0.06	0.02	0.13
E1-G	Steroid Conjugate	(−)-ESI	QC_L	4.5	0.2 (0.0)	0.2 (0.0)	0.2 (0.0)	0.05	0	0	0.02
			QC_M	9.7	0.9 (0.0)	0.8 (0.0)	1.0 (0.0)	0.05	0.01	0	0.01
			QC_H	3.9	6.4 (0.1)	5.9 (0.1)	6.3 (0.1)	0.04	0.01	0	0
T	Androgen	(+)-ESI	QC_L	2.7	0.2 (0.0)	0.2 (0.0)	0.2 (0.0)	0.03	0	0	0.01
			QC_M	6.2	1.0 (0.0)	0.9 (0.0)	1.0 (0.0)	0.06	0.01	0.02	0.01
			QC_H	2.4	6.2 (0.2)	6.1 (0.0)	6.4 (0.0)	0.05	0	0.02	0.01
PGF2αm	PG Metabolite	(+)-ESI	QC_L	3.1	0.3 (0.0)	0.3 (0.0)	0.2 (0.0)	0.03	0.01	0	0.01
			QC_M	4.9	1.0 (0.0)	0.9 (0.0)	1.0 (0.0)	0.04	0.01	0.01	0
			QC_H	2.6	6.3 (0.0)	6.2 (0.1)	6.5 (0.2)	0.04	0	0.01	0.01
17-OHP4	Progestogen	(+)-ESI	QC_L	3.7	0.2 (0.0)	0.2 (0.0)	0.2 (0.0)	0.04	0	0	0.02
			QC_M	5.3	1.0 (0.0)	0.9 (0.0)	1.0 (0.0)	0.06	0.02	0.01	0.01
			QC_H	2.3	6.2 (0.1)	6.1 (0.0)	6.4 (0.1)	0.05	0	0.02	0.01

Table S11. Quantitative profiles of the steroidome and PG metabolites in cattle plasma samples. Each sample was prepared and analyzed independently in triplicate (R1, R2 and R3). Concentrations are reported in pg/mL. FP, follicular phase; LP, luteal phase. [a] Concentrations are reported by valley-split quantification. [b] Concentrations are reported by composite quantification.

Target	FP_R1	FP_R2	FP_R3	LP_R1	LP_R2	LP_R3	Pregnancy_R1	Pregnancy_R2	Pregnancy_R3	Parturition_R1	Parturition_R2	Parturition_R3	Male_R1	Male_R2	Male_R3
E1	ND	ND	ND	ND	ND	ND	26.5	30.3	29.2	2959.6	3003.5	2975.3	ND	ND	ND
E2	ND	ND	ND	ND	ND	ND	ND	ND	ND	469.7	471.1	476.8	ND	ND	ND
17α-E2	ND	ND	ND	ND	ND	ND	ND	ND	ND	212.5	211.3	211.8	ND	ND	ND
E3[a]	ND	ND	ND	ND	ND	ND	ND	ND	ND	ND	ND	ND	ND	ND	ND
16β,17α-E3[a]	ND	ND	ND	ND	ND	ND	ND	ND	ND	ND	ND	ND	ND	ND	ND
16β,17β-E3	ND	ND	ND	ND	ND	ND	ND	ND	ND	ND	ND	ND	ND	ND	ND
16α,17α-E3	ND	ND	ND	ND	ND	ND	ND	ND	ND	ND	ND	ND	ND	ND	ND
P5 + 3β-DHP[b]	229.1	204.6	190.4	223.4	194.1	184.2	591.4	534.1	518.1	446.0	361.9	355.8	73.3	77.4	60.4
P4	68.1	73.6	71.0	3810.7	3711.8	3598.9	6319.9	6771.0	6648.2	166.0	168.2	164.6	22.8	22.2	21.7
17-OHP4	10.4	10.3	7.4	254.9	249.4	248.3	1110.1	1032.0	1057.3	256.5	230.9	243.4	25.4	26.8	21.1
3α-DHP	ND	ND	ND	103.2	134.5	121.1	184.8	222.0	212.8	ND	ND	ND	ND	ND	ND
5α-DHP[a]	ND	ND	ND	ND	ND	ND	ND	ND	ND	ND	ND	ND	ND	ND	ND
5β-DHP[a]	ND	ND	ND	ND	ND	ND	ND	ND	ND	ND	ND	ND	ND	ND	ND
20α-DHP	15.6	17.2	16.0	109.3	121.9	121.9	151.6	124.7	136.8	238.4	235.7	226.4	16.8	16.3	19.5
20β-DHP	27.7	23.6	24.5	329.7	325.5	296.4	1203.1	1079.7	1172.0	94.6	95.2	96.1	7.8	9.6	14.3
3β,5α-THP	ND	ND	ND	292.4	345.9	333.1	512.5	553.5	496.7	69.0	85.5	64.0	ND	ND	ND
3β,5β-THP	ND	ND	ND	126.5	115.3	88.2	573.2	629.5	545.4	144.2	156.9	126.0	ND	ND	ND
3α,5β-THP	ND	ND	ND	279.7	354.6	280.9	989.9	1120.5	977.9	177.9	179.5	181.7	ND	ND	ND
3α,5α-THP	59.6	68.1	55.1	783.8	761.0	686.8	1275.0	1694.0	1587.2	384.2	362.4	353.7	ND	ND	ND
DHEA	714.5	638.3	728.1	162.2	ND	179.1	1071.5	1133.5	851.2	2253.3	2303.2	2237.6	365.6	435.5	348.7
4-A5 + 5-A5[b]	ND	ND	ND	ND	ND	ND	ND	ND	ND	ND	ND	ND	ND	ND	ND
A4	ND	ND	ND	ND	ND	ND	62.2	70.9	57.3	269.6	286.1	285.4	14.2	10.4	12.2
T	13.3	13.4	15.0	ND	ND	ND	36.3	38.2	38.6	134.8	135.6	141.0	493.5	509.6	510.1
5α-AON	ND	ND	ND	ND	ND	ND	ND	ND	ND	ND	ND	ND	ND	ND	ND
5β-AON	226.8	264.9	236.7	678.4	771.3	678.0	370.9	471.4	429.7	792.9	823.7	875.4	440.1	430.2	334.3
5β-DHT	ND	ND	ND	ND	ND	ND	252.7	187.0	198.3	ND	ND	ND	ND	ND	ND
3β,5β-AS + 5α-DHT[b]	ND	ND	ND	ND	ND	ND	ND	ND	ND	ND	ND	ND	ND	ND	ND
3β,5α-AS	ND	ND	ND	ND	ND	ND	ND	ND	ND	ND	ND	ND	ND	ND	ND
3α,5β-AS	ND	ND	ND	ND	ND	ND	ND	ND	ND	ND	ND	ND	ND	ND	ND
3α,5α-AS	ND	ND	ND	ND	ND	ND	181.8	212.6	196.7	1092.8	1195.1	951.3	ND	ND	ND

3β,5α-DIOL	401.9	365.2	373.1	606.5	556.5	554.0	1406.8	1432.3	1498.1	706.5	618.7	752.9	1266.8	1306.2	1043.9
3β,5β-DIOL	118.7	115.6	96.9	215.8	208.7	201.1	258.0	336.9	306.7	150.5	175.9	153.4	199.3	256.6	232.3
3α,5β-DIOL	ND	ND	ND	ND	ND	ND	ND	ND	ND	ND	ND	ND	ND	ND	ND
3α,5α-DIOL	ND	ND	ND	ND	ND	ND	ND	ND	ND	ND	ND	ND	ND	ND	ND
B	532.1	512.3	516.0	81.6	77.6	83.9	2344.5	2199.1	2347.1	1131.5	1219.8	1109.8	1050.1	1092.6	1052.0
F	1993.0	2029.0	2066.2	6179.0	6270.9	6206.4	52686.4	50777.8	55160.6	23553.0	24446.0	23011.1	10175.0	10248.8	10101.6
A	499.0	473.1	433.5	129.8	128.0	119.3	1488.0	1649.0	1675.2	476.4	514.5	460.9	328.2	343.7	323.9
E	556.6	558.7	565.2	1514.1	1569.1	1545.2	6389.3	6209.3	6534.7	4726.3	5240.6	4984.9	628.2	633.2	617.2
3β,5α-THF	167.3	208.6	215.2	100.4	121.5	86.9	650.6	633.7	503.9	129.0	140.6	147.8	100.5	121.9	86.4
3β,5β-THF	81.6	91.1	87.5	181.7	192.7	200.0	1148.0	1119.9	1091.6	677.5	700.5	662.8	53.8	69.1	68.9
3α,5α-THF[a]	559.9	563.7	562.9	1390.5	1336.7	1420.3	3964.1	3807.6	3835.8	4947.5	5168.2	4886.8	456.1	448.2	454.0
3α,5β-THF[a]	579.4	570.1	565.2	1424.0	1363.3	1424.9	3892.1	3640.2	3702.0	4958.7	5152.9	4901.2	457.5	462.5	454.3
3β,5α-THE	ND	ND	ND	ND	ND	ND	995.9	1032.8	899.1	94.4	103.3	102.6	ND	ND	ND
3α,5α-THE	36.9	41.8	35.8	84.8	83.6	92.0	1119.4	987.5	947.7	617.9	637.0	617.6	ND	ND	ND
3α,5β-THE	73.1	59.5	56.3	221.2	198.7	214.9	581.3	740.7	605.8	623.9	630.2	615.5	71.5	70.3	70.5
E1-S	25.5	33.0	34.1	20.9	23.2	24.1	1227.3	1169.1	1216.4	19403.6	20593.6	20016.9	90.5	76.8	86.5
E2-17-S	ND	ND	ND	ND	ND	ND	8.0	10.6	7.7	ND	ND	ND	ND	ND	ND
E2-3-S	ND	ND	ND	ND	ND	ND	24.4	24.2	21.8	374.3	357.2	362.2	ND	ND	ND
E3-3-S	ND	ND	ND	ND	ND	ND	ND	ND	ND	ND	ND	ND	ND	ND	ND
DHEA-S	915.8	971.8	957.3	442.1	412.3	442.0	495.2	541.9	537.6	2136.3	2615.5	2436.2	4574.7	4419.6	4308.0
E1-G	ND	ND	ND	ND	ND	ND	227.5	230.2	290.0	4053.9	4097.4	3743.3	ND	ND	ND
E2-3-G	ND	ND	ND	ND	ND	ND	ND	ND	ND	ND	ND	ND	ND	ND	ND
E2-17-G	ND	ND	ND	ND	ND	ND	ND	ND	ND	ND	ND	ND	ND	ND	ND
PGE2m	ND	ND	ND	ND	ND	ND	ND	ND	ND	327.3	329.4	356.1	ND	50.1	ND
PGF2αm	45.6	33.9	40.3	78.6	70.8	77.0	150.5	105.8	124.8	2829.2	2824.8	2888.3	71.2	56.2	50.8
PGD1m	ND	ND	ND	ND	ND	ND	ND	ND	ND	ND	ND	ND	ND	ND	ND
PGE1m	ND	ND	ND	ND	ND	ND	ND	ND	ND	1693.9	1692.0	1704.3	130.1	135.5	165.4
PGF1αm	ND	ND	ND	ND	ND	ND	ND	ND	ND	581.3	599.5	582.9	ND	ND	ND

Table S12. Quantitative profiles of the steroidome and PG metabolites in human plasma samples. Numbers following each sampling phase denote individual sampling time points. Concentrations are reported in pg/mL. FP, follicular phase; OV, ovulation; LP, luteal phase. [a] Concentrations are reported by valley-split quantification. [b] Concentrations are reported by composite quantification.

Target	FP_1	FP_2	FP_3	OV_1	OV_2	OV_3	OV_4	OV_5	LP_1	LP_2	LP_3	LP_4	Pregnancy_1	Pregnancy_2	Pregnancy_3	Male_1	Male_2	Male_3
E1	43.3	38.7	46.2	106.8	109.5	71.5	104.1	63.6	70.9	68.3	70.8	51.4	12495.8	2642.8	2021.3	26.9	28.0	26.8
E2	64.9	56.9	80.5	223.1	199.0	53.5	149.5	72.0	136.7	107.9	148.0	84.2	21133.0	6767.4	5238.6	ND	ND	ND
17α-E2	ND	ND	ND	ND	ND	ND	ND	ND	ND	ND	ND	ND	ND	ND	ND	ND	ND	ND
E3[a]	ND	ND	ND	ND	ND	ND	ND	ND	ND	ND	ND	ND	7481.4	1725.7	1159.9	ND	ND	ND
16β,17α-E3[a]	ND	ND	ND	ND	ND	ND	ND	ND	ND	ND	ND	ND	ND	ND	ND	ND	ND	ND
16β,17β-E3	ND	ND	ND	ND	ND	ND	ND	ND	ND	ND	ND	ND	ND	ND	ND	ND	ND	ND
16α,17α-E3	ND	ND	ND	ND	ND	ND	ND	ND	ND	ND	ND	ND	ND	ND	ND	ND	ND	ND
P5 + 3β-DHP[b]	619.3	499.9	487.1	750.9	1196.3	1025.9	646.8	452.5	743.8	1546.5	273.2	814.2	443.4	451.7	328.9	405.7	317.0	342.4
P4	126.5	102.7	63.6	101.7	515.2	596.8	1414.3	1563.5	6450.5	6839.1	8156.3	9202.3	84546.7	41378.4	33418.2	37.0	33.6	24.4
17-OHP4	404.2	336.2	461.1	558.6	1253.2	873.3	1289.4	891.9	1425.0	1713.0	1061.2	1397.2	16475.3	3819.3	2998.7	789.2	754.3	505.6
3α-DHP	ND	ND	ND	ND	ND	ND	ND	ND	29.7	94.2	60.3	109.9	1482.2	995.0	706.9	ND	ND	ND
5α-DHP[a]	ND	ND	ND	ND	ND	ND	ND	ND	ND	ND	ND	ND	ND	ND	ND	ND	ND	ND
5β-DHP[a]	97.7	83.1	ND	67.6	109.1	99.9	371.5	627.4	559.2	845.0	611.3	882.4	36084.6	9325.1	7437.0	50.9	ND	87.6
20α-DHP	129.2	112.6	60.3	73.3	208.7	231.1	277.6	355.7	1449.3	2315.1	2557.5	2726.5	20540.6	11785.0	9501.5	50.9	46.6	45.0
20β-DHP	ND	ND	ND	ND	ND	ND	ND	ND	23.6	13.1	25.1	17.5	76.8	29.8	24.0	ND	ND	ND
3β,5α-THP	ND	ND	ND	ND	ND	ND	ND	ND	130.1	323.7	129.5	346.7	3453.3	1607.0	1336.3	ND	ND	ND
3β,5β-THP	ND	ND	ND	ND	ND	ND	ND	ND	ND	ND	ND	31.1	385.4	266.3	194.9	ND	ND	ND
3α,5β-THP	ND	ND	ND	ND	ND	ND	ND	ND	ND	114.9	ND	114.8	4091.2	2803.7	2317.3	ND	ND	ND
3α,5α-THP	76.2	81.7	50.8	72.7	88.4	75.5	108.3	114.7	308.1	549.2	489.0	625.3	13334.6	5501.3	4506.7	53.4	53.2	52.3
DHEA	8322.5	6571.7	7852.9	6658.7	9664.4	8072.0	5073.9	2678.0	3752.6	8974.2	2255.9	8383.9	1945.3	2277.4	1666.1	4928.2	4610.9	4657.8
4-A5 + 5-A5[b]	651.1	565.6	471.8	541.2	614.3	509.2	594.0	462.6	465.7	471.1	391.9	451.5	674.3	407.4	302.0	1266.0	1218.4	864.8
A4	1117.3	906.9	994.4	1205.7	1473.7	1132.5	929.8	655.8	721.6	1238.1	554.5	1005.7	9902.8	6445.7	4616.8	768.4	711.1	762.6
T	215.8	167.1	208.6	233.4	243.2	194.3	322.7	246.3	228.3	246.6	169.6	185.9	3859.6	2255.9	1750.3	8829.6	11555.2	3152.2
5α-AON	332.8	243.8	ND	ND	101.5	115.4	ND	ND	ND	ND	ND	257.1	597.9	515.4	361.7	ND	ND	149.2
5β-AON	ND	ND	ND	ND	ND	ND	ND	ND	ND	ND	ND	ND	ND	ND	ND	ND	ND	ND
5β-DHT	ND	ND	ND	ND	ND	ND	ND	ND	ND	ND	ND	ND	ND	ND	ND	ND	ND	ND
3β,5β-AS + 5α-DHT[b]	169.8	145.0	102.7	159.1	151.8	114.1	139.6	130.3	94.8	116.1	105.8	104.0	467.8	384.8	278.0	266.9	266.6	314.1
3β,5α-AS	ND	ND	ND	ND	ND	ND	ND	ND	ND	ND	ND	ND	146.8	174.3	161.6	ND	ND	ND
3α,5β-AS	65.9	60.9	ND	ND	64.4	ND	ND	ND	ND	102.8	ND	62.6	181.2	164.7	120.9	ND	ND	ND
3α,5α-AS	319.3	236.4	235.9	293.7	292.1	219.9	285.6	235.2	259.9	251.9	208.4	233.5	1439.7	964.1	735.4	283.5	280.2	363.1

3β,5α-DIOL	ND	ND	ND	ND	ND	ND	ND	ND	ND	ND	ND	ND	16041.9	2653.9	2080.6	ND	ND	ND
3β,5β-DIOL	ND	ND	ND	ND	ND	ND	ND	ND	ND	ND	ND	ND	ND	ND	ND	ND	ND	ND
3α,5β-DIOL	ND	ND	ND	ND	ND	ND	ND	ND	ND	ND	ND	ND	ND	ND	ND	64.4	60.0	425.6
3α,5α-DIOL	ND	ND	ND	ND	ND	ND	63.0	ND	ND	ND	ND	ND	117.9	172.3	126.5	ND	ND	275.9
B	4276.2	3363.2	5759.7	5489.2	14027.8	11159.4	3278.8	3361.3	3507.7	14979.5	3104.6	8510.2	2983.1	1969.2	1690.0	379.8	374.5	417.7
F	90317.9	74879.6	70493.3	84844.5	121846.2	86934.7	53587.9	47035.3	57065.1	94820.9	38210.9	79632.6	121222.5	93394.1	77484.2	34963.4	34754.2	36732.2
A	1248.5	925.7	954.4	1506.3	2234.1	1535.7	1159.0	992.9	2060.4	1730.1	888.2	959.6	1494.1	989.0	866.1	ND	ND	293.9
E	18863.6	15098.3	12208.1	16403.5	19740.3	14476.6	17667.0	16726.6	17010.6	15881.6	12812.7	13039.0	37344.0	25784.0	21930.8	12385.6	12293.5	12224.7
3β,5α-THF	988.6	713.4	541.2	553.7	596.9	462.5	752.1	861.6	999.4	400.9	718.3	700.3	206.6	313.9	237.8	812.3	891.4	573.2
3β,5β-THF	ND	ND	ND	ND	ND	ND	ND	ND	ND	ND	ND	ND	ND	ND	ND	21.4	25.9	ND
3α,5α-THF[a]	3496.8	2815.0	2056.8	2928.0	3103.3	2134.6	2271.5	3023.2	2674.5	1715.4	2015.1	2619.5	289.4	436.1	402.1	4159.6	3878.2	2523.6
3α,5β-THF[a]	3264.4	2420.6	1890.8	2420.1	2949.7	1985.8	1665.9	2638.8	1720.4	1223.6	1579.1	1914.9	193.8	358.2	267.9	1809.0	1948.2	1877.8
3β,5α-THE	100.3	97.5	85.3	62.6	58.2	63.4	449.1	637.3	389.3	90.8	451.7	108.1	461.7	67.1	84.0	95.7	108.9	120.0
3α,5α-THE	433.0	353.2	288.6	231.3	221.7	276.5	644.7	739.5	634.6	227.4	613.2	413.5	173.7	206.2	171.0	430.1	531.5	250.4
3α,5β-THE	649.9	518.0	393.7	679.7	774.0	525.7	433.0	597.8	450.0	533.7	408.0	547.0	724.6	643.2	500.9	326.4	313.2	1087.0
E1-S	1684.8	1324.0	1995.1	5347.5	5956.5	3128.5	5298.4	2124.2	3238.6	2591.6	3445.4	2371.8	123043.9	33052.8	29336.3	729.9	677.9	1264.8
E2-17-S	ND	ND	9.3	28.9	ND	11.2	ND	ND	10.0	ND	ND	ND	758.5	155.4	130.6	ND	ND	ND
E2-3-S	ND	ND	ND	52.5	27.6	7.3	11.2	5.3	11.3	14.9	16.5	ND	1287.8	464.8	351.2	ND	ND	ND
E3-3-S	ND	ND	ND	ND	ND	ND	ND	ND	ND	ND	ND	ND	22932.7	10318.9	6362.7	ND	ND	ND
DHEA-S	416117.9	350312.7	310644.6	412648.4	452338.8	364430.7	440773.9	378754.1	465278.1	404562.2	381456.5	373405.7	171632.9	207007.1	189484.8	600715.6	594442.8	701092.8
E1-G	44.6	75.4	176.9	515.8	830.7	412.6	347.3	201.3	192.8	406.7	470.2	264.3	21150.5	3349.8	2775.3	177.4	ND	ND
E2-3-G	32.3	27.5	38.4	58.8	108.3	69.9	ND	ND	ND	68.9	ND	52.6	1043.4	271.3	154.1	ND	ND	ND
E2-17-G	ND	ND	ND	ND	ND	ND	ND	ND	ND	ND	ND	ND	116.9	113.1	84.7	ND	ND	ND
PGE2m	ND	ND	ND	ND	ND	ND	ND	ND	ND	ND	ND	ND	ND	ND	ND	ND	ND	ND
PGF2αm	ND	ND	ND	ND	ND	ND	ND	ND	ND	ND	ND	ND	ND	ND	ND	ND	ND	ND
PGD1m	ND	ND	ND	ND	ND	ND	ND	ND	ND	ND	ND	ND	ND	ND	ND	ND	ND	ND
PGE1m	ND	ND	ND	ND	ND	ND	ND	ND	ND	ND	ND	ND	ND	ND	ND	ND	ND	ND
PGF1αm	ND	ND	ND	ND	ND	ND	ND	ND	ND	ND	ND	ND	ND	ND	ND	ND	ND	ND

Table S13. Comparison of target concentrations measured in human plasma samples with available reference intervals (RIs). Concentrations are presented as observed ranges (minimum–maximum). Interpretation indicates whether the measured range was within the corresponding RI or overlapped it while extending beyond its upper or lower limit. FP, follicular phase; OV, ovulation; LP, luteal phase; IEA, immunoenzymatic assay; ECLIA, electrochemiluminescence immunoassay. RIs attributed to ARUP Laboratories and Mayo Clinic Laboratories were obtained from their online test directories.

Target	Concentration Range of this Study (pg/mL)	Sampling Phase of this study	RI (pg/mL)	Reference Population / Physiological Stage	Matrix	Method	Reference	Interpretation
E1	26.8–28	Male	9–36	Male	serum/plasma	LC–MS/MS	ARUP Laboratories	Within RI
	38.7–46.2	FP	<150	Female (early FP)	serum/plasma	LC–MS/MS	ARUP Laboratories	Within RI
	63.6–109.5	OV	100–250	Female (late FP)	serum/plasma	LC–MS/MS	ARUP Laboratories	Partially below RI
	51.4–70.9	LP	<200	Female (LP)	serum/plasma	LC–MS/MS	ARUP Laboratories	Within RI
	2021.3–12495.8	Pregnancy	488–13478	Pregnancy (34–39 weeks)	serum	LC–MS/MS	[8]	Within RI
E2	ND	Male	10–42	Male	serum/plasma	LC–MS/MS	ARUP Laboratories	Not assessable
	56.9–80.5	FP	30–100	Female (early FP)	serum/plasma	LC–MS/MS	ARUP Laboratories	Within RI
	53.5–223.1	OV	100–400	Female (late FP)	serum/plasma	LC–MS/MS	ARUP Laboratories	Partially below RI
	84.2–148	LP	50–150	Female (LP)	serum/plasma	LC–MS/MS	ARUP Laboratories	Within RI
	5238.6–21133.0	Pregnancy	2014–12778	Pregnancy (34–39 weeks)	serum	LC–MS/MS	[8]	Partially above RI
E3	ND	Male	<70	Male	serum	IEA	Mayo Clinic Laboratories	Not assessable
	ND	FP, OV, LP	<80	Female	serum	IEA	Mayo Clinic Laboratories	Not assessable
	1159.9–7481.4	Pregnancy	1025–4984	Pregnancy (34–39 weeks)	serum	LC–MS/MS	[8]	Partially above RI
P5	317.0–405.7	Male	230–1730	Male	serum/plasma	LC–MS/MS	ARUP Laboratories	Within RI
	273.2–1546.5	FP, OV, LP	150–1320	Female	serum/plasma	LC–MS/MS	ARUP Laboratories	Partially above RI
P4	24.4–37.0	Male	<200	Male	serum	ECLIA	Mayo Clinic Laboratories	Within RI
	63.6–126.5	FP	<=890	Female (FP)	serum	ECLIA	Mayo Clinic Laboratories	Within RI
	101.7–1563.5	OV	<=1200	Female (OV)	serum	ECLIA	Mayo Clinic Laboratories	Partially above RI
	6450.5–9202.3	LP	1800–24000	Female (LP)	serum	ECLIA	Mayo Clinic Laboratories	Within RI
	33418.2–84546.7	Pregnancy	25000–83000	Pregnancy (3rd trimester)	serum	ECLIA	Mayo Clinic Laboratories	Partially above RI
17–OHP4	505.6–789.2	Male	<1390	Male	serum/plasma	LC–MS/MS	ARUP Laboratories	Within RI
	336.2–1713.0	FP, OV, LP	<2070	Female	serum/plasma	LC–MS/MS	ARUP Laboratories	Within RI
	2998.7–16475.3	Pregnancy	3170–14510	Pregnancy (40 weeks)	serum	LC–MS/MS	[9]	Partially outside RI
DHEA	1666.1–9664.4	All Phases	<13000	Adult (31–40 years)	serum	LC–MS/MS	Mayo Clinic Laboratories	Within RI
A4	711.1–768.4	Male	400–1500	Male	serum	LC–MS/MS	Mayo Clinic Laboratories	Within RI
	554.5–1473.7	FP, OV, LP	300–2000	Female	serum	LC–MS/MS	Mayo Clinic Laboratories	Within RI
	4616.8–9902.8	Pregnancy	888–6330	Pregnancy (40 weeks)	serum	LC–MS/MS	[9]	Partially above RI
T	3152.2–11555.2	Male	2400–9500	Male	serum	LC–MS/MS	Mayo Clinic Laboratories	Partially above RI

	167.1–322.7	FP, OV, LP	80–600	Female	serum	LC–MS/MS	Mayo Clinic Laboratories	Within RI
	1750.3–3859.6	Pregnancy	404–2538	Pregnancy (40 weeks)	serum	LC–MS/MS	[9]	Partially above RI
5α–DHT	266.9–314.1	Male	112–955	Male	serum	LC–MS/MS	Mayo Clinic Laboratories	Within RI
	94.8–169.8	FP, OV, LP	<=300	Female (20–55 years)	serum	LC–MS/MS	Mayo Clinic Laboratories	Within RI
B	374.5–14979.5	All Phases	530–15600	Adult	serum	LC–MS/MS	Mayo Clinic Laboratories	Partially below RI
F	34754.2–121846.2	All Phases	20000–140000	Adult	serum	LC–MS/MS	Mayo Clinic Laboratories	Within RI
E	12208.1–37344.0	All Phases	12000–35000	Adult	serum/plasma	LC–MS/MS	ARUP Laboratories	Partially above RI
DHEA–S	594442.8–701092.8	Male	570000–5220000	Male (31–40 years)	serum	IEA	Mayo Clinic Laboratories	Within RI
	310644.6–465278.1	FP, OV, LP	270000–2400000	Female (41–50 years)	serum	IEA	Mayo Clinic Laboratories	Within RI
	171632.9–207007.1	Pregnancy	54650–1092050	Pregnancy (32 weeks)	serum	LC–MS/MS	[10]	Within RI

References

(1) Rehm, K.; Hankele, A.-K.; Ulbrich, S. E.; Bigler, L. Quantification of Glucocorticoid and Progestogen Metabolites in Bovine Plasma, Skimmed Milk and Saliva by UHPLC-HR-MS with Polarity Switching. *Anal. Chim. Acta* **2024**, *1287*, 342118.

(2) Su, M.; Drotleff, B.; Janker, T.; Bürger, Z.; Kimmig, A.-C. S.; Derntl, B.; Lämmerhofer, M. Quantification of Endogenous Steroids and Hormonal Contraceptives in Human Plasma via Surrogate Calibration and UHPLC-MS/MS. *Anal. Chem.* **2025**, *97* (25), 13496–13503.

(3) Gazárková, T.; Vlčková, H. K.; Plachká, K.; Vagnerová, K.; Dubecová, D.; Klusoňová, P.; Pácha, J.; Svec, F.; Nováková, L. Comprehensive Targeted Profiling of Multiple Steroid Classes in Rodent Plasma Using Liquid Chromatography-Mass Spectrometry. *Anal. Chim. Acta* **2024**, *1332*, 343362.

(4) Andrieu, T.; du Toit, T.; Vogt, B.; Mueller, M. D.; Groessl, M. Parallel Targeted and Non-Targeted Quantitative Analysis of Steroids in Human Serum and Peritoneal Fluid by Liquid Chromatography High-Resolution Mass Spectrometry. *Anal. Bioanal. Chem.* **2022**, *414* (25), 7461–7472.

(5) Schiffer, L.; Shaheen, F.; Gilligan, L. C.; Storbeck, K.-H.; Hawley, J. M.; Keevil, B. G.; Arlt, W.; Taylor, A. E. Multi-Steroid Profiling by UHPLC-MS/MS with Post-Column Infusion of Ammonium Fluoride. *J. Chromatogr. B* **2022**, *1209*, 123413.

(6) Yuan, T.; Le, J.; Wang, S; Li, Y. An LC/MS/MS Method for Analyzing the Steroid Metabolome with High Accuracy and from Small Serum Samples. *J. Lipid Res.* **2020**, *61* (4), 580–586.

(7) Cools, T.; Wilson, K. S.; Li, D.; Vancsok, C.; Mulot, B.; Leclerc, A.; Kok, J.; Haapakoski, M.; Bertelsen, M. F.; Ochs, A.; Girling, S. J.; Zhou, Y.; Li, R.; Vanhaecke, L.; Wauters, J. Development and Validation of a Versatile Non-Invasive Urinary Steroidomics Method for Wildlife Biomonitoring. *Talanta* **2024**, *273*, 125924.

(8) Karvaly, G.; Kovács, K.; Gyarmatig, M.; Gerszi, D.; Nagy, S.; Jalal, D. A.; Tóth, Z.; Vasarhelyi, B.; Gyarmati, B. Reference Data on Estrogen Metabolome in Healthy Pregnancy. *Mol. Cell. Probes* **2024**, *74*, 101953.

(9) Lau, S. L.; Yuen, L. Y.; Ho, C. S.; Chan, M. H. M.; Ma, R. C. W.; Tam, W. H. Gestational Age-Specific Reference Intervals for Androgens in Pregnancy. *J. Clin. Endocrinol. Metab.* **2025**, *110* (1), 176–184.

(10) Soldin, O. P.; Guo, T.; Weiderpass, E.; Tractenberg, R. E.; Hilakivi-Clarke, L.; Soldin, S. J. Steroid Hormone Levels in Pregnancy and 1 Year Postpartum Using Isotope Dilution Tandem Mass Spectrometry. *Fertil. Steril.* **2005**, *84* (3), 701–710.
